# MicroRNA Regulatory Mechanisms on *Citrus sinensis* leaves to Magnesium-Deficiency

**DOI:** 10.3389/fpls.2016.00201

**Published:** 2016-03-04

**Authors:** Cui-Lan Ma, Yi-Ping Qi, Wei-Wei Liang, Lin-Tong Yang, Yi-Bin Lu, Peng Guo, Xin Ye, Li-Song Chen

**Affiliations:** ^1^Institute of Plant Nutritional Physiology and Molecular Biology, Fujian Agriculture and Forestry UniversityFuzhou, China; ^2^College of Life Science, Fujian Agriculture and Forestry UniversityFuzhou, China; ^3^College of Horticulture, Fujian Agriculture and Forestry UniversityFuzhou, China; ^4^Institute of Materia Medica, Fujian Academy of Medical SciencesFuzhou, China; ^5^College of Resource and Environmental Science, Fujian Agriculture and Forestry UniversityFuzhou, China; ^6^The Higher Educational Key Laboratory of Fujian Province for Soil Ecosystem Health and Regulation, Fujian Agriculture and Forestry UniversityFuzhou, China; ^7^Fujian Key Laboratory for Plant Molecular and Cell Biology, Fujian Agriculture and Forestry UniversityFuzhou, China

**Keywords:** Mg-deficiency, *Citrus sinensis*, Illumina sequencing, leaves, microRNA

## Abstract

Magnesium (Mg)-deficiency, which affects crop productivity and quality, widespreadly exists in many agricultural crops, including citrus. However, very limited data are available on Mg-deficiency-responsive microRNAs (miRNAs) in higher plants. Using Illumina sequencing, we isolated 75 (73 known and 2 novel) up- and 71 (64 known and 7 novel) down-regulated miRNAs from Mg-deficient *Citrus sinensis* leaves. In addition to the remarkable metabolic flexibility as indicated by the great alteration of *miRNA* expression, the adaptive responses of leaf miRNAs to Mg-deficiency might also involve the following several aspects: (*a*) up-regulating stress-related genes by down-regulating *miR164, miR7812, miR5742, miR3946*, and *miR5158*; (*b*) enhancing cell transport due to decreased expression of *miR3946* and *miR5158* and increased expression of *miR395, miR1077, miR1160*, and *miR8019*; (*c*) activating lipid metabolism-related genes by repressing *miR158, miR5256*, and *miR3946*; (*d*) inducing cell wall-related gene *expansin 8A* by repressing *miR779*; and (*e*) down-regulating the expression of genes involved in the maintenance of S, K and Cu by up-regulating *miR395* and *miR6426*. To conclude, we isolated some new known miRNAs (i.e., miR7812, miR8019, miR6218, miR1533, miR6426, miR5256, miR5742, miR5561, miR5158, and miR5818) responsive to nutrient deficiencies and found some candidate miRNAs that might contribute to Mg-deficiency tolerance. Therefore, our results not only provide novel information about the responses of plant to Mg-deficiency, but also are useful for obtaining the key miRNAs for plant Mg-deficiency tolerance.

## Introduction

Magnesium (Mg), which serves as a central component of the chlorophyll (Chl) molecule and as a cofactor and allosteric modulator for more than 300 enzymes including ribulose-1,5-bisphosphate carboxylase, ATPase, protein kinases, phosphatases and glutathione synthase (Cakmak and Kirkby, 2008), participates in many physiological and biochemical processes during plant growth and development including photosynthesis (Tang et al., 2012; Yang et al., 2012), respiration (Peng et al., 2015), organic acid metabolism (Yang et al., 2013), carbohydrate partitioning between source and sink organs (Cakmak et al., 1994; Yang et al., 2012), phloem export of sucrose (Cakmak and Kirkby, 2008), and reactive oxygen species (ROS) formation and scavenging (Cakmak and Kirkby, 2008; Yang et al., 2012). Mg-deficiency, which affects crop productivity and quality, widespreadly exists in many agricultural crops, including citrus (Tang et al., 2012; Verbruggen and Hermans, 2013). In China, Mg-deficiency often occurs in citrus orchards, and is responsible for the loss of yield and poor fruit quality (Ling et al., 2009). According to our investigation in 2011, up to 77.4 and 35.6% of “Guanximiyou” pummelo (*Citrus grandis*) orchards in Pinghe, Zhangzhou, China were deficient in soil exchange Mg and leaf Mg, respectively (Li et al., 2015).

Although the physiological and biochemical responses of plants to Mg-deficiency have been investigated in some detail in various plants (Cakmak and Kirkby, 2008; Hermans et al., 2013; Verbruggen and Hermans, 2013), very limited data are available on the molecular mechanisms of plant tolerance to Mg-deficiency until recently. Peng et al. (2015) isolated 59 up- and 31 down-regulated (19 up- and 12 down-regulated) proteins from Mg-deficient *Citrus sinensis* leaves (roots). In addition, two studies with *Arabidopsis* showed that the responses of global transcriptomics to Mg-deficiency were asynchronized, with a less number of differentially expressed genes after 4 or 8 h in leaves and after 28 h or 1 week in roots (Hermans et al., 2010a,b). Although stress-related gene expression programme largely occurs at the transcriptional level, the roles of post-transcriptional gene regulation have been recognized with the discovery of microRNAs (miRNAs) and small-interfering RNAs (siRNAs; Shukla et al., 2008). Approximately 21-nucleotide-long miRNAs generated from non-coding transcripts, one of the most abundant classes of small RNAs, are crucial post-transcriptional regulators of gene expression by repressing translation or directly degrading mRNAs in plants (Jones-Rhoades et al., 2006). MiRNAs have key roles in plant adaptations to nutrient deficiencies (Khraiwesh et al., 2012; Lu et al., 2014, 2015; Zeng et al., 2014; Paul et al., 2015). Plant *miR399* and *miR827*, which are specifically induced by phosphorus (P)-deficiency, play a role in the regulation of P homeostasis by down-regulating their target genes *UBC24* and *nitrogen (N) limitation adaptation* (*NLA*), respectively (Shukla et al., 2008; Hsieh et al., 2009). Also, many other miRNAs such as miR156, miR159, miR166, miR169, miR395, miR397, miR398, miR408, miR447, miR482, miR1510 and miR2109 are involved in plant response to P-limitation (Valdés-López et al., 2010; Hackenberg et al., 2013; Zhao et al., 2013; Paul et al., 2015).

Recently, Lu et al. (2014, 2015) investigated long-term B-deficiency-responsive miRNAs by Illumina sequencing and obtained 134 (112 known and 22 novel) and 172 (158 known and 14 novel) differentially expressed miRNAs from B-deficient *C. sinensis* roots and leaves, respectively, demonstrating the possible involvement of miRNAs in the tolerance of citrus plants to B-deficiency. It is worth noting that most of these B-deficiency-responsive miRNAs were identified only from B-deficient leaves or roots, only 22 miRNAs were identified from the both. Obviously, long-term B-deficiency-induced alterations of miRNA expression profiles greatly differed between leaves and roots.

In *Arabidopsis, miR857, miR408, miR398*, and *miR397*, which are up-regulated by copper (Cu)-deficiency, have been demonstrated to contribute to plant Cu homeostasis *via* negatively regulating nonessential Cu protein genes, thus saving Cu for other essential Cu proteins in Cu-deprived plants (Yamasaki et al., 2007; Abdel-Ghany and Pilon, 2008). Waters et al. (2012) reported that iron (Fe)-deficient *Arabidopsis* rosettes had more accumulation of Cu accompanied by decreased expression levels of *miR397a, miR398a*, and *miR398b/c*, indicating a link between Fe-deficiency and Cu homeostasis.

Many differentially expressed miRNAs (at least 27conserved families) have been isolated from N-deficient soybean, common bean, *Arabidopsis* and maize (Valdés-López et al., 2010; Liang et al., 2012; Zhao et al., 2012; Zeng et al., 2014). In *Arabidopsis, miR169* was greatly repressed and its target genes, *NFYA* (Nuclear Factor Y, subunit A) *family members*, were greatly up-regulated by N-deficiency. Transgenic *Arabidopsis* plants over-expressing *miR169a* had lower N level, and displayed less tolerance to N-deficiency than the wild type, indicating the possible roles of miR169 in helping plants to deal with N-starvation (Zhao et al., 2011).

*MiR395*, which targets two sulfur (S) metabolism-related genes [i.e., *ATP sulfurylases* (*APS*) and *sulfate transporter 2;1* (*SULTR2;1*)], was induced by S-deprivation. *MiR395*-over-expressing *Arabidopsis* exhibited remarkable down-regulation in mRNA levels of its two target genes, and had more accumulation of S in the shoot but not in the root. The *aps1-1 sultr2;1 APS4-RNAi* mutants displayed similar phenotypes to those of *miR395*-over-expressing plants. These authors concluded that *miR395*-mediated regulation of *APS* and *SULTR2;1* might play a crucial role in plant S homeostasis (Liang et al., 2010).

So far, many workers have investigated the roles of plant miRNAs in response to nutrient deficiencies. Most studies, however, have focused on P, B, N, Fe, and S deficiencies. Little is known about Mg-deficiency-responsive miRNAs in higher plants. In this study, we first sequenced two small RNA libraries from Mg-deficient and -sufficient (control) *C. sinensis* leaves, respectively, using Illumina sequencing in order to identify the Mg-deficiency-responsive miRNAs that might contribute to the tolerance of plants to Mg-deficiency.

## Materials and methods

### Plant materials and Mg treatments

This study was conducted at Fujian Agriculture and Forestry University (FAFU), Fuzhou, China (26°5′ N, 119°14′ E) with an average annual temperature of ca. 20°C and an average annual sunlight hours of ca. 1600 h. Plant culture and Mg treatments were performed according to Peng et al. (2015). Briefly, 15-week-old seedlings of “Xuegan” [*Citrus sinensis* (L.) Osbeck] in 6 L pots filled with fine river sand were grown in a greenhouse under natural photoperiod at FAFU. Each pot, which contained two seedlings, was irrigated every other day until saturated with nutrient solution containing 2.5 mM KNO_3_, 2.5 mM Ca(NO_3_)_2_, 0.5 mM KH_2_PO_4_, 10 μM H_3_BO_3_, 2 μM MnCl_2_, 2 μM ZnSO_4_, 0.5 μM CuSO_4_, 0.065 μM (NH_4_)_6_Mo_7_O_24_, 20 μM Fe-EDTA and 0 mM (Mg-deficiency) or 1 mM (Mg-sufficiency) MgSO_4_ for 16 weeks. Sulfur concentration was maintained at a constant level by using equivalent moles of Na_2_SO_4_ in replace of MgSO_4._ At the end of the experiment, fully-expanded (about 7 weeks old) leaves from different replicates and treatments were used for all the measurements. Leaves were collected at noon under full sun and immediately frozen in liquid N_2_, then stored at −80°C until extraction.

### Plant dry weight (DW) and leaf Mg concentration

At the end of the experiment, 9–10 plants per treatment from different pots were collected. Plant DW was measured after being dried at 70°C for 48 h. Leaf Mg concentration was assayed by atomic absorption spectroscopy after digested with 1 N HCl (Kushizaki, 1968).

### Leaf SRNA library construction and illumina sequencing

Equal amounts of frozen Mg-deficient or -sufficient leaves from five plants (one plant per pot) were mixed as a biological replicate. Total RNA was extracted from 0.1 g mixed frozen samples using TRIzol reagent (Invitrogen, Carlsbad, CA) following manufacturer's instructions. Mg-deficient and -sufficient leaf sRNA libraries were constructed according to Lu et al. (2014). Illumina sequencing was performed on a Solexa sequencer at the Beijing Genomics Institute (BGI), Shenzhen, China (Lu et al., 2014).

### SRNA annotation and MiRNA identification

Both sRNA annotation and miRNA identification were performed according to Lu et al. (2014). Briefly, software developed by the BGI was used to analyze the raw data from the Solexa sequencing. Clean reads were then used to calculate length distribution and common/specific sequences. Thereafter, the clear reads were mapped to *C. sinensis* genome (JGIversion 1.1, http://phytozome.jgi.doe.gov/pz/portal.html#!info?alias=Org_Csinensis) using SOAP, only perfectly mapped sequences were retained and analyzed further. RRNAs, tRNAs, snRNAs and snoRNAs were removed from the sRNAs sequences through BLASTn search using NCBI Genebank database (http://www.ncbi.nlm.nih.gov/blast/Blast.cgi/) and Rfam (12.0) database (http://www.sanger.ac.uk/resources/databases/rfam.html) using following program and parameters: blastall -p blastn -F F -e 0.01. The repeat associated RNA and piRNA were identified using tag2repeat and tag2piRNA (developed by BGI) respectively. SRNA tags were also aligned to exons and introns of mRNA to find the degraded fragments of mRNA. All annotations were summarized using tag2annotation software (developed by BGI) in the following order of preference: rRNA (Genbank > Rfam) > known miRNA > repeat > exon > intron. The remaining sequences were aligned with known plant miRNAs from miRBase 21 (http://www.mirbase.org/) with up to two mismatches. Reads that were not annotated were used to predict novel miRNAs using Mireap (http://sourceforge.net/projects/mireap/), a prediction software developed by the BGI, by exploring the secondary structure, the Dicer cleavage site and the minimum free energy of the unannotated small RNA tags which could be mapped to genome. Parameters were set as follows: minimal miRNA sequence length (18), maximal miRNA sequence length (25), minimal miRNA reference sequence length (20), maximal miRNA reference sequence length (23), maximal copy number of miRNAs on reference (20), maximal free energy allowed for a miRNA precursor (−18 kcal/mol), maximal space between miRNA and miRNA^*^ (300), minimal base pairs of miRNA and miRNA^*^ (16), maximal bulge of miRNA and miRNA^*^ (4), maximal asymmetry of miRNA/miRNA^*^ duplex (4) and flank sequence length of miRNA precursor (20; Lu et al., 2014). In addition, we used MTide (http://bis.zju.edu.cn/MTide; Zhang et al., 2015) and DNAMAN 8 (http://www.lynnon.com/pc/framepc.html) to predict novel miRNA. Only these miRNA candidates that were simultaneously predicted by the three softwares were considered to be novel miRNAs.

### Differential expression analysis of MiRNAs

Both the fold change between Mg-deficiency and -sufficiency and the *P*-value were calculated from the normalized expression of TPM (Wang et al., 2011). Normalized expression was calculated by the following formula: Normalized expression = Actual miRNA count/Total count of clean reads^*^1,000,000. The fold change between B-deficiency and control was calculated as: Fold-change = log_2_ (B-deficiency/Control). The *p*-value was calculated by the following formula:

$$\begin{array}{l} p\left(x|y\right)={\left(\frac{N_{2}}{N_{1}}\right)}^{y}{\frac{\left(x+y\right)!}{x!y!{\left(1+\frac{N_{2}}{N_{1}}\right)}^{\left(x+y+1\right)}}}_{D\left(y\geq y_{\max}|x\right)\;=\;\overset{\infty }{\underset{y\geq y_{\max}}{\sum }}p\left(y|x\right)}^{C\left(y\leq y_{\min}|x\right)\;=\;\overset{y\leq y_{\min}}{\underset{y=0}{\sum }}p\left(y|x\right)} \end{array}$$

A miRNA was considered differentially expressed when the miRNA had both a *P*-value of less than 0.01 and a fold change of more than 1.5 (Lu et al., 2014).

### Target prediction of MiRNAs

Target prediction of miRNAs was performed by RNAhybrid based on rules suggested by Allen et al. (2005) and Schwab et al. (2005): (*a*) no more than four mismatches between sRNA and target (G-U bases count as 0.5 mismatches); (*b*) no more than two adjacent mismatches in the miRNA/target duplex; (*c*) no adjacent mismatches in positions 2–12 of the miRNA/target duplex (5′ of miRNA); (*d*) no mismatches in positions 10–11 of miRNA/target duplex; (*e*) no more than 2.5 mismatches in positions 1–12 of the of the miRNA/target duplex (5′ of miRNA); and (*f*) minimum free energy (MFE) of the miRNA/target duplex should be >75% of the MFE of the miRNA bound to it's perfect complement.

### Functions of the potential targets of the differentially expressed MiRNAs

All targets of the differentially expressed miRNAs were mapped to GO terms in the database (http://www.geneontology.org/), and calculated gene numbers for each term. The GO results were expressed as three categories: biological process, molecular function and cellular component (Lu et al., 2014).

### Validation of MiRNA expression by stem-loop qRT-PCR

The analysis of miRNA expression was performed using stem-loop qRT-PCR method, stem-loop primers for reverse transcription and primers for qRT-PCR were listed in Table S1. Total RNA was reversetranscribed using Taqman® MicroRNA Reverse Transcription Kit (USA). SYBR® Premix Ex Taq™ II (Takara, Japan) kit was used for qRT-PCR. MiRNA special (forward) primers were designed according to the miRNA sequence but excluded the last six nucleotides at 3′ end of the miRNA. A 5′ extension of several nucleotides, which was chosen randomly and relatively GC-rich, was added to each forward primer to increase the melting temperature (Chen et al., 2005). All the primers were assigned to Primer Software Version 5.0 (PREMIER Biosoft International, USA) to assess their quality. For qRT-PCR, 20 μL reaction solution contained 10 μL ready-to-use SYBR® Premix Ex TaqTM II (Takara, Japan), 0.8 μL 10 μM miRNA forward primer, 0.8 μL 10 μM Uni-miR qPCR primer, 2 μL cDNA template and 6.4 μL dH_2_O. The cycling conditions were 60 s at 95°C, followed by 40 cycles of 95°C for 10 s, 60°C for 30 s. qRT-PCR was performed on the ABI 7500 Real Time System. Samples for qRT-PCR were run in three biological replicates with two technical replicates. Relative miRNA expression was calculated using ddCt algorithm. For the normalization of miRNA expression, *actin* (AEK97331.1) was used as an internal standard and the leaves from control plants were used as reference sample, which was set to 1.

### qRT-PCR analysis of MiRNA target gene expression

Total RNA was extracted from frozen Mg-sufficient and -deficient leaves using TRIzol reagent (Invitrogen, Carlsbad, CA) following manufacturer's instructions. The sequences of the F and R primers used were given in Table S2. qRT-PCR analysis of miRNA target gene expression was performed using an ABI 7500 Real Time System according to Lu et al. (2014). Samples for qRT-PCR were run in three biological replicates with two technical replicates.

### Experimental design and statistical analysis

There were 20 pot seedlings per treatment in a completely randomized design. Experiments were performed with 3 replicates except for plant DW (*n* = 9–10) and leaf Mg concentration (*n* = 5). The unpaired *t*-test was applied for comparison between two means.

## Results

### Plant growth and leaf Mg concentration

**Figure 1:**
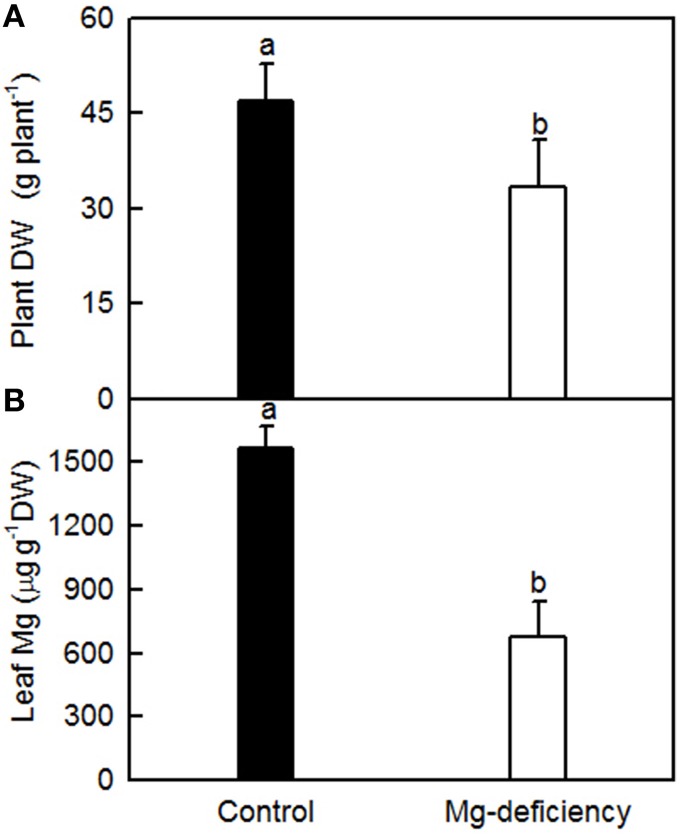
**Effects of Mg-deficiency on plant growth (A) and leaf Mg concentration (B)**. Bars represent mean ± SD (*n* = 5 for leaf Mg and 9–10 for plant DW). Different letters above the bars indicate a significant difference at *P* < 0.05.

As shown in Figure 1, 0 mM Mg-treated seedlings had lower plant DW and leaf Mg concentration than 1 mM Mg-treated ones, and leaf Mg concentration was much less than the normal range (Chapman, 1968). Visible Mg-deficient symptoms were observed only in 0 μM Mg-treated leaves (Figure S1). Thus, seedlings treated with 0 mM Mg are considered as Mg-deficient, and those treated with 1 mM Mg are considered as Mg-sufficient (control).

### Sequencing and analysis of two SRNA libraries from Mg-sufficient and -deficient citrus leaves

To isolate Mg-deficiency-responsive miRNAs, two sRNA libraries were constructed from leaves of *C. sinensis* seedlings submitted to 0 or 1 mM MgSO_4_ for 16 weeks, respectively. After being sequenced by a Solexa sequencer, we obtained 20,602,570 and 22,513,099 raw reads from Mg-sufficient and -deficient leaf libraries, respectively. These raw reads were cleaned by removing the contaminant reads like adaptors and low quality tags, thus leading to the generation of 20,328,011 (4,024,507) and 22,218,850 (4,480,037) clear reads (unique reads) from Mg-deficient and -deficient libraries, respectively (Table 1). Most of the clear sequences were within the range of 20–24 nt, which accounted for 94 and 96% of the total clear reads from Mg-deficient and -sufficient leaves, respectively. Reads with the length of 24 nt were at the most abundant, followed by the reads with length of 21, 22, 23, and 20 nt (Figure S2), as found for fruits of *C. sinensis* (Xu et al., 2010) and *Poncirus trifoliata* (Song et al., 2010), roots and leaves of *C. sinensis* (Lu et al., 2014, 2015), and flowers of *P. trifoliata* (Song et al., 2010). Therefore, the data of sRNA libraries obtained in this study are reliable. Compared with the controls, Mg-deficient leaves displayed less 21 and 24 nt clean reads and more 22 and 23 nt clean reads (Figure S2).

**Table 1:** **Statistical analysis of sRNA sequencing data from Mg-sufficient and -deficient leaves of *Citrus sinensis***.

	**Mg-sufficiency**		**Mg-deficiency**	
	**Unique sRNAs**	**Total sRNAs**	**Unique sRNAs**	**Total sRNAs**
Raw reads		20602570		22513099
Clear reads	4024507(100%)	20328011(100%)	4680037(100%)	22218850(100%)
Mapped to genomic	2057122(51.15%)	10424352(51.29%)	2498165(53.38%)	14966029(67.36%)
Exon antisense	43345(1.08%)	168476(0.83%)	36748(0.79%)	185172(0.83%)
Exon sense	73814(1.83%)	297258(1.46%)	78538(1.68%)	383020(1.72%)
Intron antisense	39066(1.01%)	241915(1.19%)	46841(1.00%)	320641(1.44%)
Intron sense	55378(1.38%)	433107(2.13%)	65216(1.39%)	716855(3.23%)
miRNA	42935(1.07%)	2280530(11.22%)	49819(1.06%)	4888886(22.00%)
rRNA	270955(6.73%)	7699010(37.87%)	157928(3.37%)	3063831(13.79%)
repeat	832(0.02%)	2139(0.01%)	1126(0.02%)	3516(0.02%)
snRNA	4548(0.11%)	15986(0.08%)	2727(0.06%)	7986(0.04%)
snoRNA	1228(0.03%)	2466(0.01%)	1011(0.02%)	2351(0.01%)
tRNA	19314(0.48%)	347898(1.71%)	20326(0.43%)	832644(3.75%)
Unannotated sRNAs	3473092(86.30%)	8839226(43.48%)	4219757(90.17%)	11813948(53.17%)

As shown in Table 1, 10,424,352 (2,057,122) and 14,966,029 (2,498,165) clean reads (unique reads) from Mg-sufficient and -deficient leaves, respectively were mapped to *C. sinensis* genome (JGIversion 1.1, http://phytozome.jgi.doe.gov/pz/portal.html#!info?alias=Org_Csinensis) using SOAP (Li et al., 2008). After removal of these annotated reads such as exon, intron, miRNA, rRNA, repeat regions, snRNA, shorn and tRNA, the remained unique read used for the prediction of novel miRNAs for Mg-sufficient and -deficient leaves were 3,473,092 and 4,219,757 reads, respectively.

### Isolation of known and novel mirnas in citrus leaves

We isolated 691 known miRNAs from the two libraries constructed from Mg-sufficient and -deficient leaves (Table S3). To compare the abundance of miRNAs in the two libraries, the count of reads was normalized to transcript per million (TPM). In this experiment, known miRNAs with normalized read-count less than ten in the two leaf libraries were not used for further analysis in order to avoid false results caused by the use of low expressed miRNAs (Chen et al., 2012; Lu et al., 2014). After these low expressed miRNAs being excluded, the remained 288 known miRNAs were used for further analysis (Table S4).

As shown in Table 1, the unannotated 3,473,092 and 4,219,757 unique clean reads from Mg-sufficient and -deficient leaf libraries, respectively were used to predict the novel miRNAs. Based on the criteria for annotation of plant miRNAs (Jones-Rhoades et al., 2006; Meyers et al., 2008), we obtained 113 novel miRNAs from the two libraries (Table S5). Like known miRNAs, only 34 novel miRNAs with normalized read-count more than 10 in Mg-sufficient and/or Mg-deficient libraries were used for the expression analysis (Table S6).

### Mg-deficiency-responsive MiRNAs in citrus leaves

A miRNA was considered differentially expressed when it had both a *P*-value of less than 0.01 and a fold-change of more than 1.5. According to the above criteria, we identified 75 (73 known and 2 novel) up-regulated and 71 (64 known and 7 novel) down-regulated miRNAs from Mg-deficient leaves. The strongest up-regulated known (novel) and down-regulated known (novel) miRNAs were miR5832 with a fold-change of 17.61 (novel_miR_96 with a fold-change of 17.75) and miR4351 with a fold-change of -14.66 (novel_miR_243 with a fold-change of -13.08), respectively (Tables S7, S8).

### Validation of illumina sequencing data by qRT-PCR

**Figure 2:**
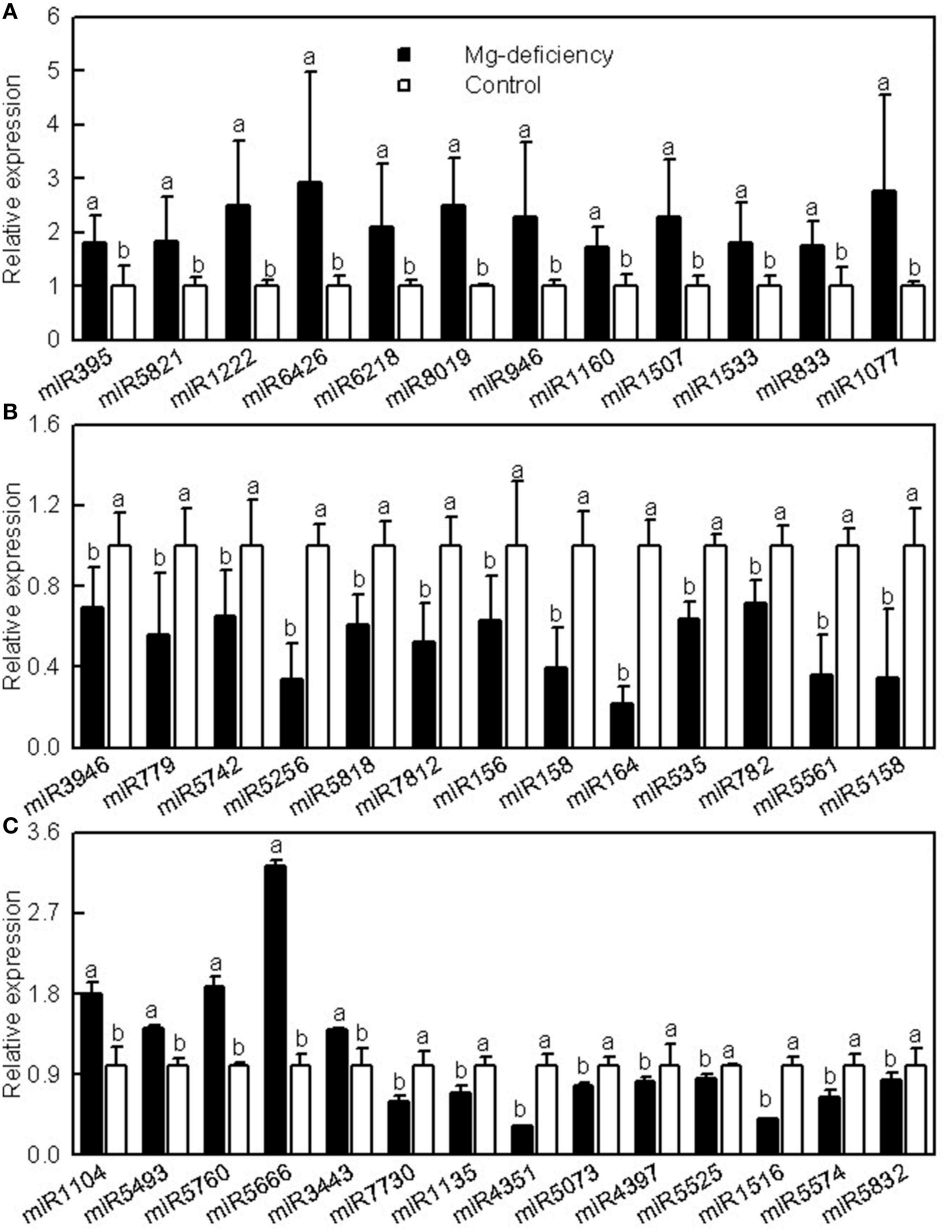
**Relative abundances of selected known miRNAs in Mg-deficient and -sufficient (control) leaves revealed by qRT-PCR**. Bars represent mean ± SD (*n* = 3). Significant differences were tested between control and Mg-deficient leaves for the same miRNA. Different letters above the bars indicate a significant difference at *P* < 0.05. All the values were expressed relative to the control leaves.

Since only one mixed sample of Mg-sufficient or -deficient leaf RNA was sequenced, the expression levels of 39 known miRNAs were analyzed using stem-loop qRT-PCR to validate the *miRNA* expression patterns revealed by Illumina sequencing. The expression levels of all these miRNAs except for *miR1222, miR7730*, and *miR5832* matched with the expression patterns obtained by Illumiona sequencing (Figure 2). Thus, the high-throughput sequencing allowed us to identify the Mg-deficiency-responsive miRNAs in citrus leaves.

### Identification of targets for Mg-deficiency-responsive MiRNAs and GO analysis

**Figure 3:**
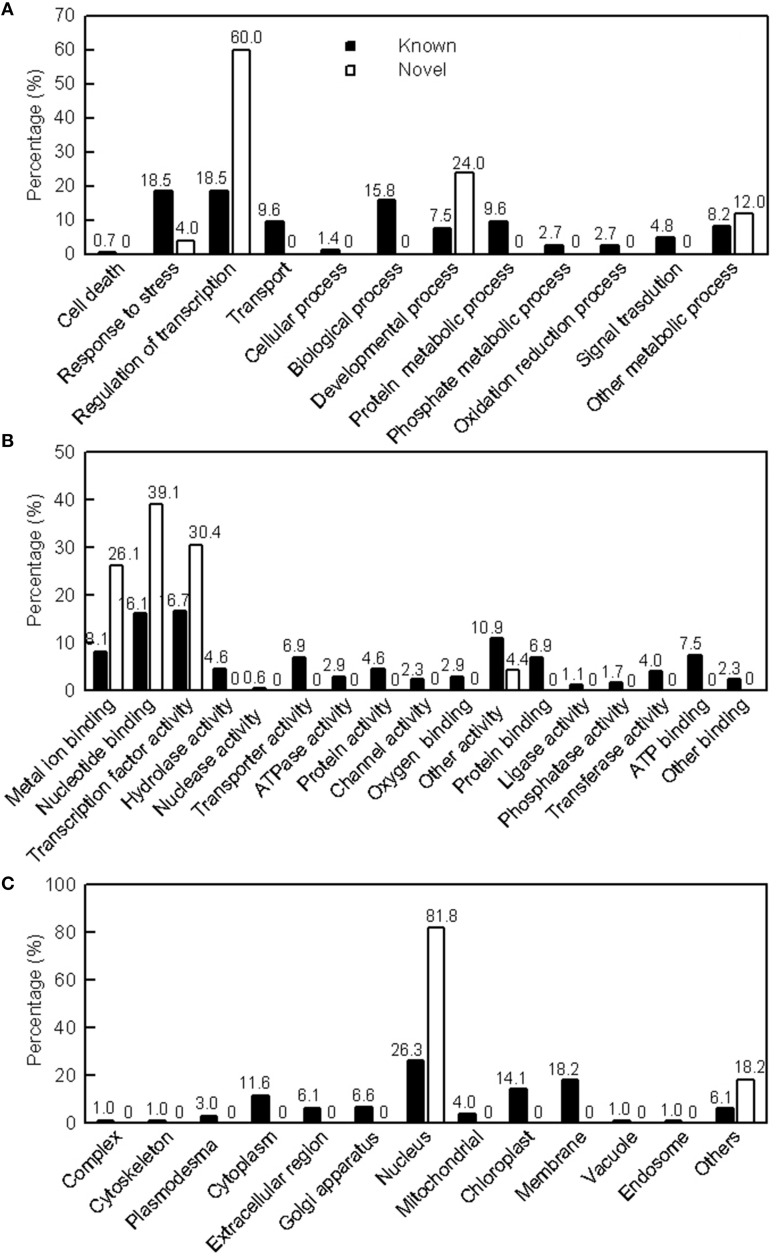
**GO of the predicted target genes for 57 (3) differentially expressed known (novel) miRNAs in *Citrus sinensis* leaves**. Categorization of miRNAs target genes was performed according to biological process **(A)**, molecular function **(B)**, and cellular component **(C)**.

We predicted 187 and 24 target genes from the 57 known and 3 novel differentially expressed miRNAs, respectively (Tables S9, S10). Based on the biological process, these target genes for known (novel) were mainly involved in response to stress, regulation of transcription, biological process, protein metabolic process and transport (regulation of transcription and developmental process; Figure 3A). Based on the molecular function, these target genes for the known and novel miRNAs genes were classified into 17 and four categories, respectively, the highest percentage of three categories for known miRNAs were transcription factor activity, nucleic acid binding and other activity (Figure 3B). Based on the cellular component, these target genes for the known and novel miRNAs were associated with 13 and two components, respectively. The most three GO terms for known miRNAs were nucleus, membrane and chloroplast (Figure 3C).

### qRT-PCR validation of target genes

We used qRT-PCR to assay the transcript levels of 77 genes targeted by 12 down- and 11 up-regulated miRNAs in order to verify the expression of the target genes and how the miRNAs regulate their target genes. As shown in Table 2, 56 (73%) target genes displayed the expected reverse changes in mRNA levels in Mg-deficient leaves with their corresponding miRNAs, demonstrating the possible roles of miRNAs in regulating gene expression under Mg-deficiency by cleaving mRNAs. However, 17 target genes had the similar change trends in transcript levels in Mg-deficient leaves with their corresponding miRNAs. The remaining four target genes were not detected in Mg-sufficient and -deficient *C. sinensis* leaves.

**Table 2:** **qRT-PCR relative expression of experimentally determined or predicted target genes of selected miRNAs**.

**miRNA**	**Fold change of miRNA**	**Accession**	**Homology**	**Target genes**	**Potential roles**	**Relative change of target genes**
miR164	−2.38730699^**^	orange1.1g030909m	AT1G56010.2	NAC domain containing protein 1	Transcription	1.4372^**^
		orange1.1g047710m	AT5G53950.1	NAC (No Apical Meristem) domain transcriptional regulator superfamily protein	Transcription	0.6610^**^
		orange1.1g017827m	AT5G61430.1	NAC domain containing protein 100	Transcription	2.9265^**^
miR158	−6.05735341^**^	orange1.1g001709m	AT3G07400	Lipase class 3 family protein	Lipid metabolism	4.1384^**^
		orange1.1g002569m	AT5G63020.1	Disease resistance protein (CC-NBS-LRR class) family	Disease, virulence and defense	3.2507^**^
		orange1.1g038105m	AT1G12220.1	Disease resistance protein (CC-NBS-LRR class) family	Disease, virulence and defense	1.6141^**^
		orange1.1g041843m	AT1G12280.1	LRR and NB-ARC domains-containing disease resistance protein	Disease, virulence and defense	0.7305^**^
miR833	5.7705023^**^	orange1.1g047519m	AT1G45616.1	Receptor like protein 6	Disease, virulence and defense	0.5503^**^
miR1507	1.99337925^**^	orange1.1g034576m	AT3G14470.1	NB-ARC domain-containing disease resistance protein	Disease, virulence and defense	0.1031^**^
		orange1.1g042037m	AT3G14460.1	LRR and NB-ARC domains-containing disease resistance protein	Disease, virulence and defense	0.5256^**^
		orange1.1g045522m	AT3G50950.2	HOPZ-ACTIVATED RESISTANCE 1	Disease, virulence and defense	1.6070^**^
miR156	−1.91583036^**^	orange1.1g016971m	AT5G50570.2	Squamosa promoter-binding protein-like (SBP domain) transcription factor family protein	Transcription	ND
		orange1.1g021420m	AT5G50670.1	Squamosa promoter-binding protein-like (SBP domain) transcription factor family protein	Transcription	1.3295^**^
		orange1.1g008680m	AT1G69170.1	Squamosa promoter-binding protein-like (SBP domain) transcription factor family protein	Transcription	2.0745^**^
		orange1.1g009653m	AT1G69170.1	Squamosa promoter-binding protein-like (SBP domain) transcription factor family protein	Transcription	1.5065^**^
		orange1.1g011640m	AT5G43270.2	Squamosa promoter binding protein-like 2	Transcription	1.4202^**^
		orange1.1g011651m	AT5G43270.3	Squamosa promoter binding protein-like 2	Transcription	ND
		orange1.1g032310m	AT2G33810.1	Squamosa promoter binding protein-like 3	Transcription	3.4314^**^
		orange1.1g029650m	AT1G53160.1	Squamosa promoter binding protein-like 4	Transcription	2.0105^**^
		orange1.1g032937m	AT3G15270.1	Squamosa promoter binding protein-like 5	Transcription	0.8213^**^
		orange1.1g046416m	AT2G42200.1	Squamosa promoter binding protein-like 9	Transcription	3.8189^**^
		orange1.1g030599m	AT3G60030.1	Squamosa promoter-binding protein-like 12	Transcription	1.1053^*^
miR7812	−7.07931495^**^	orange1.1g017621m	AT4G08850.1	Leucine-rich repeat receptor-like protein kinase family protein	Transmembrane signal transduction	0.7175^**^
		orange1.1g038769m	AT3G24503.1	Aldehyde dehydrogenase 2C4	Stress response	3.2200^**^
miR5821	10.86451118^**^	orange1.1g045278m	AT1G33060.2	NAC 014	Transcription	0.4220^**^
		orange1.1g022991m	AT1G71190.1	Senescence associated gene 18		1.3523^**^
		orange1.1g013216m	AT4G38220.2	Peptidase M20/M25/M40 family protein	Proteolytic degradation	0.4598^**^
		orange1.1g013368m	AT4G38220.1	Peptidase M20/M25/M40 family protein	Proteolytic degradation	0.9147^**^
		orange1.1g046783m	AT2G04620.1	Cation efflux family protein	Transport	0.5710^**^
miR395	10.30345436^**^	orange1.1g005583m	AT3G02050.1	K^+^ uptake transporter 3	Transport	0.9674^*^
		orange1.1g014749m	AT2G34250.1	SecY protein transport family protein	Transport	1.5667^**^
			AT3G22890	APS1	Sulfur metabolism	1.5017^**^
			AT4G14680	APS3	Sulfur metabolism	ND
			AT5G43780	APS4	Sulfur metabolism	0.8727^**^
			AT5G1018	SULTR2;1	Transport	0.7439^**^
miR1077	11.84568538^**^	orange1.1g014749m	AT2G34250.1	SecY protein transport family protein	Transport	1.7085^**^
miR946	10.29281625^**^	orange1.1g005467m	AT1G70610.1	Transporter associated with antigen processing protein 1	Transport	0.4222^**^
miR1160	5.35880943^**^	orange1.1g005451m	AT4G11440.1	Mitochondrial substrate carrier family protein	Transport	0.8227^**^
		orange1.1g004285m	AT3G23430.1	Phosphate 1	Transport	1.1364^**^
miR8019	6.3871206^**^	orange1.1g010016m	AT4G27500.1	Proton pump interactor 1	Transport	2.5270^**^
miR6218	3.83981303^**^	orange1.1g005203m	AT1G70610.1	Transporter associated with antigen processing protein 1	Transport	0.7108^**^
		orange1.1g003633m	AT2G32400.1	Glutamate receptor 5	Transport	0.8682^**^
miR1533	4.30631516^**^	orange1.1g007444m	AT1G08520.1	ALBINA 1	Chl biosynthesis and breakdown	0.6732^*^
miR6426	4.30631516^**^	orange1.1g037454m	AT5G60020.1	Laccase 17	Cu homeostasis	0.8741^**^
		orange1.1g010327m	AT5G24120.1	Sigma factor E	Transcription	0.8483^**^
miR3946	−2.16322268^**^	orange1.1g017665m	AT3G04070.1	NAC domain containing protein 47	Transcription	0.8570^**^
		orange1.1g013752m	AT2G46810.1	Basic helix-loop-helix (bHLH) DNA-binding superfamily protein	Transcription	9.9306^**^
		orange1.1g014958m	AT3G61950.1	Basic helix-loop-helix (bHLH) DNA-binding superfamily protein	Transcription	0.6579^**^
		orange1.1g005518m	AT2G35940.1	BEL1-like homeodomain 1	Transcription	1.2743^**^
		orange1.1g025914m	AT4G13040.1	Integrase-type DNA-binding superfamily protein	Transcription	1.8616^**^
		orange1.1g024507m	AT5G32450.1	RNA binding (RRM/RBD/RNP motifs) family protein	Transcription	1.1601^*^
		orange1.1g025497m	AT5G65430.1	General regulatory factor 8	Signal transduction	3.6019^**^
		orange1.1g011991m	AT2G43850.1	Integrin-linked protein kinase family	MAPKKK	6.0692^**^
		orange1.1g006091m	AT5G24300.2	Glycogen/starch synthases, ADP-glucose type	Starch synthase	0.6423^**^
		orange1.1g009139m	AT5G24300.1	Glycogen/starch synthases, ADP-glucose type	Starch synthase	1.4300^**^
		orange1.1g010449m	AT5G08570.1	Pyruvate kinase family protein	Organic acid metabolism	0.7370^**^
		orange1.1g002698m	AT2G42600.1	Phosphoenolpyruvate carboxylase 2	Organic acid metabolism	2.1231^**^
		orange1.1g007773m	AT5G24240.1	Phosphatidylinositol 3- and 4-kinase; Ubiquitin family protein	Lipid metabolism	1.2461^**^
		orange1.1g002776m	AT3G22400.1	PLAT/LH2 domain-containing lipoxygenase family protein	Lipid metabolism	ND
		orange1.1g016142m	AT2G01170.1	Bidirectional amino acid transporter 1	Transport	5.4132^**^
		orange1.1g030941m	AT3G16640.1	Translationally controlled tumor protein		0.7158^**^
miR535	−1.56870368^**^	orange1.1g009840m	AT5G24910.1	Cytochrome P450, family 714, subfamily A, polypeptide 1	Metabolism	1.8899^**^
miR5256	−3.19897021^**^	orange1.1g004233m	AT4G35790.2	Phospholipase D delta	Lipid metabolism	2.3193
miR5742	−2.88770785^**^	orange1.1g009718m	AT5G20890.1	TCP-1/cpn60 chaperonin family protein	Protein folding and stabilizatio	1.9777^**^
		orange1.1g041155m	AT2G34930.1	Disease resistance family protein/LRR family protein	Disease resistance family	1.6675^**^
miR5561	−7.70753451^**^	orange1.1g018677m	AT4G36730.1	G-box binding factor 1	Transcription	2.5652^**^
		orange1.1g019071m	AT4G36730.2	G-box binding factor 1	Transcription	1.7283^**^
miR5158	−5.92521928^**^	orange1.1g043878m	AT5G37930.1	Protein with RING/U-box and TRAF-like domains	Transcription	1.9307^**^
		orange1.1g042649m	AT4G21330.1	Basic helix-loop-helix (bHLH) DNA-binding superfamily protein	Transcription	2.7431^**^
miR5818	−4.95063483^**^	orange1.1g001860m	AT4G27190.1	NB-ARC domain-containing disease resistance protein	Disease, virulence and defense	3.9827^**^
miR779	−8.10749886^**^	orange1.1g027903m	AT3G63120.1	Cyclin p1;1	Cell cycle and DNA processing	3.9679^**^
		orange1.1g044779m	AT2G38290.1	Ammonium transporter 2	Transport	7.1508^**^
		orange1.1g041074m	AT2G38290.1	Ammonium transporter 2	Transport	7.9240^**^
		orange1.1g042791m	AT3G14470.1	NB-ARC domain-containing disease resistance protein	Disease, virulence and defense	2.2157^**^
		orange1.1g001921m	AT3G45630.1	RNA binding (RRM/RBD/RNP motifs) family protein	Transcription	9.5707^**^
		orange1.1g025347m	AT2G40610.1	Expansin A8	Cell wall	21.0361^*^
		orange1.1g045028m	AT1G73660.1	Protein tyrosine kinase family protein	MAPKKK	0.7103^**^

Both fold change of miRNAs and relative change of target genes are the ratio of Mg-deficient to -sufficient leaves.

The value for relative change of target gene was an average of three biological replicates with two technical replicates; Target genes that had the expected changes in mRNA levels were marked in bold and blue.

* and

** indicate a significant difference at P < 0.05 and P < 0.01, respectively. ND, not detected.

## Discussion

In addition to their involvement in plant growth and development, evidence in *Arabidopsis, C. sinensis*, barley (*Hordeum vulgare*), soybean (*Glycine max*), white lupin (*Lupinus albus*), common bean (*Phaseolus vulgaris*), rapeseed (*Brassica napus*), tomato (*Solanum lycopersicum*), maize (*Zea mays*), and wheat (*Triticum aestivum*) shows that miRNAs play key roles in the adaptations of plants to P, Cu, Fe, N, and B deficiencies (Hsieh et al., 2009; Kong and Yang, 2010; Liang et al., 2010, 2012; Valdés-López et al., 2010; Zhao et al., 2012, 2013; Hackenberg et al., 2013; Lu et al., 2014, 2015; Zeng et al., 2014; Paul et al., 2015). Here, we isolated 137 known and nine novel Mg-deficiency-responsive miRNAs from *C. sinensis* leaves, respectively (Tables S7, S8), demonstrating the possible involvement of miRNAs in the tolerance of plants to Mg-deficiency.

The expression level of *miR164* was decreased in Mg-deficient leaves (Table 2), as found for transient low nitrate-stressed maize leaves (Xu et al., 2011) and water stressed cassava (*Manihot esculenta*) leaves (Phookaew et al., 2014). As expected, the expression of its target genes (*NAC domain containing protein 1* and *NAC domain containing protein 100*) was induced in Mg-deficient leaves (Table 2). Transgenic rice over-expressing *NAC1* and *NAC6* displayed higher drought and salt tolerance (Hu et al., 2006; Nakashima et al., 2007), and *SINAC4*-RNAi tomato plants were more sensitive to drought and salt stress (Zhu et al., 2014). Therefore, Mg-deficiency-induced down-regulation of leaf *miR164* might play a role in the tolerance of plants to Mg-deficiency by enhancing the expression of *NAC*. However, the expression of *NAC domain containing protein 47* targeted by miR3946 and *NAC 014* targeted by miR5821 was down-regulated in Mg-deficient leaves. Xu et al. (2011) observed that the expression level of *miR164* in maize leaves increased in response to chronic N limitation, concluding that miR164 might function in remobilizing the N from old to new leaves *via* boost senescence due to decreased expression of *NAC* under N limitation.

The expression level of *miR158* was lower in Mg-deficient leaves than in controls (Table 2). Similar results have been obtained on N-deficient *Arabidopsis* seedlings (Liang et al., 2012), B-deficient *C. sinensis* roots (Lu et al., 2014) and leaves (Lu et al., 2015). The observed lower expression level of *miR158* indicated that its target genes might be induced in these leaves. In fact, the expression of all target genes (i.e., one *lipase class 3 family protein* and two *disease resistance protein (CC-NBS-LRR class) family*) were up-regulated in Mg-deficient citrus leaves except for *LRR and NB-ARC domains-containing disease resistance*. Also, the expression levels of *HOPZ-ACTIVATED RESISTANCE 1* targeted by miR1507, *disease resistance family protein/LRR family protein* targeted by miR5742, and *NB-ARC domain-containing disease resistance protein* (AT4G27190.1 and AT3G14470.1) targeted by miR5818 and miR779 were increased in Mg-deficient leaves (Table 2). These implied that the disease resistance might be enhanced in Mg-deficient leaves. This agrees with our results that Mg-deficient citrus leaves had higher concentrations of K and Ca (Xu, 2015), which play a role in plant disease resistance (Amtmann et al., 2008; Huber and Jones, 2013). However, the expression levels of *receptor like proteins 6* targeted by miR833, *LRR and NB-ARC domains-containing disease resistance protein* and *NB-ARC domain-containing disease resistance protein* targeted by miR1507 were decreased in Mg-deficient leaves (Table 2).

MiR156, which targets a series of squamosa promoter binding protein-like (SPL) genes, determines plastochron length by regulating SPL levels (Wang et al., 2008). As shown in Table 2, most of the target genes showed expected reverse changes in mRNA levels in Mg-deficient leaves compared with miR156. Transgenic *Arabidopsis* over-expressing *miR156h* displayed enhanced rate of leaf initiation (Stief et al., 2014). A similar effect was detected in the *spl9 spl15* double mutant (Wang et al., 2008). The observed down-regulation of *miR156* in Mg-deficient leaves indicated that the rate of leaf initiation might be decreased in Mg-deficient seedlings, thus decreasing leaf number and leaf DW (Peng et al., 2015). Study showed that increased miR156 activity resulted in high concentration of anthocyanins, while decreased miR156 activity led to the accumulation of flavonols (Gou et al., 2011). Therefore, Mg-deficient citrus leaves might have less accumulation of anthocyanin and more accumulation of flavonols due to decreased abundance of miR156 (Table 2).

*MiR7812* was repressed and its target gene *aldehyde dehydrogenase (ALDH) 2C4* was induced in Mg-deficient leaves (Table 2). Aldehyde dehydrogenases, which catalyze the oxidation of aldehydes arising from reactions of ROS with lipids and proteins to carboxylic acids, function in the detoxification of aldehydes generated in plants exposed to abiotic stress. Over-expression of *ALDH3I1* and *ALDH7B4* in *Arabidopsis* increased tolerance to abiotic stresses and protected plants against lipid peroxidation and oxidative stress (Kotchoni et al., 2006). Transgenic *Arabidopsis* over-expressing a stress-inducible *ALDH* from *Arabidopsis* displayed enhanced stress tolerance, which was correlated with decreased accumulation of lipid peroxidation-derived reactive aldehydes compared to wild-type plants (Sunkar et al., 2003). Heat shock proteins (HSPs)/chaperones play crucial roles in protecting plants against stress by reestablishing normal protein conformation and thus cellular homeostasis. Our finding that Mg-deficiency increased leaf expression of *TCP-1/cpn60 chaperonin family protein* targeted by 5742 (Table 2) agrees with the report that the abundances of several HSPs were increased in Mg-deficient *C. sinensis* leaves (Peng et al., 2015).

Our finding that *miR395* was induced in Mg-deficient leaves (Table 2) agrees with the report that *miR395* in *Arabidopsis* leaves was enhanced by S-deficiency. MiR395 targets *APS1, APS2, APS4* and *sulfate transporter 2;1* (*SULTR2;1*). Their transcripts were decreased in transgenic *Arabidopsis* over-expressing *miR395* accompanied by increased accumulation of S in the shoot but not in the root. MiR395 might play a role in the regulation of plant S accumulation and allocation by targeting *APS* and *SULTR2;1* (Liang et al., 2010). As expected, the expression of *APS4* and *SULTR2;1* was down-regulated in Mg-deficient leaves (Table 2). Therefore, Mg-deficiency-induced up-regulation of leaf *miR395* might contribute to the homeostasis of S in plants, which agrees with our data that Mg-deficiency did not significantly affect S concentration in *C. sinensis* roots, stems and leaves (Xu, 2015). However, the expression of *APS1* was up-regulated in Mg-deficient leaves (Table 2). Also, *K*^+^ *uptake transporter 3* (*KUP3*), a target gene of miR395 was inhibited in Mg-deficient leaves (Table 2). Kim et al. (1998) showed that *AtKUP3* transcripts increased in K^+^-starved *Arabidopsis* roots. Because K concentration was higher in Mg-deficient *C. sinensis* roots, stems and leaves than in controls (Xu, 2015), the down-regulation of *KUP3* might provide an adaptive strategy of plants to Mg-deficiency by lowering K uptake, thus maintaining nutrient balance. Thus, it is reasonable to assume that miR395 played a role in the maintenance of S and K homeostasis.

Study showed that *Arabidopsis phosphate 1* (*PHO1*; At3g23430) played a role in the regulation of P homeostasis through the phosphate (Pi) loading to the xylem (Wang et al., 2004). The *pho1* mutant of *Arabidopsis* had ca. 95% less Pi and 50–75% less total P in shoots than wild-type plants (Poirier et al., 1991). As shown in Table 2, the expression levels of both *miR1160* and its target gene *PHO1* were enhanced in Mg-deficient leaves. The up-regulation of *PHO1* might be advantageous to alleviating Mg-deficiency-induced decrease in leaf P level, since Mg-deficient *C. sinensis* plants accumulated less P in roots, stems and leaves than controls (Xu, 2015).

Both leaf *miR8019* and its target gene: *proton pump interactor 1* (*PPI1*) were induced by Mg-deficiency (Table 2). Anzi et al. (2008) showed that PPI1 stimulated *in vitr*o activity of plasma membrane (PM) H^+^-ATPase. Thus, its activity might be enhanced in these leaves. This agrees with the previous reports that Fe-deficiency strongly increased PM H^+^-ATPase activity in cucumber roots (Dell'Orto et al., 2000), and that P-deficient soybean roots had increased PM H^+^-ATPase activity (Shen et al., 2006), because the concentrations of P and Fe were lower in Mg-deficient roots, stems and leaves than in controls (Xu, 2015).

*MiR1533* was up-regulated in Mg-deficient leaves and its target gene: *ALBINA 1* encoding the CHLD subunit of the Mg-chelatase involved in Chl biosynthesis, was down-regulated in these leaves (Table 2). This implied that Chl biosynthesis might be impaired, thus decreasing leaf Chl concentration and accelerating leaf senescence. This agrees with our data that *senescence associated gene 18* targeted by 5821 was up-regulated in Mg-deficient leaves (Table 2) and previous reports that Mg-deficient citrus leaves had lower Chl concentration (Tang et al., 2012; Yang et al., 2012).

Leaf *miR6426* was up-regulated and its target genes: *laccase 17* and *sigma factor E* (SIGE, SIG5) were down-regulated by Mg-deficiency (Table 2). The down-regulation of *laccase 17* might be advantageous to the maintenance of Cu homeostasis (Abdel-Ghany and Pilon, 2008), because Cu concentration was lower in Mg-deficient leaves than in controls (Xu, 2015). Studies showed that *laccase* down-regulation caused an increase in total phenolic content in poplar (Ranocha et al., 2002), and that Mg and Cu concentrations were negatively correlated with total phenols in beech (*Fagus sylvatica*) leaves (Påhlsson, 1989). Thus, it is reasonable to assume that Mg-deficient citrus had higher concentration of total phenols. Kanamaru and Tanaka (2004) demonstrated that *SIG5* was induced by various stresses and might contribute to the repair of damaged photosystem II (PSII) in higher plants. The down-regulation of *SIG5* indicated that PSII might be damaged in Mg-deficient leaves (Tang et al., 2012; Yang et al., 2012).

Mg-deficient leaves had lower expression level of *miR3946* and its target genes: *glycogen/starch synthases* (orange1.1g009139m) involved in starch biosynthesis and *phosphoenolpyruvate carboxylase (PEPC) 2* involved in organic acid metabolism were up-regulated in these leaves (Table 2). This agrees with our reports that Mg-deficient *C. sinensis* leaves had higher or similar concentrations of starch, glucose, fructose and sucrose, higher activities of pyruvate kinase (PK) and PEPC, and enhanced organic acid metabolism and respiration, which was considered to be an adaptive response to Mg-deficiency by providing energy to maintain the basic metabolic processes in Mg-deficient leaves with lower photosynthetic rate (Yang et al., 2012, 2013; Peng et al., 2015). Mg-deficiency-induced up-regulation of *phosphatidylinositol 3- and 4- kinase* involved in lipid metabolism agrees with our report that the abundances of two protein species involved in lipid metabolism were enhanced in Mg-deficient *C. sinensis* leaves, thus contributing to the tolerance of plants to Mg-deficiency (Peng et al., 2015). Similarly, the expression level of the lipid metabolism-related gene, *lipase class 3 family protein* targeted by miR158 was up-regulated in Mg-deficient leaves due to decreased expression levels of their miRNAs (Table 2). Therefore, lipid metabolism might be up-regulated in Mg-deficient leaves. BAT1, a bidirectional amino acid transporter in *Arabidopsis* could be involved in amino acid export from the phloem into sink tissues (Dündar and Bush, 2009). Recently, Ladwig et al. (2012) showed that *SIAR1*, encoding a BAT from *Arabidopsis*, played an important role in organic N allocation and particularly in amino acid homeostasis in developing siliques. Mutant alleles of *SIAR1* displayed more accumulation of anthocyanins and lower concentration of amino acids in the early stages of silique development. The up-regulation of *BAT1* agrees with the report that Mg-deficient spinach leaves displayed more accumulation of amino acids (Fischer et al., 1988). The basic helix-loop-helix (bHLH) proteins, a large superfamily of transcription factors (TFs) involved in DNA binding, play key roles in plant development and environmental responses (Hudson and Hudson, 2015). Huang et al. (2013) suggested that a *bHLH* of *P. trifoliata* might play a key role in cold tolerance *via* positively regulating peroxidase-mediated ROS scavenging. Vorwieger et al. (2007) reported that two *Arabidopsis bHLH TF* were strongly induced by Fe-deficiency. Long et al. (2010) observed that *POPEYE* encoding a bHLH TF was induced by Fe-deficiency, concluding that POPEYE might play a crucial role in *Arabidopsis* Fe homeostasis. The up-regulation of *bHLH DNA-binding superfamily protein* genes (AT2G46810.1 and AT4G21330.1) targeted by miR3946 and miR5158 in Mg-deficient leaves (Table 2) agrees with the report that Mg-deficiency decreased root, stem and leaf concentration of Fe (Xu, 2015).

The expression level of *miR535* was decreased in Mg-deficient leaves (Table 2), as obtained on drought potato leaves (Zhang et al., 2014) and *Xanthomonas axonopodis* pv. manihotis inoculated cassava leaves (Pérez-Quintero et al., 2012). MiR535 was predicted to target genes encoding cytochrome P450, family 714, subfamily A, polypeptide 1 in citrus (Table 2), disease resistance family protein, pectinesterase family protein, zinc ion binding, MLP-LIKE PROTEIN 423 and leucine-rich repeat transmembrane protein kinase, putative in cassava (Pérez-Quintero et al., 2012) and MYB domain-containing protein in potato (Zhang et al., 2014), which are involved in the regulation of various physiological processes. Thus, miR535 might play a role in the tolerance of plants to (a)biotic stresses.

As shown in Table 2, leaf *miR5561* was repressed and its target gene *G-box binding factor 1* (*GBF1*) was induced by Mg-deficiency. Smykowski et al. (2010) observed that *GBF1* negatively regulated the expression of *catalase2*, and that *gbf1 Arabidopsis* mutants had a delayed senescence phenotype and postponed expression of senescence-associated genes. Mg-deficiency-induced up-regulation of *GBF1* agrees with our above inference that Mg-deficiency accelerated leaf senescence, and that our report that Mg-deficient *C. sinensis* and *C. grandis* leaves had lower catalase (CAT) activity compared with controls (Yang et al., 2012).

*MiR779* was down-regulated and all its target genes except for *protein tyrosine kinase family protein* were induced in Mg-deficient leaves (Table 2). Expansins are essential for cell enlargement and cell wall loosening during many developmental processes in plants. Choi et al. (2003) showed that expansins participated in enhancing plant growth by mediating cell wall loosening. Evidence shows the involvement of expansions in plant tolerance to abiotic stresses including dehydration (Dai et al., 2012), heat (Xu et al., 2014) and salt (Lü et al., 2013). Thus, the up-regulation of *expansin A8* might have a positive role in plant Mg-deficiency tolerance. Through the interaction existing between Mg^2+^ and ${\text{NH}}_{4}^{+}$ in the absorption process, ${\text{NH}}_{4}^{+}$ levels would increase in Mg-deficient plants, thus leading to ${\text{NH}}_{4}^{+}$ toxicity, which could be reversed by increasing Mg supply (Lasa et al., 2000). *Ammonium transporter 1;1* (*AMT1;1*) transgenic rice had enhanced N use efficiency, growth and yield under optimal and suboptimal ${\text{NH}}_{4}^{+}$ conditions (Ranathunge et al., 2014). Thus, the up-regulation of *AMT2* might be an adaptive response of plants to Mg-deficiency. Similarly, the expression levels of the other transport-related genes targeted by miR395 and miR1077 (*SecY protein transport family protein*), miR1160 *(PHO1*), miR8019 (*PPI1*), and miR3946 (*BAT1*) were up-regulated in Mg-deficient leaves (Table 2). This agrees with our report that only two up-regulated protein species involved in protein transport were detected in Mg-deficient leaves, and that transport of proteins might be enhanced in Mg-deficient leaves (Peng et al., 2015). By contrast, the expression levels of *miR5821, miR395, miR946, miR1160*, and *miR6218* were increased in Mg-deficient leaves, and their some target genes related to transport [i.e., *cation efflux family protein, K*^+^ *uptake transporter 3* (KUT3), *transporter associated with antigen processing protein 1, mitochondrial substrate carrier family protein* and *glutamate receptor 5* (*GLR5*)] were down-regulated in these leaves. Therefore, the transport of some substances might be down-regulated in Mg-deficient leaves.

## Conclusions

**Figure 4:**
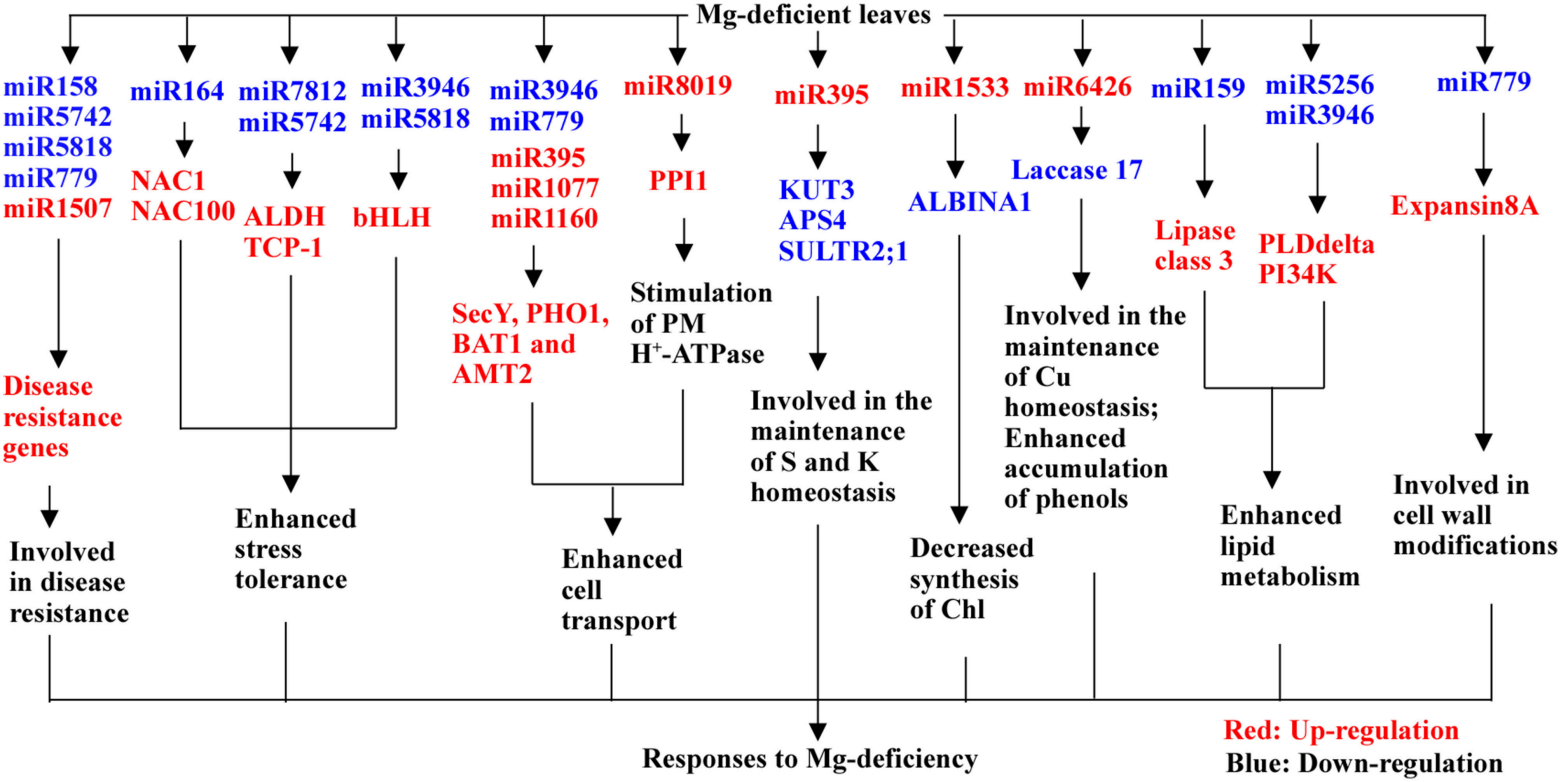
**A potential model for responses of *C. sinensis* leaf miRNAs to Mg-deficiency**. AMT2, ammonium transporter 2; BAT1, bidirectional amino acid transporter 1; PI34K, phosphatidylinositol 3- and 4- kinase; PLD delta, phospholipase D delta.

Using Illumina sequencing, we isolated 691 known and 113 novel miRNAs from Mg-deficient and -sufficient citrus leaves. A miRNA was considered differentially expressed when it had both a fold-change of more than 1.5 and a *P*-value of less than 0.01. Based on the two criteria, we obtained 75 (73 known and 2 novel) up-regulated and 71 (64 known and 7 novel) down-regulated miRNAs from Mg-deficient leaves. This indicated that *C. sinensis* leaves owned remarkable metabolic plasticity, which might contribute to Mg-deficiency tolerance of plants. As shown in Figure 4, a possible model for the responses of leaf miRNAs to Mg-deficiency was proposed *via* integrating the present findings with the data available on the previous reports. The adaptive responses of leaf miRNAs to Mg-deficiency might include following several aspects: (*a*) inducing stress-related genes by repressing *miR164, miR7812, miR5742, miR3946*, and *miR5158*; (*b*) up-regulating transport-related genes; (*c*) increasing the expression of genes related to lipid metabolism by inhibiting *miR158, miR5256*, and *miR3946* expression; (*d*) activating cell wall-related gene *expansis 8A* by down-regulating *miR779*; and (*e*) down-regulating the expression of genes involved in the maintenance of S, K and Cu by up-regulating *miR395* and *miR6426*. To sum up, we identified some new known miRNAs (i.e., miR7812, miR8019, miR6218, miR1533, miR6426, miR5256, miR5742, miR5561, miR5158, and miR5818) responsive to nutrient deficiencies and obtained some candidate miRNAs that might contribute to Mg-deficiency tolerance of *C. sinensis* plants. Further study is needed to elucidate the roles of these candidate miRNAs in responses to Mg-deficiency, which will be useful to us for obtaining the key miRNAs for plant Mg-deficiency tolerance.

## Data access

RNAseq are submitted to Gene Expression Omnibus (GEO) under accession number GSE75758 (http://www.ncbi.nlm.nih.gov/geo/query/acc.cgi?acc=GSE75758).

## Author contributions

CM carried out most of the experiments and drafted the manuscript; YQ participated in the design of the study. WL participated in data analysis. LY directed the study; YL participated in qRT-PCR analysis; PG participated in data analysis; XY determined leaf Mg concentration. LC designed and directed the study and revised the manuscript. All authors edited the manuscript.

## Funding

Our work was funded by the earmarked fund for China Agriculture Research System (No. CARS-27).

### Conflict of interest statement

The authors declare that the research was conducted in the absence of any commercial or financial relationships that could be construed as a potential conflict of interest.
