# Supplementary material for: MicroRNA Regulatory Mechanisms on Citrus sinensis leaves to Magnesium-Deficiency
Source: Front Plant Sci. 2016 Mar 4;7:201. doi: 10.3389/fpls.2016.00201 (PMC4778066; doi:10.3389/fpls.2016.00201)
Supplement: Supplementary file 1 [file DataSheet1.DOC]

**Supplementary Material**

**Table S1 | Stem loop primer sequences for qRT-PCR analysis of miRNAs.**

| miRNA | Sequence | RT-primer (5'→3') | Forward primer (5'→3') |
| --- | --- | --- | --- |
| miR395 | GTGAAGTGTTCGGATCGCC | GTCGTATCCAGTGCAGGGTCCGAGGTATTCGCACTGGATACGACGGCGAT | GAG TGC GTG AAG TGT TCG G |
| miR5821 | TGCGGAGGGATGGTGGGAG | GTCGTATCCAGTGCAGGGTCCGAGGTATTCGCACTGGATACGACCTCCCA | CAT GAA TGC GGA GGG ATG G |
| miR1222 | TTAAGAGAGGTCAGTGGTTCA | GTCGTATCCAGTGCAGGGTCCGAGGTATTCGCACTGGATACGACTGAACC | ACC GCC TTA AGA GAG GTC AGT |
| miR6426 | AGTGGAAGATGGAAGTGTAGA | GTCGTATCCAGTGCAGGGTCCGAGGTATTCGCACTGGATACGACTCTACA | GCA GCA GTG GAA GAT GGA AG |
| miR6218 | CGAAAAATCACGAAGACTTGC | GTCGTATCCAGTGCAGGGTCCGAGGTATTCGCACTGGATACGACGCAAGT | CGG CGC GAA AAA TCA CGA AG |
| miR8019 | AAAAGAAGACCCTTTTGAGCTTG | GTCGTATCCAGTGCAGGGTCCGAGGTATTCGCACTGGATACGACCAAGCT | GCG CGC AAA AGA AGA CCC TTT TG |
| miR946 | TGTGGAATAGAAGGGCTGAGT | GTCGTATCCAGTGCAGGGTCCGAGGTATTCGCACTGGATACGACACTCAG | GCC GCT GTG GAA TAG AAG GG |
| miR1160 | TGAAGGAAGCAGTAGTGGAT | GTCGTATCCAGTGCAGGGTCCGAGGTATTCGCACTGGATACGACATCCAC | GCG CCG TGA AGG AAG CAG TA |
| miR1507 | TCTTCATTCCACACGTCGTCT | GTCGTATCCAGTGCAGGGTCCGAGGTATTCGCACTGGATACGACAGACGA | CAG GCT CTT CAT TCC ACA CG |
| miR1533 | ATAATAAAAATAGATCATGTG | GTCGTATCCAGTGCAGGGTCCGAGGTATTCGCACTGGATACGACCACATG | CGCCGCGATAATAAAAATAGATCATGTG |
| miR833 | TGATTTGTTGATAATCGGCTCAGT | GTCGTATCCAGTGCAGGGTCCGAGGTATTCGCACTGGATACGACACTGAG | CGGTGATTTGTTGATAATCGGCTCAG |
| miR1077 | TTGAAGTGTTCGGATCGCGGC | GTCGTATCCAGTGCAGGGTCCGAGGTATTCGCACTGGATACGACGCCGCG | CGA GCG TTG AAG TGT TCG GAT |
| miR3946 | GTGTAGAGAGAGAGAGAGAGACAC | GTCGTATCCAGTGCAGGGTCCGAGGTATTCGCACTGGATACGACGTGTCT | CGG AAG TGT AGA GAG AGA GAG AG |
| miR779 | TCTGCTCATAGATTGTCTGCTCAT | GTCGTATCCAGTGCAGGGTCCGAGGTATTCGCACTGGATACGACATGAGC | CCGTCTGCTCATAGATTGTCTGCTC |
| miR5742 | CCAAATCAGATGGTCGCGGAT | GTCGTATCCAGTGCAGGGTCCGAGGTATTCGCACTGGATACGAC ATCCGC | AGA GCC CCA AAT CAG ATG GTC |
| miR5256 | ATATAGGATTATGAAGATTAA | GTCGTATCCAGTGCAGGGTCCGAGGTATTCGCACTGGATACGACTTAATC | GCGGCGATATAGGATTATGAAG |
| miR5818 | TCGAACTGAGAGGCACAGGTT | GTCGTATCCAGTGCAGGGTCCGAGGTATTCGCACTGGATACGACAACCTG | GCA GCT CGA ACT GAG AGG CA |
| miR7812 | ATGTTAGGAAATTGATGGGTG | GTCGTATCCAGTGCAGGGTCCGAGGTATTCGCACTGGATACGACCACCCA | GCC CGG CATG TTA GGA AAT TGA |
| miR156 | TTGACAGAAGAGAGTGAGCAC | GTCGTATCCAGTGCAGGGTCCGAGGTATTCGCACTGGATACGACGTGCTC | CTCGTTGACAGAAGAGAGTGAGCAC |
| miR158 | TTCAAAACTGTTAGACAAAGC | GTCGTATCCAGTGCAGGGTCCGAGGTATTCGCACTGGATACGACGCTTTG | GCG GCG GTT CAA AAC TGT TAG A |
| miR164 | TGGAGAAGCAGGGCACGTGCA | GTCGTATCCAGTGCAGGGTCCGAGGTATTCGCACTGGATACGACTGCACG | GAA GCT GGA GAA GCA GGG CA |
| miR535 | TGACAATGAGAGAGAGCACAC | GTCGTATCCAGTGCAGGGTCCGAGGTATTCGCACTGGATACGACGTGTGC | GCG CGT GAC AAT GAG AGA GA |
| miR782 | ACAAACAGAGTTGGATTTCTT | GTCGTATCCAGTGCAGGGTCCGAGGTATTCGCACTGGATACGACAAGAAA | GCG ACG ACA AAC AGA GTT GGA |
| miR5561 | CATAAGAGAGAGAGCATAGACAA | GTCGTATCCAGTGCAGGGTCCGAGGTATTCGCACTGGATACGACTTGTCT | GCGGCCATAAGAGAGAGAGCATA |
| miR5158 | TGAGACGGGATGAGATGAGAT | GTCGTATCCAGTGCAGGGTCCGAGGTATTCGCACTGGATACGACATCTCA | AGCGTGAGACGGGATGAGATGA |
| miR1104 | CGCAGCTGTTCGTCTTTCCC | GTCGTATCCAGTGCAGGGTCCGAGGTATTCGCACTGGATACGACGGGAAA | TTCTGCGCAGCTGTTCGTC |
| miR5493 | ACCGGGCTCGAGCGACGCGTG | GTCGTATCCAGTGCAGGGTCCGAGGTATTCGCACTGG ATACGACCACGCG | ATGTAACCGGGCTCGAGCGA |
| miR5760 | TGTTTAAGATAGTTTGTAAGGAT | GTCGTATCCAGTGCAGGGTCCGAGGTATTCGCACTGGATACGACATCCTT | GCGGCCTGTTTAAGATAGTTTGT |
| miR5666 | AGGGACATAGAGACATTTACT | GTCGTATCCAGTGCAGGGTCCGAGGTATTCGCACTGGATACGACAGTAAA | CAGCAGAGGGACATAGAGACA |
| miR3443 | GTGCGTTTGAGATTGAGGTT | GTCGTATCCAGTGCAGGGTCCGAGGTATTCGCACTGGATACGACAACCTC | CGGCGGTGCGTTTGAGATT |
| miR7730 | ATGAACACGGCACGATTGAAGTTAT | GTCGTATCCAGTGCAGGGTCCGAGGTATTCGCACTGGATACGACATAACT | GACGACATGAACACGGCACGATTGA |
| miR1135 | TTCGCTCAAGTAATTCTGACGGA | GTCGTATCCAGTGCAGGGTCCGAGGTATTCGCACTGGATACGACTCCGTC | GCGCCTTCGCTCAAGTAATTCT |
| miR4351 | ATTGGGAGTGTGCAGTGGGAGTGG | GTCGTATCCAGTGCAGGGTCCGAGGTATTCGCACTGGATACGACCCACTC | GCGATATTGGGAGTGTGCAGTGG |
| miR5073 | GATTGGTGAACGGCAGAAGTATTT | GTCGTATCCAGTGCAGGGTCCGAGGTATTCGCACTGGATACGACAAATAC | GCAGTGATTGGTGAACGGCAGAA |
| miR4397 | TGTCTAAGAGTGTGGCGACATTAC | GTCGTATCCAGTGCAGGGTCCGAGGTATTCGCACTGGATACGACGTAATG | GACGACTGTCTAAGAGTGTGGCGA |
| miR5525 | TCAATCCTTGTGGAGACGATCTGA | GTCGTATCCAGTGCAGGGTCCGAGGTATTCGCACTGGATACGACTCAGAT | GACGACTCAATCCTTGTGGAGACG |
| miR1516 | AAAGTTGGGACTTGAGGAGGCGGT | GTCGTATCCAGTGCAGGGTCCGAGGTATTCGCACTGGATACGACACCGCC | GACGCAAAGTTGGGACTTGAGGA |
| miR5574 | TTTAAATAGAACTCTAAAAGA | GTCGTATCCAGTGCAGGGTCCGAGGTATTCGCACTGGATACGACTCTTTT | GCCGCCTTTAAATAGAACTCT |
| miR5832 | TTGGCGGAGCGATTTGTC | GTCGTATCCAGTGCAGGGTCCGAGGTATTCGCACTGGATACGACGACAAA | GAACAGTTGGCGGAGCGA |
| Common reverse primer | | CAGTGCAGGGTCCGAGGT (5'→3') |  |
|  |  |  |  |
|  |  | Reverse primer (5'→3') | Forward primer (5'→3') |
| Actin |  | GCTTGGAGCAAGTGCTGTGATT | AGAACTATGAACTGCCTGATGGC |

**Table S2 | Specific primer pairs used for qRT-PCR expression analysis of selected miRNA target genes.**

| miRNA | Accession | Homology | Target genes | Forward primer(5'→3') | Reverse primer(5'→3') |
| --- | --- | --- | --- | --- | --- |
| miR164 | orange1.1g030909m | AT1G56010.2 | NAC domain containing protein 1 | AGGGCAGCCTTGTCGGTATG | GACGAAGCCTTAGGAGAATTAGCA |
|  | orange1.1g047710m | AT5G53950.1 | NAC (No Apical Meristem) domain transcriptional regulator superfamily protein | TCAATGCTGATGCTGGTGCC | GGTGGTGCGAAGAAGAAAGGAA |
|  | orange1.1g017827m | AT5G61430.1 | NAC domain containing protein 100 | AAGGGTTCTGGTGGGAAAAGG | GAGATGGGAGGAGGATTGTTGA |
| miR158 | orange1.1g001709m | AT3G07400 | Lipase class 3 family protein | GCAGCCAGCCCTTTCACTTC | CTGACAGCACCAGGCATAACATA |
|  | orange1.1g002569m | AT5G63020.1 | Disease resistance protein (CC-NBS-LRR class) family | GCAAAACTCCAGCGTCTTCG | TCTCCACCAGTATTCACCTCCAC |
|  | orange1.1g038105m | AT1G12220.1 | Disease resistance protein (CC-NBS-LRR class) family | ACTTTCGTTCTTGTGGTTGGTCTG | GGGCTGCTTATTATTCCCGTCA |
|  | orange1.1g041843m | AT1G12280.1 | LRR and NB-ARC domains-containing disease resistance protein | TGTAGGGAGGAGTCGATTGGTG | GGAAATAGACTTGAGGTTTGGAGC |
| miR833 | orange1.1g047519m | AT1G45616.1 | Receptor like protein 6 | GGGTTATGGATCTGGATTGGTG | GCCTGGAGGGTGATTATTTGAC |
| miR1507 | orange1.1g034576m | AT3G14470.1 | NB-ARC domain-containing disease resistance protein | TTCCATGACAGGTCAACCAC | TGAAACACTCCAGGGCAAT |
|  | orange1.1g042037m | AT3G14460.1 | LRR and NB-ARC domains-containing disease resistance protein | CGCTTTGTGGAAATCTGGAGTC | CAGTGAACGAATGTTGGTGGG |
|  | orange1.1g045522m | AT3G50950.2 | HOPZ-ACTIVATED RESISTANCE 1 | TCCTGCTTTCCTGCCACTTG | CGCCCTGGTTTCTCACTTCC |
| miR156 | orange1.1g016971m | AT5G50570.2 | Squamosa promoter-binding protein-like (SBP domain) transcription factor family protein | AATAAGTGTAAGGAGCCTGTTGCC | GAACCGTTGCTTCTGCCCAC |
|  | orange1.1g021420m | AT5G50670.1 | Squamosa promoter-binding protein-like (SBP domain) transcription factor family protein | ACCTGTTTGGAGTGGCATAGTCA | GAACTGCTTCCCTTCTTTGTCG |
|  | orange1.1g008680m | AT1G69170.1 | Squamosa promoter-binding protein-like (SBP domain) transcription factor family protein | AACGCAGCGACAGAAAGCAT | ACAGAAGAGGGTGAGCATAGCC |
|  | orange1.1g009653m | AT1G69170.1 | Squamosa promoter-binding protein-like (SBP domain) transcription factor family protein | GCAGTACCAAACACTAACGAGCAG | TTTCCCAAAGGTGTTGTGCC |
|  | orange1.1g011640m | AT5G43270.2 | Squamosa promoter binding protein-like 2 | AGGCTGTCTTCCTCACTCTATG | CCCCTTTGGCTGACTTTG |
|  | orange1.1g011651m | AT5G43270.3 | Squamosa promoter binding protein-like 2 | CGTGATGATTCCAATGGGCTCT | GACTTGTACTCGTGGGGTGCTG |
|  | orange1.1g032310m | AT2G33810.1 | Squamosa promoter binding protein-like 3 | GCCAAGGCTCCTGTTGTTCG | CGGCAACTCCTTTTGGTGTCA |
|  | orange1.1g029650m | AT1G53160.1 | Squamosa promoter binding protein-like 4 | CTCAGGTTGTGCTTATGGGTGG | CCGCAGACCAAATCCTTCAA |
|  | orange1.1g032937m | AT3G15270.1 | Squamosa promoter binding protein-like 5 | TTAACCGATGCAAGACGATACCAC | CAAACGCCTACGGCAACTCC |
|  | orange1.1g046416m | AT2G42200.1 | Squamosa promoter binding protein-like 9 | GAGGATGCTTCACAGGAGTTGC | TGAGTGACGGATTTGGTATGGG |
|  | orange1.1g030599m | AT3G60030.1 | Squamosa promoter-binding protein-like 12 | CTCAGGTTGTGCTTATGGGTGG | CCGCAGACCAAATCCTTCAA |
| miR7812 | orange1.1g017621m | AT4G08850.1 | Leucine-rich repeat receptor-like protein kinase family protein | TACTGTCCCAAATATGGCATCG | TTCACCCTTTTAGCCCAATCC |
|  | orange1.1g038769m | AT3G24503.1 | Aldehyde dehydrogenase 2C4 | GACCCGTGATGGTGTTGATGA | GGGGAATTGTGAATGGGAGTAA |
| miR5821 | orange1.1g045278m | AT1G33060.2 | NAC 014 | GCTATAACCGAGTGGGGTATGC | ATTGAAGTTGCTGCTGAGGGAT |
|  | orange1.1g022991m | AT1G71190.1 | Senescence associated gene 18 | TGCCTTAGTCCAGTTTGTTCCTTG | CCATTGCTTCTTCCACCTTTGC |
|  | orange1.1g013216m | AT4G38220.2 | Peptidase M20/M25/M40 family protein | GAAGGGGAGGTCGTTTCAGTTA | TCAGCATCAGTAGTCGGTGGG |
|  | orange1.1g013368m | AT4G38220.1 | Peptidase M20/M25/M40 family protein | ACAGATGCTCGCTACTTCCGTG | AGGCTTCATCCTTTGAGTGCTG |
|  | orange1.1g046783m | AT2G04620.1 | Cation efflux family protein | CCGACCACCATGAACCACTAA | GAGCAGGCAGGATCAGCAAC |
| miR395 | orange1.1g005583m | AT3G02050.1 | K+ uptake transporter 3 | CGGCAGGTGAGGTTTCAGTTA | CCTTCACATAGGAGTGACCCATTA |
|  | orange1.1g014749m | AT2G34250.1 | SecY protein transport family protein | TCTGTTAGGGAAGTGGAAGGAATC | GAGAAAAGAGCACAAGCAGTAAGC |
|  |  | AT3G22890 | APS1 | AGGTGCTTGAGGATGGTGTTCT | TGCCTTGAGTCTTGTCATACGCT |
|  |  | AT4G14680 | APS3 | GCGAATGAAACAGCACGAGAA | CCGACAATGTAGAAGTTAGCACCA |
|  |  | AT5G43780 | APS4 | GAACGCCTCAACATTCTACCCT | GCTTCTGAAACCCTTCCATTACC |
|  |  | AT5G1018 | SULTR2;1 | TCAGAGCTGGCTGTGAAAGTACA | GCAAACAACACCCCGAAGAA |
| miR1077 | orange1.1g014749m | AT2G34250.1 | SecY protein transport family protein | TCTGTTAGGGAAGTGGAAGGAATC | GAGAAAAGAGCACAAGCAGTAAGC |
| miR946 | orange1.1g005467m | AT1G70610.1 | Transporter associated with antigen processing protein 1 | ACCCTTGTGGATGATGATTTGC | GAGCTCAGCGTGATTACCTACCTC |
| miR1160 | orange1.1g005451m | AT4G11440.1 | Mitochondrial substrate carrier family protein | GCTGCCATCGCTAAAACCTG | GAGACCTTTCAAACCTTCACGCT |
|  | orange1.1g004285m | AT3G23430.1 | Phosphate 1 | CTTGGGTGGAGACCGTGATG | ATTCCAGTGCCCTCGTCGTA |
| miR8019 | orange1.1g010016m | AT4G27500.1 | Proton pump interactor 1 | AGATACCCTGCCAACCCAGAA | TGCTACTTTCGCCTCTTCCTTC |
| miR6218 | orange1.1g005203m | AT1G70610.1 | Transporter associated with antigen processing protein 1 | TGTGGGACAGGAACCAAAACTT | CATAACCACTGGGCAGCGAC |
|  | orange1.1g003633m | AT2G32400.1 | Glutamate receptor 5 | CTTTCCAACGGGATTCTCCAC | GACAGCCCTCTTTACAAAACCACT |
| miR1533 | orange1.1g007444m | AT1G08520.1 | ALBINA 1 | GGAGGAAGATCAGGAGGATGAGA | TTAATAGGACCCTTTGGGAGCA |
| miR6426 | orange1.1g037454m | AT5G60020.1 | Laccase 17 | ATTAGTCATGTCCCTGTCAATGCG | CAGAAACCGAGGTGCTACCCA |
|  | orange1.1g010327m | AT5G24120.1 | Sigma factor E | AAAGGTGATAGGACACTGGGAGAA | CATACCATGTGCTGGCGAAGA |
| miR782 | orange1.1g039969m | NM_001112290 | Protein disulfide isomerase (PDIL5-1) | CGTGAAAGTGAATGCGAAGGA | CCGGGGATGAATCAGGATAAG |
| miR3946 | orange1.1g017665m | AT3G04070.1 | NAC domain containing protein 47 | GAAGTCCACCAAGCCACAA | TGCTGCTCTAAAATCCCTGA |
|  | orange1.1g013752m | AT2G46810.1 | Basic helix-loop-helix (bHLH) DNA-binding superfamily protein | CTCAACACTCTTCGCTCCC | GCTGCTCCAGTTCCTTCAC |
|  | orange1.1g014958m | AT3G61950.1 | Basic helix-loop-helix (bHLH) DNA-binding superfamily protein | AATGAGAATGGGCACAAC | CAATGGCTTTCAACAACT |
|  | orange1.1g005518m | AT2G35940.1 | BEL1-like homeodomain 1 | AAGCAATGTGCCATCTAT | CTGAAAACCTTGGAGTGA |
|  | orange1.1g025914m | AT4G13040.1 | Integrase-type DNA-binding superfamily protein | GAAAGCGACATAGGAGGA | AGTGATTGAACGGCGAGT |
|  | orange1.1g024507m | AT5G32450.1 | RNA binding (RRM/RBD/RNP motifs) family protein | TCCTTTGACCGCAGAGTTG | TCCTTTGCAGTGAGGTTTGA |
|  | orange1.1g025497m | AT5G65430.1 | General regulatory factor 8 | AAGGAGGAGGGGAGGAAGA | TTGGACTCACTCGCCGTAG |
|  | orange1.1g011991m | AT2G43850.1 | Integrin-linked protein kinase family | GGATGCCTACTCTTTCGG | TCTTTCCACCAACCCTGT |
|  | orange1.1g006091m | AT5G24300.2 | Glycogen/starch synthases, ADP-glucose type | AGTATCGTGGTTGGGTTG | GCTTTCTTCTGATAGTGGC |
|  | orange1.1g009139m | AT5G24300.1 | Glycogen/starch synthases, ADP-glucose type | CAAGATTGCTTTGCAGAAGG | TCCAACCCAACCACGATAC |
|  | orange1.1g010449m | AT5G08570.1 | Pyruvate kinase family protein | TCCAATCCTATCTGTAGTTGTCCC | CACCCTCCAGTATCACCTCTG |
|  | orange1.1g002698m | AT2G42600.1 | Phosphoenolpyruvate carboxylase 2 | AAATGGAGTATGGTCGGA | TAAATGGAACCTCGTCTG |
|  | orange1.1g007773m | AT5G24240.1 | Phosphatidylinositol 3- and 4-kinase; Ubiquitin family protein | GGTTACTGCTTGCCCTAT | ATCTTTCAGCGTTTTCCT |
|  | orange1.1g002776m | AT3G22400.1 | PLAT/LH2 domain-containing lipoxygenase family protein | CAACAGACCAACCGTAAG | TGAGTGCCGTGATAAAAT |
|  | orange1.1g016142m | AT2G01170.1 | Bidirectional amino acid transporter 1 | CACCCGAAGCACAAAGTT | GGGCAAAAGAAAGACTGAAC |
|  | orange1.1g030941m | AT3G16640.1 | Translationally controlled tumor protein | TGAGGGAAAGTGGGTTGT | TGCTCCTGTAGCCTGAAT |
| miR535 | orange1.1g009840m | AT5G24910.1 | Cytochrome P450, family 714, subfamily A, polypeptide 1 | CTTGAAAACGGTGACTATG | AATGTTCCCGATTTGAGT |
| miR5256 | orange1.1g004233m | AT4G35790.2 | Phospholipase D delta | TGCCAAGGGGATGATAGT | ATGCGGCTGATAAGAACC |
| miR5210 | orange1.1g029519m | AT5G44080.1 | Basic-leucine zipper (bZIP) transcription factor family protein | GCGAACGAGAACGACCAG | TCACCGCCAGCACTATCC |
| miR5742 | orange1.1g009718m | AT5G20890.1 | TCP-1/cpn60 chaperonin family protein | GCAGGGTTTTGTTTGGAGG | CAGCAATGGTTGTTGGAATG |
|  | orange1.1g041155m | AT2G34930.1 | Disease resistance family protein/LRR family protein | AGGCGGAATGTGAGGAGC | GCACGACTGAGTGGGGTT |
| miR5561 | orange1.1g018677m | AT4G36730.1 | G-box binding factor 1 | TGCTCCCGCTGGCATAAT | GCTTGCGTAACCTTGACCTT |
|  | orange1.1g019071m | AT4G36730.2 | G-box binding factor 1 | TGCTCCCGCTGGCATAAT | GCTTGCGTAACCTTGACCTT |
| miR5158 | orange1.1g043878m | AT5G37930.1 | Protein with RING/U-box and TRAF-like domains | ACACTCCGATCATTAGTCC | TCATCTCCTCAGCAGCAT |
|  | orange1.1g042649m | AT4G21330.1 | Basic helix-loop-helix (bHLH) DNA-binding superfamily protein | TCACGATTTCTTCACTTCT | CGTTTGGATTGTCACATA |
| miR5818 | orange1.1g001860m | AT4G27190.1 | NB-ARC domain-containing disease resistance protein | TCAATGACAGCAGGGTTT | GCAATGGTTGTTGGAATG |
| miR779 | orange1.1g027903m | AT3G63120.1 | Cyclin p1;1 | CCCTCCTGCTTTGTCATTG | ATTTCTGCTGTGCTTACTCCTC |
|  | orange1.1g044779m | AT2G38290.1 | Ammonium transporter 2 | TCAAGGATGGGCTGCTAT | CGCCTGCTAATTGTTTCA |
|  | orange1.1g041074m | AT2G38290.1 | Ammonium transporter 2 | CTTGCCCGTCACAAACTCA | CATACTTCTCGCCATCACCC |
|  | orange1.1g042791m | AT3G14470.1 | NB-ARC domain-containing disease resistance protein | AGATGGCGAAGGCGAAGA | CACGAGGCAAGCGTTAAATT |
|  | orange1.1g001921m | AT3G45630.1 | RNA binding (RRM/RBD/RNP motifs) family protein | CACCAACTTCTGCCCACC | TCTCCTGCTCCTCCGACA |
|  | orange1.1g025347m | AT2G40610.1 | Expansin A8 | TTCGTGGCACATTTGGAG | TTAGGTGGGCAGAAGTTGG |
|  | orange1.1g045028m | AT1G73660.1 | Protein tyrosine kinase family protein | CAGAGCCCTTCAACAACT | TCTTTAAGCCCGTACCAC |

**Table S3 | List of *known miRNAs* in *Citrus sinensis* leaves.**

| **miRNA** | **Sequence** | | **Expressed** | | **Normalized read count** | | **Fold change** |
| --- | --- | --- | --- | --- | --- | --- | --- |
|  |  |  | **Control** | **Mg-deficiency** | **Control** | **Mg-deficiency** |  |
| ***Up-regulated miRNAs*** | | |  |  |  |  |  |
| miR5832 | UUGGCGGAGCGAUUUGUC | | 0 | 40754 | 0.01 | 2004.8199 | 17.61311311** |
| miR5493 | ACCGGGCUCGAGCGACGCGUG | | 0 | 25261 | 0.01 | 1242.6695 | 16.92308312** |
| miR1104 | CGCAGCUGUUCGUCUUUCCC | | 0 | 4433 | 0.01 | 218.0735 | 14.41252685** |
| miR5760 | UGUUUAAGAUAGUUUGUAAGGAU | | 0 | 3899 | 0.01 | 191.8043 | 14.22734744** |
| miR3443 | GUGCGUUUGAGAUUGAGGUU | | 0 | 2668 | 0.01 | 131.2475 | 13.68000232** |
| miR5666 | AGGGACAUAGAGACAUUUACU | | 0 | 2555 | 0.01 | 125.6886 | 13.61756618** |
| miR7730 | AUGAACACGGCACGAUUGAAGUUAU | | 0 | 1366 | 0.01 | 67.1979 | 12.71420043** |
| miR6166 | AUUAGAUAACGGAUUAAUGGA | | 0 | 1131 | 0.01 | 55.6375 | 12.44184188** |
| miR6233 | UCAAGUUGUUUUGGAAUUACUG | | 0 | 1045 | 0.01 | 51.4069 | 12.3277463** |
| miR4408 | AACAAAUAUGGAUGAUUGUUGGA | | 0 | 1044 | 0.01 | 51.3577 | 12.32636488** |
| miR8039 | UUUCCUACUGAGAUUAUCAAC | | 0 | 956 | 0.01 | 47.0287 | 12.19932574** |
| miR5160 | CAGAAGAUCGAUGGUAAUUUCUU | | 0 | 944 | 0.01 | 46.4384 | 12.18110255** |
| miR1851 | AUAUGGGAUGGCAUUUGGC | | 0 | 840 | 0.01 | 41.3223 | 12.01270484** |
| miR917 | UUUGGACGGUUAUUUUCGAA | | 0 | 814 | 0.01 | 40.0433 | 11.96734516** |
| miR1077 | UUGAAGUGUUCGGAUCGCGGC | | 22 | 74084 | 0.9902 | 3644.4294 | 11.84568538** |
| miR4223 | AUGGUUUUGAUAAAUAGAACAGGC | | 0 | 578 | 0.01 | 28.4337 | 11.47338613** |
| miR1172 | UAGACGGAGACAUAGUGAA | | 0 | 432 | 0.01 | 21.2515 | 11.05334896** |
| miR5821 | UGCGGAGGGAUGGUGGGAG | | 0 | 379 | 0.01 | 18.6442 | 10.86451118** |
| miR1162 | CGGCUUAAUUUGACUCAACACG | | 0 | 359 | 0.01 | 17.6604 | 10.7863023** |
| miR6449 | CUAUGAUUCUGGAAAUAAACGGUU | | 0 | 272 | 0.01 | 13.3806 | 10.38592709** |
| miR395 | GUGAAGUGUUCGGAUCGCC | | 64 | 73994 | 2.8804 | 3640.002 | 10.30345436** |
| miR946 | UGUGGAAUAGAAGGGCUGAGU | | 0 | 255 | 0.01 | 12.5443 | 10.29281625** |
| miR7779 | GAUCAUUGUCGAAAUGCGGAAG | | 0 | 169 | 0.01 | 8.3137 | 9.69934688** |
| miR7727 | CAAGAUCGAUUGGAACGAAUGGA | | 0 | 151 | 0.01 | 7.4282 | 9.53686885** |
| miR5674 | UAAUUGUGUAUGGUACAUUCA | | 0 | 150 | 0.01 | 7.379 | 9.52728151** |
| miR906 | CGGUUGGUGGACGUGAUGG | | 0 | 139 | 0.01 | 6.8379 | 9.41740951** |
| miR1866 | UGAAAUUCCUGUGAAAGAUUGCGU | | 0 | 111 | 0.01 | 5.4604 | 9.09286283** |
| miR5053 | UCGGACUGUAGUCGCCGGCCU | | 0 | 92 | 0.01 | 4.5258 | 8.82202902** |
| miR2610 | AAGAUUUAGACAUUGUAUGGCGUU | | 7 | 2833 | 0.315 | 139.3643 | 8.7892935** |
| miR5491 | UGGAAAUGGGGAUCGUUGGAC | | 0 | 87 | 0.01 | 4.2798 | 8.74139957** |
| miR2585 | AGGAUUAGAGAAAUUACAGAGGAC | | 0 | 84 | 0.01 | 4.1322 | 8.69076627** |
| miR5508 | UAUGAUGGCUGUCGGUGGUGGUGG | | 0 | 83 | 0.01 | 4.083 | 8.67348576** |
| miR5628 | GAGGAAUACGAAGGAUAUGAUUAC | | 0 | 70 | 0.01 | 3.4435 | 8.42773187** |
| miR5774 | GCUUGGCCUACGACACGUGCAU | | 11 | 3200 | 0.4951 | 157.4183 | 8.3126676** |
| miR4383 | UAUUGAAUUCUCAGUUGGACGGU | | 0 | 63 | 0.01 | 3.0992 | 8.27575205** |
| miR5206 | AGUGGUGGUCGUGUUGGUGGGUUC | | 0 | 61 | 0.01 | 3.0008 | 8.22920336** |
| miR3700 | UCGCACCAAAACUGAAGAUCA | | 0 | 59 | 0.01 | 2.9024 | 8.18110255** |
| miR6107 | AAAGCGGACAAUAUCUUGACAGGU | | 0 | 56 | 0.01 | 2.7548 | 8.10580377** |
| miR5540 | UUGUGCGAAUCAACGGUUCUA | | 14 | 3488 | 0.6301 | 171.5859 | 8.08913448** |
| miR400 | AUGUGAGAGUAUUAUAAGUUACA | | 0 | 53 | 0.01 | 2.6072 | 8.02635745** |
| miR3513 | UUGGAUAAGAUAGGAUUGGAU | | 1 | 234 | 0.045 | 11.5112 | 7.99889752** |
| miR5555 | AGGAGAAUAAUAUGCACUUUG | | 0 | 52 | 0.01 | 2.558 | 7.99887245** |
| miR866 | UCAAGGAGACUGAUUUUGUGA | | 0 | 51 | 0.01 | 2.5089 | 7.97091116** |
| miR2905 | UUACAUGACAGGACAAAGCA | | 0 | 50 | 0.01 | 2.4597 | 7.94233856** |
| miR1161 | UGACUUGAGUUCUCCAACUAGC | | 0 | 47 | 0.01 | 2.3121 | 7.85305999** |
| miR812 | ACGGCACGAUUAAAGAUGGGCCAU | | 5 | 1041 | 0.225 | 51.2101 | 7.83035956** |
| miR1087 | AACAGAUUGGUUUUGGCUU | | 0 | 45 | 0.01 | 2.2137 | 7.79031591** |
| miR3438 | UCAAGGAUUGAGAUAGAUAGC | | 13 | 2429 | 0.5851 | 119.4903 | 7.67399457** |
| miR4377 | UAGUCCUCGCUGAUGAAGACG | | 0 | 40 | 0.01 | 1.9677 | 7.62036647** |
| miR5251 | AGUAGAUCAGUUGGAGCUU | | 2 | 329 | 0.09 | 16.1846 | 7.49048099** |
| miR5828 | UUAAGGGCAUUAAAAUUAAAU | | 0 | 36 | 0.01 | 1.771 | 7.4684204** |
| miR1153 | UGUGGCCAUCGUAUGACUUGAG | | 0 | 35 | 0.01 | 1.7218 | 7.42777376** |
| miR6230 | UGACAAGUUUAAGGGCAUUAGA | | 0 | 35 | 0.01 | 1.7218 | 7.42777376** |
| miR1439 | AUUUAGGAACGGGAGUGAGAUAUU | | 0 | 34 | 0.01 | 1.6726 | 7.38594866** |
| miR3628 | AUUGGGAGAGAGUGCUUAGUA | | 0 | 34 | 0.01 | 1.6726 | 7.38594866** |
| miR2612 | UGAUAGUGGCAAUAGUAGAGA | | 0 | 33 | 0.01 | 1.6234 | 7.34287471** |
| miR5205 | AUGACAAUUUGGGACGGGAUUGAG | | 0 | 33 | 0.01 | 1.6234 | 7.34287471** |
| miR2622 | UUUGUGGCCACCGUGAACUAAU | | 0 | 31 | 0.01 | 1.525 | 7.25266543** |
| miR5076 | GAAUGGGAGCGAGAGUAGGUUGAU | | 0 | 30 | 0.01 | 1.4758 | 7.20535341** |
| miR5776 | ACUAUGGGCUGACCUUAGGUGG | | 11 | 1393 | 0.4951 | 68.5261 | 7.11278982** |
| miR2098 | UCUCCGUGGAGGCAUGCAGAU | | 0 | 27 | 0.01 | 1.3282 | 7.05332859** |
| miR6423 | UGCUGUCGCCACUAUCUUCGCAUU | | 0 | 27 | 0.01 | 1.3282 | 7.05332859** |
| miR840 | AGACUGAAGGACCUUAACUCAAGCU | | 0 | 27 | 0.01 | 1.3282 | 7.05332859** |
| miR4350 | UCAAAGUUUUGUGAUCGUUGUGU | | 0 | 26 | 0.01 | 1.279 | 6.99887245** |
| miR5370 | CUCAGAUUCGUCCAAAAAGAA | | 0 | 26 | 0.01 | 1.279 | 6.99887245** |
| miR6228 | GUGGAUAGUAGAAUGAUGAAGGA | | 3 | 349 | 0.135 | 17.1684 | 6.99065238** |
| miR7498 | AUGGAUGACAUGGUAUCUCAA | | 0 | 25 | 0.01 | 1.2298 | 6.9422799** |
| miR7787 | GCUUUAGGUCGUGUGCUUGGGCAC | | 0 | 24 | 0.01 | 1.1806 | 6.88337644** |
| miR6115 | CUGGACGGGUUUGACAUGGCAU | | 0 | 23 | 0.01 | 1.1314 | 6.82196527** |
| miR5286 | ACAAUGGAGGCAAGGGAAGUA | | 30 | 2988 | 1.3502 | 146.9893 | 6.7663942** |
| miR529 | GAGGAAGGAGAGAUGGAGCAG | | 60 | 5700 | 2.7004 | 280.4013 | 6.6981761** |
| miR7780 | GUAUGGGACCUUGGCUAAAGACGU | | 2 | 167 | 0.09 | 8.2153 | 6.51224445** |
| miR4387 | ACUGAGCACUGAUUGGAUGAUGAU | | 4 | 327 | 0.18 | 16.0862 | 6.48168285** |
| miR5181 | CUUUUUGGAUUGAAGGGA | | 4 | 309 | 0.18 | 15.2007 | 6.39999705** |
| miR8019 | AAAAGAAGACCCUUUUGAGCUUG | | 47 | 3599 | 2.1153 | 177.0463 | 6.3871206** |
| miR1168 | UCGUGGACAAGGCCAAGGCGC | | 19 | 1338 | 0.8551 | 65.8205 | 6.26630003** |
| miR7747 | CAUCAAAUGUUUUUGUAGGAUG | | 1 | 70 | 0.045 | 3.4435 | 6.25780687** |
| miR2616 | AUUGGGUUGGGUCGGCCGGU | | 570 | 39850 | 25.6539 | 1960.3492 | 6.25578869** |
| miR6261 | AAGUGUUAGGUAUAGAGAAGCACG | | 7 | 484 | 0.315 | 23.8095 | 6.24004169** |
| miR4366 | CUUGUUGUAGAGUUUGUUGG | | 17 | 1154 | 0.7651 | 56.769 | 6.2133112** |
| miR8127 | CAACUGUGGGAGAUACCUUUA | | 60 | 3359 | 2.7004 | 165.24 | 5.93524603** |
| miR833 | UGAUUUGUUGAUAAUCGGCUCAGU | | 17 | 849 | 0.7651 | 41.765 | 5.7705023** |
| miR7510 | AGAACUGAGACUAUUAGCGGCGU | | 11 | 536 | 0.4951 | 26.3676 | 5.7349025** |
| miR863 | UUGAGAGACAACAAGAAGAUCAU | | 4 | 176 | 0.18 | 8.658 | 5.58796499** |
| miR1440 | UUAAGGAGAGGUUGGCUAUUUGAG | | 6 | 248 | 0.27 | 12.1999 | 5.4977661** |
| miR1069 | UGAUAGAAUCAAAGGUGUCGACUG | | 1 | 40 | 0.045 | 1.9677 | 5.45044147** |
| miR5151 | UAAUUGUGUGGGUAGAAGAA | | 1 | 40 | 0.045 | 1.9677 | 5.45044147** |
| miR6442 | CAGAACGGUUGAAGGACACG | | 5 | 198 | 0.225 | 9.7403 | 5.4359693** |
| miR1160 | UGAAGGAAGCAGUAGUGGAU | | 11 | 413 | 0.4951 | 20.3168 | 5.35880943** |
| miR1512 | UAAGCAGAACAUUCAUGAGCAU | | 28 | 813 | 1.2602 | 39.9941 | 4.98806257** |
| miR6480 | UAUGCUGAAACGACGGAACAU | | 7 | 186 | 0.315 | 9.1499 | 4.86033224** |
| miR5290 | AAUAUGAGUAGAGUAGACACCUA | | 57 | 1386 | 2.5654 | 68.1818 | 4.73213099** |
| miR1441 | CCACAUGUCGGAACAAGGUUU | | 7 | 168 | 0.315 | 8.2645 | 4.71350381** |
| miR5337 | CUAGAACGACGAAGACAUAUUGUGA | | 53 | 1264 | 2.3854 | 62.1802 | 4.70415214** |
| miR7699 | UUUAAUGCAUUGAGACUCA | | 2 | 45 | 0.09 | 2.2137 | 4.62039091** |
| miR7758 | AACCGUUAGUUGACCGUGUAA | | 24 | 533 | 1.0802 | 26.22 | 4.60129733** |
| miR5304 | AUGAUGAGUCUGGUAAUUGGA | | 64 | 1350 | 2.8804 | 66.4108 | 4.5270768** |
| miR418 | UUAAUGUGAUCAUGAAUGAGC | | 8 | 155 | 0.3601 | 7.6249 | 4.40424891** |
| miR7785 | GUGAAUGGGUAGAGAGAGAAGAC | | 22 | 420 | 0.9902 | 20.6611 | 4.38305331** |
| miR1533 | AUAAUAAAAAUAGAUCAUGUG | | 18 | 335 | 0.8101 | 16.4797 | 4.34644616** |
| miR6426 | AGUGGAAGAUGGAAGUGUAGA | | 188 | 3403 | 8.4613 | 167.4045 | 4.30631516** |
| miR6203 | AGACGAUAUCAGAUAGACCUGCAU | | 2 | 35 | 0.09 | 1.7218 | 4.25784876** |
| miR5185 | UUUGAAAAAUGAACUGAAGAA | | 5 | 85 | 0.225 | 4.1814 | 4.21598915** |
| miR5037 | AUGAGAACUUUGAAGGCCGGC | | 1155 | 19027 | 51.9829 | 935.9991 | 4.17039812** |
| miR3520 | AGGUGAUCGGUGAAUAAUUAU | | 79 | 1257 | 3.5555 | 61.8359 | 4.1203203** |
| miR1026 | UGUGAAAUGACUUGAGAGGCC | | 121 | 1867 | 5.4458 | 91.8437 | 4.07596486** |
| miR4404 | ACAUGGAAGACUGGAUGGAUCAA | | 16 | 245 | 0.7201 | 12.0523 | 4.06496741** |
| miR5490 | UAUGGAUUUGUAUUUGGAUGG | | 63 | 921 | 2.8354 | 45.3069 | 3.9981066** |
| miR6286 | UUUAACCAUUGAUCGUCGUUGA | | 13 | 186 | 0.5851 | 9.1499 | 3.96700085** |
| miR1869 | UGGAACAAUGUAGGCAAGGGAAGUA | | 2119 | 28170 | 95.3695 | 1385.7726 | 3.86101877** |
| miR6218 | CGAAAAAUCACGAAGACUUGC | | 30 | 393 | 1.3502 | 19.3329 | 3.83981303** |
| miR8125 | CAGGUAAAAGAAUGGAUGAGU | | 13 | 163 | 0.5851 | 8.0185 | 3.77657726** |
| miR7710 | GUCUACAGAUCGGAUGCAGACUU | | 3 | 36 | 0.135 | 1.771 | 3.7135329** |
| miR4368 | AAGACGGGACUUACUAUCAGUAAA | | 2 | 23 | 0.09 | 1.1314 | 3.65204026** |
| miR5720 | UUGUGAUUUGGGUUGGACAGC | | 4 | 45 | 0.18 | 2.2137 | 3.62039091** |
| miR2916 | UGGGGGCUCGAAGACGAUCAGA | | 6410 | 70604 | 288.4938 | 3473.237 | 3.58966864** |
| miR6281 | AUGGUAGAGAGAGAGAGAGUGAG | | 17 | 185 | 0.7651 | 9.1007 | 3.57225729** |
| miR5536 | AAGGUAGUGACUAUGUACGGUAGU | | 6 | 64 | 0.27 | 3.1484 | 3.54358753** |
| miR847 | AUGUUGAAGAAGAGAAUGGAA | | 9 | 94 | 0.4051 | 4.6242 | 3.51285381** |
| miR5656 | AGUGAGUGAGAGAUUGGGUGU | | 21 | 213 | 0.9451 | 10.4782 | 3.47078011** |
| miR4371 | AUGGUGGUGACGGGUGACGGAGU | | 1823 | 17547 | 82.0475 | 863.1932 | 3.39515222** |
| miR7762 | CUGGAAUAAUGGUCAUAACUGGA | | 4 | 34 | 0.18 | 1.6726 | 3.21602366** |
| miR5999 | CUUCACGAUCAUGACGGACAA | | 75 | 618 | 3.3755 | 30.4014 | 3.17096464** |
| miR5282 | GAGGAAUUAGUAGAAGAUUCAU | | 6 | 49 | 0.27 | 2.4105 | 3.15830112** |
| miR6439 | GAAAUAAGAAGUAGAAGCC | | 11 | 85 | 0.4951 | 4.1814 | 3.07819421** |
| miR1888 | UUGAAGUUAAGAUUUGUGACAA | | 14 | 106 | 0.6301 | 5.2145 | 3.04887621** |
| miR5284 | UAGGGACCUAAGUGGAGAAUCCU | | 6 | 45 | 0.27 | 2.2137 | 3.03542841** |
| miR4379 | AGACUGUAUACUAGGGAAGGCCU | | 17 | 127 | 0.7651 | 6.2475 | 3.02955877** |
| miR3711 | GGCGCUAGAAGGAGGGCAUU | | 109 | 784 | 4.9057 | 38.5675 | 2.97485472** |
| miR5211 | UCGCAGGGGAGAUGGGACCGC | | 20 | 141 | 0.9001 | 6.9362 | 2.9459883** |
| miR443 | AGCACAAACAAUAAAUUGGAC | | 3 | 21 | 0.135 | 1.0331 | 2.9359486** |
| miR7748 | AAUUUCUUCUGAUUGUUGGACUGG | | 3 | 21 | 0.135 | 1.0331 | 2.9359486** |
| miR5750 | AAGAAGAAGAUCAGAAUUCAA | | 9 | 58 | 0.4051 | 2.8532 | 2.81623089** |
| miR5718 | CAGAGACACAAACACAGACACAA | | 21 | 122 | 0.9451 | 6.0016 | 2.66680828** |
| miR5813 | ACAGCAGGACGGUGGUCAUGGA | | 3747 | 21623 | 168.6406 | 1063.7047 | 2.65707388** |
| miR415 | AACAGAGCAGAACAGAACCGU | | 8 | 46 | 0.3601 | 2.2629 | 2.65170333** |
| miR1511 | ACCUGGCUCUGAUACCACAAGGCG | | 5 | 28 | 0.225 | 1.3774 | 2.61395068** |
| miR7837 | UGGAUGGGAGGAUGUGGUGGU | | 49 | 265 | 2.2053 | 13.0362 | 2.56347656** |
| miR7698 | UUUCUCAUCAAAGUUAUUCUGACA | | 4 | 21 | 0.18 | 1.0331 | 2.5209111** |
| miR2105 | UUGAUGAUGUGAAUAAAUUCU | | 12 | 58 | 0.5401 | 2.8532 | 2.40128243** |
| miR2663 | UUGUGAGAGGGCGUAAAUU | | 8 | 38 | 0.3601 | 1.8693 | 2.37602862** |
| miR859 | UGAUUAUUAAAUAGAUCGUA | | 33 | 155 | 1.4852 | 7.6249 | 2.3600612** |
| miR1426 | AGAAUCUUGAUGAUGCAUU | | 9 | 42 | 0.4051 | 2.0661 | 2.35056009** |
| miR8024 | UUAAAAUUUAAAGGACGUCAACUC | | 5 | 23 | 0.225 | 1.1314 | 2.33011217** |
| miR8033 | UUUCAAAGCUGGAGAAAGAU | | 41 | 187 | 1.8453 | 9.1991 | 2.31763734** |
| miR7711 | UGAUGGAAUUGUAAAUUAGAAGA | | 9 | 40 | 0.4051 | 1.9677 | 2.28016029** |
| miR533 | GAGUGGCCAGGGCUGUUGGAGGGC | | 20 | 87 | 0.9001 | 4.2798 | 2.24938618** |
| miR5378 | CAUUGAAGGAAUAGAAGACACA | | 19 | 79 | 0.8551 | 3.8863 | 2.18423222** |
| miR7485 | AAAGACCAUCUUUGAUUCGUUUGA | | 98 | 380 | 4.4107 | 18.6934 | 2.08344945** |
| miR6180 | AGGAGGAGAGAAAGACGGGCG | | 8 | 30 | 0.3601 | 1.4758 | 2.03502772** |
| miR1507 | UCUUCAUUCCACACGUCGUCU | | 70 | 255 | 3.1505 | 12.5443 | 1.99337925** |
| miR835 | AAUGAGAAGAUGGACGCAAGAAAG | | 30 | 105 | 1.3502 | 5.1653 | 1.93567902** |
| miR4414 | AGCUGCUGACUCGUUGGUUC | | 99 | 346 | 4.4557 | 17.0208 | 1.93357484** |
| miR319 | AUCCAACGAAGCAGGAGCUGU | | 9 | 31 | 0.4051 | 1.525 | 1.91245925** |
| miR5292 | AUUCAGUAGAGCAACAAAGAAGGC | | 31 | 102 | 1.3952 | 5.0177 | 1.84655427** |
| miR7764 | CAAAACCUUAGAUCUGGAUCAA | | 59 | 194 | 2.6554 | 9.5435 | 1.84558926** |
| miR5485 | UGACAAGUGGUAUCAGAGCAA | | 152 | 496 | 6.841 | 24.3998 | 1.83459019** |
| miR846 | UUGAAUUGAGUCGCUUGAAGA | | 15 | 48 | 0.6751 | 2.3613 | 1.80640822** |
| miR5807 | AGGACUCUAGGAGGUGAUGUGGC | | 319 | 1000 | 14.3572 | 49.1932 | 1.77668449** |
| miR5029 | GAGAGAGAGAACACGUGCACAA | | 13 | 40 | 0.5851 | 1.9677 | 1.74975516** |
| miR5218 | GUGAGACUUGGAGUACAAAAUGAU | | 16 | 48 | 0.7201 | 2.3613 | 1.71331217** |
| miR7716 | UCUGUUCUAGAGAAAACGACUAAC | | 8 | 23 | 0.3601 | 1.1314 | 1.65163957** |
| miR5793 | CGAGGACGGACACGAGAGCAG | | 10 | 28 | 0.4501 | 1.3774 | 1.61363011** |
| miR398 | GGGGCGACAUGAGAUCACAUG | | 8029 | 22159 | 361.3598 | 1090.0722 | 1.59291577** |
| miR951 | UUGUACUUGACGUGUGGACAC | | 28 | 72 | 1.2602 | 3.5419 | 1.49087077** |
| miR4349 | AUUAGGCUUAGAGAGAAGACAAAG | | 56 | 142 | 2.5204 | 6.9854 | 1.47069002** |
| miR1310 | AGAGGCAUCGGGGGCGCAACGC | | 945 | 2373 | 42.5315 | 116.7355 | 1.45663972** |
| miR6229 | AUUCUAACGGGGACGGAAAGGG | | 50 | 124 | 2.2503 | 6.1 | 1.43869189** |
| miR5246 | UCUCCAGAGAGUUUGAAGGUU | | 36 | 89 | 1.6202 | 4.3782 | 1.43416595** |
| miR7532 | UGAACAGCCUCUGGUCGAUGGU | | 3609 | 8670 | 162.4296 | 426.5051 | 1.39274843** |
| miR5298 | UUGGAGAUGAUAACGAAGAUGAC | | 598 | 1379 | 26.9141 | 67.8374 | 1.33371869** |
| miR3441 | CGAGAAAAUGUACUCUUUU | | 10 | 23 | 0.4501 | 1.1314 | 1.32979161* |
| miR4376 | ACGCAGGAAAGAGAAGACUGGA | | 12 | 27 | 0.5401 | 1.3282 | 1.29817395** |
| miR5262 | UCUGAAUCAGUAGACUCAUUU | | 190 | 427 | 8.5513 | 21.0055 | 1.29655146** |
| miR3631 | AUAUUGGUUGAUCUCAUACAA | | 13 | 29 | 0.5851 | 1.4266 | 1.28582576** |
| miR5024 | AUAAGAACGGCCAAGAUACUAACA | | 202 | 447 | 9.0914 | 21.9894 | 1.27423386** |
| miR5751 | UUGAAUUUGAUCGUUGGUGUU | | 10 | 22 | 0.4501 | 1.0823 | 1.26578298* |
| miR5224 | CGGAAGACAUUGUCAGGAC | | 10604 | 22940 | 477.2524 | 1128.4921 | 1.24157196** |
| miR6190 | GAGGAAAGGAAGAAGCAGCUG | | 23 | 49 | 1.0352 | 2.4105 | 1.21942291** |
| miR1535 | CUUCUUUGUGGUAGAUUGUUU | | 670 | 1399 | 30.1546 | 68.8213 | 1.19047705** |
| miR2938 | UGAUCUUCUGAGAAGGGUUCGAG | | 216 | 433 | 9.7215 | 21.3007 | 1.13165** |
| miR5208 | ACAUGGAUGUAGGUGGGUUUGUUA | | 17 | 33 | 0.7651 | 1.6234 | 1.08529829* |
|  |  | |  |  |  |  |  |
| ***Down-regulated miRNAs*** | | |  |  |  |  |  |
| miR4351 | | AUUGGGAGUGUGCAGUGGGAGUGG | 5736 | 0 | 258.1592 | 0.01 | -14.65597294** |
| miR5073 | | GAUUGGUGAACGGCAGAAGUAUUU | 2978 | 0 | 134.0303 | 0.01 | -13.71027147** |
| miR1135 | | UUCGCUCAAGUAAUUCUGACGGA | 2059 | 0 | 92.6691 | 0.01 | -13.17787312** |
| miR1516 | | AAAGUUGGGACUUGAGGAGGCGGU | 709 | 0 | 31.9098 | 0.01 | -11.63978408** |
| miR5574 | | UUUAAAUAGAACUCUAAAAGA | 657 | 0 | 29.5695 | 0.01 | -11.52989416** |
| miR4397 | | UGUCUAAGAGUGUGGCGACAUUAC | 464 | 0 | 20.8832 | 0.01 | -11.02812713** |
| miR5525 | | UCAAUCCUUGUGGAGACGAUCUGA | 418 | 0 | 18.8129 | 0.01 | -10.87750643** |
| miR6162 | | UUUGAUACGACUGUCAUUUACGG | 395 | 0 | 17.7777 | 0.01 | -10.79585287** |
| miR6138 | | CACGUUUGGGAUUGAGGUUGAAA | 372 | 0 | 16.7425 | 0.01 | -10.70929917** |
| miR6108 | | UAUGGGUGAGAAGGGAAGAUA | 33936 | 19 | 1527.3518 | 0.9347 | -10.67424131** |
| miR5025 | | AUCUGUAUAUAUGAGUAAUGAUCA | 337 | 0 | 15.1673 | 0.01 | -10.56674863** |
| miR4381 | | UUGUGACGGUCAACUGGUGAAAU | 334 | 0 | 15.0323 | 0.01 | -10.55385004** |
| miR7130 | | GUUUGGAAGUGUGGUGAGGUGGC | 328 | 0 | 14.7622 | 0.01 | -10.52769204** |
| miR7697 | | UCCGGAUAAAUUAUGAUCGGAUA | 299 | 0 | 13.457 | 0.01 | -10.39414106** |
| miR6032 | | UGGAGCAUGAAUCAGAAUCGG | 296 | 0 | 13.322 | 0.01 | -10.37959505** |
| miR1165 | | UACUGUAGCAAGCGGCCAUCC | 288 | 0 | 12.962 | 0.01 | -10.34007259** |
| miR3509 | | AUCUAACGACUGCUACAUAAUCAU | 278 | 0 | 12.5119 | 0.01 | -10.28908522** |
| miR5782 | | UAGCUGUGUAGUAGAAGUUGAGA | 278 | 0 | 12.5119 | 0.01 | -10.28908522** |
| miR5074 | | GCAAGGCCACCGUGCCGGCGACGC | 271 | 0 | 12.1969 | 0.01 | -10.25229873** |
| miR5516 | | CUGCAGUUGCUGUCGGGUAGGCGG | 268 | 0 | 12.0618 | 0.01 | -10.23622943** |
| miR782 | | ACAAACAGAGUUGGAUUUCUU | 249 | 0 | 11.2067 | 0.01 | -10.13014587** |
| miR5023 | | UUGGUAGUGAUAAAGGCGC | 239 | 0 | 10.7566 | 0.01 | -10.0710064** |
| miR7708 | | UGUCAUGAACUGAACGAAAGACAGC | 239 | 0 | 10.7566 | 0.01 | -10.0710064** |
| miR7545 | | UAGGAAAGUAGAGUGCAU | 205 | 0 | 9.2264 | 0.01 | -9.84962397** |
| miR5780 | | AAACUUAACUGACGGUAGGGA | 2925 | 3 | 131.645 | 0.1476 | -9.80074424** |
| miR2597 | | UUUGGUAGCUUCGAGAUGUGA | 185 | 0 | 8.3263 | 0.01 | -9.70153168** |
| miR5163 | | UAGAAUAAUUCAGGUGUGUCGGAU | 185 | 0 | 8.3263 | 0.01 | -9.70153168** |
| miR5781 | | CGAAACUUGAGAAUGCAUCUGC | 176 | 0 | 7.9212 | 0.01 | -9.62957518** |
| miR6277 | | UGUUGUGGGAGAGCGAUAC | 170 | 0 | 7.6512 | 0.01 | -9.57954225** |
| miR2863 | | AUAUAGGGACUAAAUGGGCAAA | 836 | 1 | 37.6257 | 0.0492 | -9.57884436** |
| miR6247 | | UGGCUGAAUGAACAUAAGGCA | 829 | 1 | 37.3107 | 0.0492 | -9.56671537** |
| miR7763 | | AAUUUAAUGUCGGGGAUG | 168 | 0 | 7.5611 | 0.01 | -9.56245231** |
| miR5230 | | CAAAGUCUUGAAUUGUUGGAA | 159 | 0 | 7.1561 | 0.01 | -9.48302974** |
| miR5759 | | AACGGGGUGAAGAAGAUUAGA | 155 | 0 | 6.9761 | 0.01 | -9.4462769** |
| miR848 | | GACGUGGGACUAACAGAGGCUAUA | 153 | 0 | 6.886 | 0.01 | -9.42752237** |
| miR5633 | | UUGAACAUCAGAAAUCAGUGC | 152 | 0 | 6.841 | 0.01 | -9.41806341** |
| miR2864 | | UGGUUUUGAUUGUAUGGUC | 148 | 0 | 6.661 | 0.01 | -9.37959496** |
| miR384 | | GGGGGCAAAGUGGCAAUC | 135 | 0 | 6.0759 | 0.01 | -9.24695432** |
| miR868 | | UCAUGUAAGAAAUAUAGUCAC | 135 | 0 | 6.0759 | 0.01 | -9.24695432** |
| miR1870 | | UGAAAUUAGGACCUAGUGGCAU | 130 | 0 | 5.8509 | 0.01 | -9.19251472** |
| miR3933 | | AGAAGAAAAUGAAGAACACGG | 125 | 0 | 5.6259 | 0.01 | -9.13594008** |
| miR948 | | UGUGGUCGUGGGUUCGGG | 121 | 0 | 5.4458 | 0.01 | -9.08900019** |
| miR8014 | | AGUGAAUAGAAAGUUGGAUAAAU | 104 | 0 | 4.6807 | 0.01 | -8.87058048** |
| miR5041 | | UUCUCUUUCAACUUGCUCGAA | 102 | 0 | 4.5907 | 0.01 | -8.84257037** |
| miR5826 | | UAGGGAAAGUAGAAGAGUGGAGU | 102 | 0 | 4.5907 | 0.01 | -8.84257037** |
| miR1024 | | UUGGUUGGAUUUGGCAUC | 101 | 0 | 4.5457 | 0.01 | -8.82835867** |
| miR6461 | | UAGCUGGAGUUGAUGGAUC | 100 | 0 | 4.5007 | 0.01 | -8.81400559** |
| miR5512 | | UAGGAUAAGGAAAUGGCUAACAA | 97 | 0 | 4.3657 | 0.01 | -8.77006919** |
| miR6192 | | UGAGGAGAGGUGGGAAUGGGAAUC | 91 | 0 | 4.0956 | 0.01 | -8.67793101** |
| miR8000 | | AACCGAAAAACCGAACCGAAGAA | 86 | 0 | 3.8706 | 0.01 | -8.5964134** |
| miR5543 | | UAUGAGUGGCUAAUUUUCUUU | 85 | 0 | 3.8256 | 0.01 | -8.5795422** |
| miR8129 | | AAAUAAAUGUACGGAUGUCA | 78 | 0 | 3.5105 | 0.01 | -8.4555327** |
| miR444 | | UGCAUGUUAGUUGUGGCAAGCUU | 759 | 2 | 34.1602 | 0.0984 | -8.43944239** |
| miR1046 | | UGUUUUCAUAUUUUUCAU | 75 | 0 | 3.3755 | 0.01 | -8.39895739** |
| miR7842 | | UAAUCAAGCCGGGUACUUCUA | 74 | 0 | 3.3305 | 0.01 | -8.37959496** |
| miR6194 | | UAGGGGAUCGAAGACGAU | 73 | 0 | 3.2855 | 0.01 | -8.35996911** |
| miR6200 | | UUUGUCCAAGCUAGAUCUAA | 68 | 0 | 3.0605 | 0.01 | -8.25762354** |
| miR2095 | | UGAUCAUUUUACGAUGAACUAA | 67 | 0 | 3.0155 | 0.01 | -8.23625344** |
| miR8134 | | UGAGAAUGUAGCAGAGGAUUU | 66 | 0 | 2.9705 | 0.01 | -8.21456199** |
| miR5278 | | AAAAUAUGCAUGCAGAAAUGUGAA | 64 | 0 | 2.8804 | 0.01 | -8.17012538** |
| miR7123 | | AUAAGAGGGGAUGUGGUAAAAAGG | 63 | 0 | 2.8354 | 0.01 | -8.14740848** |
| miR5495 | | GAGGUCCGGAUACGAAGAG | 62 | 0 | 2.7904 | 0.01 | -8.12432813** |
| miR779 | | UCUGCUCAUAGAUUGUCUGCUCAU | 603 | 2 | 27.1391 | 0.0984 | -8.10749886** |
| miR7514 | | AUAAACUGUAAGUGAGAGUCU | 59 | 0 | 2.6554 | 0.01 | -8.05278538** |
| miR2677 | | AUUUAUUGAUGAUUGUAACAGAAU | 58 | 0 | 2.6104 | 0.01 | -8.02812709** |
| miR1105 | | UUCGGUAUCUGAAGAUCGCAUUGC | 56 | 0 | 2.5204 | 0.01 | -7.9775089** |
| miR6110 | | AUCUUGCAUUUGAUGAUUGUGGA | 56 | 0 | 2.5204 | 0.01 | -7.9775089** |
| miR851 | | UCUGGGUGGAAACAAGCGACAG | 56 | 0 | 2.5204 | 0.01 | -7.9775089** |
| miR2620 | | UUCUGAUGAUAACCGGCUUGCUGC | 53 | 0 | 2.3854 | 0.01 | -7.89808741** |
| miR5143 | | GUGGAUGAUAGCAAUGUAGGAAG | 52 | 0 | 2.3404 | 0.01 | -7.87061131** |
| miR5285 | | UAGGACUUAGGGCUAAAUUAGGC | 50 | 0 | 2.2503 | 0.01 | -7.81397355** |
| miR7772 | | GAGACUAAUGAAUGAGAAACGG | 50 | 0 | 2.2503 | 0.01 | -7.81397355** |
| miR5500 | | AUUACUUGAAGAAAAUCUUGCGGC | 47 | 0 | 2.1153 | 0.01 | -7.72471848** |
| miR5561 | | CAUAAGAGAGAGAGCAUAGACAA | 457 | 2 | 20.5681 | 0.0984 | -7.70753451** |
| miR2608 | | UUGUGACUAUAUCAUUACUCU | 46 | 0 | 2.0703 | 0.01 | -7.69369604** |
| miR5554 | | UGUCCUCUUGAACGAUGGUU | 222 | 1 | 9.9915 | 0.0492 | -7.66589914** |
| miR7724 | | CUGAACACUGGACUGCGGCCAACC | 45 | 0 | 2.0253 | 0.01 | -7.66199182** |
| miR4233 | | AAUCAUAUCAACAUAACUCC | 44 | 0 | 1.9803 | 0.01 | -7.6295752** |
| miR5259 | | CAAGGGGUAUUUGGAUGGACA | 617 | 3 | 27.7692 | 0.1476 | -7.5556491** |
| miR853 | | CAACCGGUAGCAAGAGGAGCAGCA | 41 | 0 | 1.8453 | 0.01 | -7.52771158** |
| miR5501 | | CUUAUGGCAGAACGGGUGAG | 38 | 0 | 1.7103 | 0.01 | -7.41810561** |
| miR2661 | | UAUGGUUUGGAAGAAAAUGUGGCA | 37 | 0 | 1.6653 | 0.01 | -7.3796383** |
| miR5513 | | UAACAAAGGCACACGAACUGA | 36 | 0 | 1.6202 | 0.01 | -7.34002811** |
| miR2667 | | CCUGUGAUCUGAGGCAACC | 35 | 0 | 1.5752 | 0.01 | -7.29939121** |
| miR6145 | | CAUUGUUCACAUGUACGGCACUAU | 516 | 3 | 23.2235 | 0.1476 | -7.2977489** |
| miR6198 | | GCUGUUCUUAGAGAUGGUCGAUUC | 34 | 0 | 1.5302 | 0.01 | -7.25757641** |
| miR8005 | | UUUAGAGUUUUACUGUUUAGGUAUU | 34 | 0 | 1.5302 | 0.01 | -7.25757641** |
| miR5752 | | CAUUGAUUGGUUUGGUCAA | 32 | 0 | 1.4402 | 0.01 | -7.17012536** |
| miR5214 | | UGAUAGAGCGGAGGACCACGGCAG | 31 | 0 | 1.3952 | 0.01 | -7.12432813** |
| miR7812 | | AUGUUAGGAAAUUGAUGGGUG | 887 | 6 | 39.9211 | 0.2952 | -7.07931495** |
| miR163 | | UUGAACAGGCUUUGAGACUUCGA | 30 | 0 | 1.3502 | 0.01 | -7.07702931** |
| miR4237 | | AGACAGCGUAAACAUAUAAAUUGA | 29 | 0 | 1.3052 | 0.01 | -7.02812709** |
| miR5381 | | AAGACUGUGGCAGCCAAGC | 29 | 0 | 1.3052 | 0.01 | -7.02812709** |
| miR6475 | | UCUUGCGAGAGUAAAGAAUGA | 3233 | 23 | 145.5071 | 1.1314 | -7.00683666** |
| miR2912 | | UCUAGAAAUCGUGUUGGGC | 28 | 0 | 1.2602 | 0.01 | -6.9775089** |
| miR5488 | | UGAAUGGACUGAUGAUUGCCA | 27 | 0 | 1.2152 | 0.01 | -6.92504996** |
| miR6209 | | UGAGAUCAGAAUGUAUGGGCGG | 27 | 0 | 1.2152 | 0.01 | -6.92504996** |
| miR7739 | | AGUGCGCGAACGGACUGGCAU | 27 | 0 | 1.2152 | 0.01 | -6.92504996** |
| miR1109 | | UAGUGGGAGAUUGUUGGGAUAAU | 26 | 0 | 1.1702 | 0.01 | -6.87061131** |
| miR7731 | | GGUUAGCACUCUGGACUUUGAAUC | 26 | 0 | 1.1702 | 0.01 | -6.87061131** |
| miR7813 | | UGGUAAUGAAGAUGAUGCUGA | 26 | 0 | 1.1702 | 0.01 | -6.87061131** |
| miR852 | | AGAACAAAGAGCGGCCUUACCUAU | 26 | 0 | 1.1702 | 0.01 | -6.87061131** |
| miR5171 | | ACUUAUAUGGGACGGGCAGA | 25 | 0 | 1.1252 | 0.01 | -6.81403765** |
| miR5248 | | UUUUUAGGUUGAGCAUGACAUACA | 25 | 0 | 1.1252 | 0.01 | -6.81403765** |
| miR1509 | | UUAUUCAAGGAUCACGGAU | 24 | 0 | 1.0802 | 0.01 | -6.75515464** |
| miR5067 | | CAUGCGACAACUAAUGAUGCAU | 24 | 0 | 1.0802 | 0.01 | -6.75515464** |
| miR5798 | | UGGACUACAAGAUUCAGAGAU | 24 | 0 | 1.0802 | 0.01 | -6.75515464** |
| miR6248 | | AUGUUGUAGGAAUGGAGGUAGGUA | 990 | 9 | 44.5568 | 0.4427 | -6.65317244** |
| miR1114 | | AAGAGAGGACAAAACAUUGGCAUG | 211 | 2 | 9.4964 | 0.0984 | -6.59257858** |
| miR1044 | | UUGAGGGCAUAUUUCUUUUU | 98 | 1 | 4.4107 | 0.0492 | -6.48620551** |
| miR869 | | UUUGGUUCAGUUCGGGUUG | 179 | 2 | 8.0562 | 0.0984 | -6.35529737** |
| miR6282 | | GUUGAUCGAUAGUGGGAGUACCCA | 89 | 1 | 4.0056 | 0.0492 | -6.34721623** |
| miR822 | | UGCGAGGAGGCAUUUGGACAG | 88 | 1 | 3.9606 | 0.0492 | -6.33091688** |
| miR5819 | | AGGCGAUGGGAACGGUGGCUG | 85 | 1 | 3.8256 | 0.0492 | -6.28088391** |
| miR5373 | | UGUCUUGAUUUUAGAUGCAUG | 3543 | 47 | 159.4592 | 2.3121 | -6.10783973** |
| miR158 | | UUCAAAACUGUUAGACAAAGC | 364 | 5 | 16.3825 | 0.246 | -6.05735341** |
| miR5789 | | UGAUGAGCAUCUGGUCGGUAU | 289 | 4 | 13.007 | 0.1968 | -6.04641422** |
| miR5158 | | UGAGACGGGAUGAGAUGAGAU | 465 | 7 | 20.9282 | 0.3444 | -5.92521928** |
| miR6427 | | GUGGAGAAUGAAAUUAUGAAGA | 64 | 1 | 2.8804 | 0.0492 | -5.87146704** |
| miR6425 | | UUGCUUCCGUGGACAUAGGCA | 308 | 5 | 13.8621 | 0.246 | -5.81634371** |
| miR773 | | UAUCUUUGAACAGAUUUGA | 116 | 2 | 5.2208 | 0.0984 | -5.72946877** |
| miR5721 | | AGAAAAUGGUAGAGAGAAAGUGGA | 346 | 6 | 15.5724 | 0.2952 | -5.72115477** |
| miR1879 | | GUUUGUUUGGUUUGGGAGAGAUGG | 114 | 2 | 5.1308 | 0.0984 | -5.70438166** |
| miR5217 | | AAUCUAUUUUGAAUCGGAUCGGAU | 56 | 1 | 2.5204 | 0.0492 | -5.67885059** |
| miR1149 | | CGGAUCAAUACUGACCCCCAGC | 392 | 7 | 17.6427 | 0.3444 | -5.67884241** |
| miR7712 | | UUCAAUUGUAGAAACUUUAGAUGGC | 333 | 7 | 14.9873 | 0.3444 | -5.44351155** |
| miR5026 | | AUUCAAUAAGAUCGUGACACA | 47 | 1 | 2.1153 | 0.0492 | -5.42606016** |
| miR5031 | | UUGAAUUAAAAUCUAAUUU | 47 | 1 | 2.1153 | 0.0492 | -5.42606016** |
| miR5247 | | GCAGAAGCAGAACUGAUGA | 145 | 4 | 6.526 | 0.1968 | -5.05139686** |
| miR6186 | | CGAGGAACGGCUGAGGGAG | 34 | 1 | 1.5302 | 0.0492 | -4.9589181** |
| miR6290 | | UGAAUGAGGUAGAGAUUCAUGGUA | 34 | 1 | 1.5302 | 0.0492 | -4.9589181** |
| miR5818 | | UCGAACUGAGAGGCACAGGUU | 507 | 15 | 22.8185 | 0.7379 | -4.95063483** |
| miR5268 | | ACAAGUGGAAUGAGAUGGAUGGUU | 337 | 10 | 15.1673 | 0.4919 | -4.94645542** |
| miR5578 | | UUAUGUGGAUGAUGGCAAGAAGGU | 32 | 1 | 1.4402 | 0.0492 | -4.87146704** |
| miR1886 | | UCGGAAGAGAAGUGAAGAUGAAAU | 125 | 4 | 5.6259 | 0.1968 | -4.83728178** |
| miR4359 | | AGCGAAUGACUCUAAUCACUCGGU | 85 | 3 | 3.8256 | 0.1476 | -4.69592141** |
| miR7546 | | UUGGUGCGACAGGCUGCCUGC | 55 | 2 | 2.4754 | 0.0984 | -4.65285954** |
| miR7752 | | AAAAUGAUAGGUCGUGGAAAACAG | 55 | 2 | 2.4754 | 0.0984 | -4.65285954** |
| miR5226 | | UUUGAAACUUGGAGAUGCA | 135 | 5 | 6.0759 | 0.246 | -4.6263679** |
| miR5068 | | UCGGGUUAGAUCGGGUUGGGUAAU | 78 | 3 | 3.5105 | 0.1476 | -4.5719119** |
| miR7767 | | CCCAAGAUGAGUGCUCUCC | 322 | 14 | 14.4922 | 0.6887 | -4.39525714** |
| miR5175 | | AAAAAUUUAGGAACAGGAGUGGGA | 23 | 1 | 1.0352 | 0.0492 | -4.3951074** |
| miR3437 | | AAAAAUACAAGGACUAAACGGAU | 892 | 39 | 40.1461 | 1.9185 | -4.38720917** |
| miR5260 | | UUUGAAUGUUGACAAUGGCUG | 135 | 6 | 6.0759 | 0.2952 | -4.3633335** |
| miR5556 | | UGAUGAGGAAGAACUGCAGAA | 43 | 2 | 1.9353 | 0.0984 | -4.2977551** |
| miR6443 | | AAUGACAUAGAUGAUGGAU | 222 | 11 | 9.9915 | 0.5411 | -4.20673414** |
| miR5830 | | AUGAGAGGAGGUGAUGUGACAUCA | 239 | 12 | 10.7566 | 0.5903 | -4.18762998** |
| miR5369 | | UGAGAAAGGAGAGAUGGUGCA | 16576 | 868 | 746.0332 | 42.6997 | -4.126942** |
| miR5519 | | UGGUAGACGCUACGGACUUAG | 1606 | 90 | 72.281 | 4.4274 | -4.02908484** |
| miR7996 | | AUGUGGAGAAUGAAAUUAUGAAGA | 35 | 2 | 1.5752 | 0.0984 | -4.00073289** |
| miR3522 | | CGACCAAAGGAAGCAGCUGAU | 34 | 2 | 1.5302 | 0.0984 | -3.9589181** |
| miR6279 | | UAAGACAAAAUUACAGAGACU | 34 | 2 | 1.5302 | 0.0984 | -3.9589181** |
| miR5301 | | UGGGGUGGGGAUGGGAAAAGCAUU | 197 | 12 | 8.8663 | 0.5903 | -3.90881193** |
| miR8143 | | GGGAUGGUGGAAAGAUGAUGGUA | 423 | 26 | 19.0379 | 1.279 | -3.89578618** |
| miR7497 | | ACAUGUGACUGUAGCAUAGGCUU | 32 | 2 | 1.4402 | 0.0984 | -3.87146705** |
| miR7499 | | UAUAAAUUUGGGUUGAAUUCGGU | 124 | 8 | 5.5808 | 0.3935 | -3.8260364** |
| miR5630 | | GCGGAAGAGCGGUUCUGAUGC | 62 | 4 | 2.7904 | 0.1968 | -3.82566982** |
| miR6173 | | CUAGCCGUAAACGAUGGAUACUAG | 106 | 7 | 4.7707 | 0.3444 | -3.79204392** |
| miR7539 | | UAGAGAGAGAGAAGCACGAGA | 200 | 14 | 9.0014 | 0.6887 | -3.70820182** |
| miR8145 | | AAAAAGAUAGAAGAGCAGCCU | 51 | 4 | 2.2953 | 0.1968 | -3.5438806** |
| miR2093 | | UGGAUUAAUUGGCAGAAUCA | 63 | 5 | 2.8354 | 0.246 | -3.52682205** |
| miR5648 | | AUUGAAGAACAAGGAGCGGCAU | 49 | 4 | 2.2053 | 0.1968 | -3.4861728** |
| miR5712 | | AUUAUUAAUAUAAUUGAGUGGAGA | 24 | 2 | 1.0802 | 0.0984 | -3.45649633** |
| miR838 | | UGAAGAAUAAGAGGAGAGCAAAGC | 24 | 2 | 1.0802 | 0.0984 | -3.45649633** |
| miR8002 | | UUUUCGUCGAUACAUAAUGCAAGCA | 23 | 2 | 1.0352 | 0.0984 | -3.3951074** |
| miR6151 | | CUGAUAUGUAGAGGAUUGGAUUGU | 45 | 4 | 2.0253 | 0.1968 | -3.3633335** |
| miR5570 | | AACAAGACAAAUCUGAUGACA | 44 | 4 | 1.9803 | 0.1968 | -3.33091688** |
| miR2670 | | UCUCAACUCGAUCGGAUCAUA | 33 | 3 | 1.4852 | 0.1476 | -3.33089259** |
| miR5256 | | AUAUAGGAUUAUGAAGAUUAA | 271 | 27 | 12.1969 | 1.3282 | -3.19897021** |
| miR1097 | | UGCCUGUUGUUGAUUGGAA | 69 | 7 | 3.1055 | 0.3444 | -3.17266852** |
| miR5263 | | AGGCUAAAACAGAACGGGG | 186 | 19 | 8.3713 | 0.9347 | -3.16287638** |
| miR3981 | | AGAAAUAGGAUCGUCUCAU | 27 | 3 | 1.2152 | 0.1476 | -3.04142915** |
| miR5210 | | UAGGAUGUGUUUGGAAUUGAGGUU | 377 | 42 | 16.9676 | 2.0661 | -3.03780053** |
| miR2600 | | AUAGCAAUUCUGGCUGUGAUUGGU | 59 | 7 | 2.6554 | 0.3444 | -2.94677215** |
| miR1222 | | UUAAGAGAGGUCAGUGGUUCA | 421 | 52 | 18.9479 | 2.558 | -2.88894979** |
| miR5742 | | CCAAAUCAGAUGGUCGCGGAU | 453 | 56 | 20.3881 | 2.7548 | -2.88770785** |
| miR1115 | | UGAUAUCGGCAUUUGGUGG | 24 | 3 | 1.0802 | 0.1476 | -2.87153383** |
| miR8041 | | AUGCUUUGCGUAUUGCUCAUUG | 56 | 7 | 2.5204 | 0.3444 | -2.87149567** |
| miR5056 | | AGGAAGAACGGAUAAGCAC | 70 | 9 | 3.1505 | 0.4427 | -2.83117953** |
| miR8050 | | AAGAAGGAAUCAAAGGUAU | 113 | 15 | 5.0858 | 0.7379 | -2.78497751** |
| miR5665 | | UUGGUGGAAAAUGAUUUGGGA | 27 | 4 | 1.2152 | 0.1968 | -2.62639165** |
| miR5072 | | UUCGUUCCCCAGUGGAGUCGCCA | 78 | 12 | 3.5105 | 0.5903 | -2.57215628** |
| miR164 | | UGGAGAAGCAGGGCACGUGCA | 57341 | 9234 | 2580.7366 | 454.25 | -2.50622448** |
| miR8030 | | UAUCGGGUUGGGUUUGGUUCGGUU | 49 | 8 | 2.2053 | 0.3935 | -2.48653939** |
| miR5176 | | UUGAUGAUGCUGCAUAGAAUC | 35 | 6 | 1.5752 | 0.2952 | -2.41577039** |
| miR1852 | | AUAUGUGGAUUCGAAUGCAGGUAA | 23 | 4 | 1.0352 | 0.1968 | -2.3951074** |
| miR5368 | | AGGGACAGUCUCAGGUAGA | 589 | 103 | 26.509 | 5.0669 | -2.38730699** |
| miR1536 | | AAGCGGAGACAAAGUGUAGUGUA | 36 | 7 | 1.6202 | 0.3444 | -2.23401486** |
| miR2913 | | GAGGCUCGUGGAUGCAAGGA | 56 | 11 | 2.5204 | 0.5411 | -2.21968557** |
| miR3629 | | GAGCUCUGGAAAAUGUAGAA | 35 | 7 | 1.5752 | 0.3444 | -2.19337797** |
| miR3946 | | GUGUAGAGAGAGAGAGAGAGACAC | 235 | 48 | 10.5766 | 2.3613 | -2.16322268** |
| miR5653 | | CUGGGUUGGGUAGAGUUGGUUGC | 51 | 11 | 2.2953 | 0.5411 | -2.08471558** |
| miR4995 | | UAGGCAGUGGCUUGGUUAAGGGA | 95 | 21 | 4.2756 | 1.0331 | -2.04914698** |
| miR4250 | | UCACCAAGGAGAAGAACAUCA | 36 | 8 | 1.6202 | 0.3935 | -2.04173637** |
| miR4249 | | UGAAUGUUGAGAAGUGUGAUGGUC | 31 | 7 | 1.3952 | 0.3444 | -2.0183149** |
| miR173 | | GAUUCUCUGGGCGAAGUGGAAUGC | 70 | 16 | 3.1505 | 0.7871 | -2.00096196** |
| miR5077 | | UUCACGUCGGGUUCACCA | 5365 | 1293 | 241.4616 | 63.6068 | -1.92454086** |
| miR156 | | UUGACAGAAGAGAGUGAGCAC | 475712 | 115344 | 21410.289 | 5674.141 | -1.91583036** |
| miR1216 | | UGUAGUGAUGGCGGUUGUAUC | 28 | 7 | 1.2602 | 0.3444 | -1.87149567** |
| miR5758 | | UGAACUUGGAGAAUGUAUUUG | 59 | 15 | 2.6554 | 0.7379 | -1.84743198** |
| miR2111 | | UAAUCUGCAUCCUGAGGUUUG | 118 | 30 | 5.3108 | 1.4758 | -1.84743198** |
| miR5667 | | AAAAGAACUCAAAUGGAUUGC | 47 | 12 | 2.1153 | 0.5903 | -1.84134204** |
| miR7728 | | UUGGAUUUGAGUGUAUUUU | 70 | 18 | 3.1505 | 0.8855 | -1.8310166** |
| miR4992 | | AUGCUGAAGAUGGUUUUGUUGAGU | 23 | 6 | 1.0352 | 0.2952 | -1.8101449** |
| miR4352 | | UAAUAAGUAGACAUUUAAUGACGG | 41 | 11 | 1.8453 | 0.5411 | -1.76988824** |
| miR6476 | | UCAGUAGAGAUGAAAUCAGAU | 92 | 25 | 4.1406 | 1.2298 | -1.75141613** |
| miR2118 | | GUCGAUGGAACAAUGUAGGCAAGG | 32284 | 9127 | 1453.0005 | 448.9864 | -1.69429155** |
| miR1023 | | AGCGAGAUUGGGAAAGUGCAU | 434 | 125 | 19.533 | 6.1492 | -1.66744291** |
| miR5380 | | GAGAAUGAGAUGGGGAUGGGGAA | 329 | 98 | 14.8072 | 4.8209 | -1.61892445** |
| miR535 | | UGACAAUGAGAGAGAGCACAC | 185022 | 57065 | 8327.2537 | 2807.2102 | -1.56870368** |
| miR6150 | | AGAAUUUGUUUGAAUCUGUUGGCA | 29 | 9 | 1.3052 | 0.4427 | -1.55986961** |
| miR5059 | | UCGUUCCUGGGCAGCAACACCA | 10610 | 3334 | 477.5225 | 164.0101 | -1.54178405** |
| miR5499 | | GAAGAAGCAAUCGUUUGGA | 41 | 13 | 1.8453 | 0.6395 | -1.52883912** |
| miR894 | | GUUUCACGUCGGGUUCACCA | 28609 | 9269 | 1287.6004 | 455.9718 | -1.49766842** |
| miR171 | | CGAGCCGAAUCAAUAUCACUC | 13357 | 4441 | 601.1562 | 218.467 | -1.46032453** |
| miR1101 | | UUAGAUUGCAGAGGAAUGCUCAA | 33 | 11 | 1.4852 | 0.5411 | -1.45669007** |
| miR4364 | | CGUAGAUCGGCAGCGGAAGAAGUU | 207 | 70 | 9.3164 | 3.4435 | -1.43589691* |
| miR399 | | GGGCACCUCUUACUUGGCAUG | 23 | 8 | 1.0352 | 0.3935 | -1.39547398* |
| miR1048 | | UGGAAGAUAGUGUAGAGAC | 298 | 104 | 13.412 | 5.1161 | -1.39040802* |
| miR834 | | UGAGUAGGAUCAGUAGGGUGGUAA | 125 | 45 | 5.6259 | 2.2137 | -1.34562419* |
| miR1877 | | AGAUGACAGUGGAUAUGGAGGGGG | 188 | 69 | 8.4613 | 3.3943 | -1.31776526* |
| miR1508 | | UGGAAGAGGAGAAUGCAGUUG | 27 | 10 | 1.2152 | 0.4919 | -1.30475681* |
| miR2084 | | CCUCAUUGAUGGAUUGUGUAA | 24 | 9 | 1.0802 | 0.4427 | -1.28689717* |
| miR1112 | | ACGGAAUGGUAUACAAAGUCA | 138 | 52 | 6.2109 | 2.558 | -1.27978608* |
| miR5203 | | GACUUAUCUAUUGGACGGAGGGA | 29 | 11 | 1.3052 | 0.5411 | -1.27030375** |
| miR5669 | | ACAAUGUGAGUGUGGUUAAGUGGG | 42 | 16 | 1.8903 | 0.7871 | -1.26399637** |
| miR2083 | | AGAAUUGAGAGUGCAGAGUAAGAG | 34 | 13 | 1.5302 | 0.6395 | -1.25870396* |
| miR5769 | | UGAUAGGGACAAUGAAGAGACGGA | 39 | 15 | 1.7553 | 0.7379 | -1.2502204** |
| miR6116 | | UCAUUGUACACAAGCUGAG | 7142 | 2748 | 321.4388 | 135.1829 | -1.24963141** |
| miR7756 | | UGGAAUGUGGAUUGAAUAUGG | 23 | 9 | 1.0352 | 0.4427 | -1.22550824** |
| miR5542 | | UUUGAGAGGAAUAUGAGCAU | 93 | 37 | 4.1856 | 1.8201 | -1.20141673** |
| miR157 | | UUGACGGAAGAUAGAGAGCAC | 1935764 | 770693 | 87122.601 | 37912.8583 | -1.2003598** |
| miR403 | | UUAGAUUCACGCACAAACUCG | 2596 | 1036 | 116.8377 | 50.9642 | -1.19694978** |
| miR858 | | CUCGUUGUCUGUUCGACCUUG | 837 | 338 | 37.6707 | 16.6273 | -1.17988892** |
| miR5061 | | UCUGAUCUCGCUCCUGCUCGGUAU | 32 | 13 | 1.4402 | 0.6395 | -1.17125291** |
| miR1446 | | CGAACUCUCUCCCUCAACGGC | 26042 | 10652 | 1172.0679 | 524.006 | -1.16140091** |
| miR5161 | | UUUUGAUAGAGUGGAGUAUA | 22914 | 9463 | 1031.2865 | 465.5153 | -1.14754469** |
| miR1167 | | GGGGCUGUGAUGAUAUUUAAA | 31 | 13 | 1.3952 | 0.6395 | -1.12545568** |
| miR8155 | | CGUAACCUGGCUCUGAUACCA | 288 | 121 | 12.962 | 5.9524 | -1.12274496** |
| miR7484 | | UUUGAUCAUCAAGAUAAAGAGCAA | 42 | 18 | 1.8903 | 0.8855 | -1.094051** |
| miR6478 | | CCGACCUUAGCUCAGUUGGCAG | 2082 | 914 | 93.7042 | 44.9626 | -1.05938825** |
| miR5675 | | UCAGAGACGGACAACUGGAAA | 66 | 29 | 2.9705 | 1.4266 | -1.05812491** |
| miR5640 | | UGAGAUGAAGGAAUUAGUAUA | 75 | 33 | 3.3755 | 1.6234 | -1.0560827** |
| miR5716 | | UUGAUAAUUGAGAGAGUACUGAAA | 70 | 31 | 3.1505 | 1.525 | -1.04677157** |
| miR4378 | | AUAGGACUACUUAGAAUGGCGA | 126 | 56 | 5.6709 | 2.7548 | -1.04163013** |
| miR2666 | | CAAAGUGUUGAUAAUCAAGGA | 36 | 16 | 1.6202 | 0.7871 | -1.04155307** |
| miR530 | | UGCAUUUGCACCUGCAUCUUG | 435 | 195 | 19.578 | 9.5927 | -1.02922455** |
| miR3951 | | UAGAUAAAGAUGAGAGAAAAA | 18102 | 8139 | 814.7136 | 400.3835 | -1.02491047** |
| miR5152 | | AGUCCUGCUAUACCCACCA | 31 | 14 | 1.3952 | 0.6887 | -1.01852436** |
| miR5054 | | GUUCCCCACAGACGGCGCCA | 4885 | 2217 | 219.8584 | 109.0613 | -1.0114354** |
|  | |  |  |  |  |  |  |
| ***Equally expressed miRNAs*** | | |  |  |  |  |  |
| miR5822 | | UGUCUGCGAGUCGGGUUG | 1244 | 2272 | 55.9885 | 111.767 | 0.99729185 |
| miR916 | | AACCAAGGUCAUCGGUUCGAUACU | 29 | 52 | 1.3052 | 2.558 | 0.97074537 |
| miR4413 | | AAGAGAAUUGAAAGGACU | 201 | 358 | 9.0464 | 17.6112 | 0.96107752 |
| miR5293 | | AGAUGUAGAAGGGAUGGAAGAAGA | 136 | 238 | 6.1209 | 11.708 | 0.93567895 |
| miR7821 | | AGAUGGGCAAGGGCAUUUGUG | 116 | 202 | 5.2208 | 9.937 | 0.92853947 |
| miR1092 | | AUGACAGGAAGCUUGGUGUUU | 13 | 22 | 0.5851 | 1.0823 | 0.88734533 |
| miR815 | | AAGGGUGAUGAGGAGGAGUGGG | 118 | 199 | 5.3108 | 9.7894 | 0.88229124 |
| miR8144 | | AAACAGAGAACGACUAAGAAAGA | 15 | 25 | 0.6751 | 1.2298 | 0.86525059 |
| miR7722 | | AAGGUAUAGGGAUGAGGG | 81 | 134 | 3.6456 | 6.5919 | 0.85453808 |
| miR3627 | | UCGCAGGGGAGAUGGGACUAAC | 7480 | 12241 | 336.6511 | 602.174 | 0.83892624 |
| miR7981 | | AUUAAUGUAAACUAGUCCUAGUA | 13 | 21 | 0.5851 | 1.0331 | 0.82022478 |
| miR160 | | GCGUACGAGGAGCCAAGCAUA | 5880 | 9478 | 264.6402 | 466.2532 | 0.8170814 |
| miR5662 | | AGAGUGUGAGCAAUUGGAGAGUG | 943 | 1518 | 42.4414 | 74.6753 | 0.81515888 |
| miR4246 | | AAACAAUUUUCAUUGUAA | 38 | 61 | 1.7103 | 3.0008 | 0.81109776 |
| miR5079 | | UUUGGAUAUGUUAUGUUUGGUGGU | 101 | 162 | 4.5457 | 7.9693 | 0.80995053 |
| miR1510 | | UUGUCGUUUUACCUACUCCCC | 48 | 76 | 2.1603 | 3.7387 | 0.79130504 |
| miR397 | | UCAUUGAGUGCAGCGUUGAUG | 1816 | 2855 | 81.7324 | 140.4466 | 0.7810417 |
| miR841 | | UACGAGCCACUUGAAGAUGAACA | 94 | 139 | 4.2306 | 6.8379 | 0.69269104 |
| miR6464 | | UUGAUUGAUUGUUGGAUUUU | 5136 | 7505 | 231.1551 | 369.195 | 0.67552182 |
| miR5198 | | GGGGUGAAGAGAUUGGGGA | 374 | 543 | 16.8326 | 26.7119 | 0.66622456 |
| miR6152 | | AUUGUAUUCAGAGGUAGUUCACGG | 31 | 45 | 1.3952 | 2.2137 | 0.66598778 |
| miR5052 | | CCCGUGGACGUAGGCAUA | 2517 | 3619 | 113.2822 | 178.0302 | 0.6522008 |
| miR5539 | | AAGAAAACGGGAUGGCGAGCU | 191 | 272 | 8.5963 | 13.3806 | 0.63835507 |
| miR2592 | | AGGCGGUUUAGACACGGUA | 33 | 44 | 1.4852 | 2.1645 | 0.54337658 |
| miR854 | | AGGAUGAGGAAGAGGAGGAGGAGC | 60 | 80 | 2.7004 | 3.9355 | 0.54337382 |
| miR6157 | | UUAGGUAAUGUAGGAUUUGCAAGA | 51 | 67 | 2.2953 | 3.2959 | 0.52198974 |
| miR4398 | | UGUAGCGGAGAGAGAAAGAGGAAA | 21 | 27 | 0.9451 | 1.3282 | 0.49093351 |
| miR5207 | | CAUUAAGGUGUUUGGACGUU | 1053 | 1332 | 47.3922 | 65.5253 | 0.46740242 |
| miR5014 | | UUGUACAAAUUUAUUUUGUAC | 34 | 43 | 1.5302 | 2.1153 | 0.46714206 |
| miR5492 | | AGACUAGGAGAAACAGAUAUGGUU | 411 | 509 | 18.4978 | 25.0393 | 0.43684053 |
| miR5237 | | UUAAAAGAUUGUAAGUGUUGGGAU | 104 | 128 | 4.6807 | 6.2967 | 0.42787163 |
| miR4385 | | AAUCGAUGUUGAAAAGUUUGAUGG | 201 | 243 | 9.0464 | 11.9539 | 0.40206569 |
| miR6146 | | UUUGGCACAAUAAAUACUUAAUCC | 29 | 35 | 1.3052 | 1.7218 | 0.39964668 |
| miR5149 | | GAGCAGCUGGAAGAUUUGGGCA | 381 | 458 | 17.1476 | 22.5305 | 0.39387266 |
| miR6466 | | UCAGUGGUAGAGCAUUUGACUGCA | 871 | 1046 | 39.2009 | 51.4561 | 0.39245534 |
| miR1314 | | UCUAGGCCAUAGAAUGUUAGGAGA | 75 | 90 | 3.3755 | 4.4274 | 0.3913585 |
| miR482 | | AGUGGGAGCGUGGGGUAAGAAG | 3544 | 4249 | 159.5042 | 209.0219 | 0.39005969 |
| miR8124 | | ACUUGGUACGUGGAGCGGU | 261 | 311 | 11.7468 | 15.2991 | 0.38117899 |
| miR1514 | | UUCAUCUUUGAACGUAGGCAUG | 55 | 64 | 2.4754 | 3.1484 | 0.34695718 |
| miR5791 | | UUGCGGAGACUGGAGAGACGAG | 19 | 22 | 0.8551 | 1.0823 | 0.3399354 |
| miR7511 | | AGAAGAUUUUGUUGUGUAGUGA | 38 | 43 | 1.7103 | 2.1153 | 0.30661288 |
| miR1873 | | UCACAUGGUACCAAGCUGAAGC | 25 | 28 | 1.1252 | 1.3774 | 0.29176612 |
| miR5179 | | UCUUGCUCAAGACCGCGCAAU | 158 | 176 | 7.1111 | 8.658 | 0.28396106 |
| miR2628 | | UCAGAACAGAAUGAUGAGGAA | 28 | 31 | 1.2602 | 1.525 | 0.27515653 |
| miR6021 | | UUGGAGAUGCUUGCUAUUGAA | 29 | 32 | 1.3052 | 1.5742 | 0.27034795 |
| miR6114 | | UGAAAGGAAUCAGAAUCGUCA | 64 | 70 | 2.8804 | 3.4435 | 0.2576065 |
| miR6257 | | UCUUAACUGUAAUGGAUUAGGGCA | 24 | 26 | 1.0802 | 1.279 | 0.24371781 |
| miR1120 | | AAUCUUGAUAUUGUGGGACGAG | 96 | 103 | 4.3207 | 5.0669 | 0.22983829 |
| miR1313 | | UGUCCACUGGAUUGUUGUUCG | 168 | 180 | 7.5611 | 8.8548 | 0.22786359 |
| miR6280 | | UUAGCAUGUAAGAUUCUUGGU | 159 | 170 | 7.1561 | 8.3628 | 0.22481251 |
| miR3953 | | UUGAGUUCUGCAAGCCGUCGA | 76 | 81 | 3.4205 | 3.9846 | 0.22022767 |
| miR5386 | | CGUCGGCUGUCGGCGGACUG | 2074 | 2189 | 93.3442 | 107.6839 | 0.20617028 |
| miR391 | | UUGGCAGGGAGAUAGUGACCA | 101 | 106 | 4.5457 | 5.2145 | 0.19802645 |
| miR2936 | | GUUGGAGAGAGAGAAGCACGAGAG | 62 | 65 | 2.7904 | 3.1976 | 0.19651753 |
| miR774 | | UGAGGAUGAAGAUGAGGAUGC | 495 | 516 | 22.2784 | 25.3837 | 0.18825675 |
| miR2097 | | UCUCUCUCUCUUCUGUCGGCUUUU | 29 | 30 | 1.3052 | 1.4758 | 0.17722633 |
| miR2108 | | UCAAUGUGUUGAUGUGGUCGG | 92 | 95 | 4.1406 | 4.6734 | 0.17463269 |
| miR1861 | | CUGAUCUUGAGGCAAGGAGCUGUUG | 779 | 786 | 35.0603 | 38.6659 | 0.14122345 |
| miR5227 | | ACUGAAGAUGAAGACGAUGAUGAA | 1106 | 1110 | 49.7776 | 54.6045 | 0.13352318 |
| miR6485 | | AGAAUGUAGAAGAGGUAA | 318 | 318 | 14.3122 | 15.6434 | 0.12830865 |
| miR5740 | | UGGAACAGGAAACAACAUUUGG | 248 | 246 | 11.1617 | 12.1015 | 0.11662911 |
| miR6118 | | UGGACGAUAUGGGUGGUUCGGAAA | 401 | 397 | 18.0477 | 19.5297 | 0.1138548 |
| miR472 | | UUUUUCCCACACCUCCCAUCCC | 309 | 303 | 13.9071 | 14.9055 | 0.10002316 |
| miR7488 | | UUUUGAUGUAGCAGGGGAAACAA | 196 | 192 | 8.8213 | 9.4451 | 0.09857479 |
| miR6267 | | UAGGAAUAGGUCAGGCAAUGU | 593 | 573 | 26.6891 | 28.1877 | 0.07881511 |
| miR1091 | | CGGCAUGUGAGGGAAGAGUUG | 408 | 394 | 18.3628 | 19.3821 | 0.07793883 |
| miR5013 | | UUUGUGACAGUCAGAGUGCCUU | 329 | 317 | 14.8072 | 15.5942 | 0.07471069 |
| miR5766 | | UUGAGGCAUGUAGAAGGGCAA | 13886 | 13293 | 624.9648 | 653.9253 | 0.06535091 |
| miR5244 | | UUAUCGGAUGAAGAUUGUUGG | 1670 | 1588 | 75.1614 | 78.1188 | 0.05567785 |
| miR5241 | | UACUGAAACGGAGGAGUGCAU | 76 | 72 | 3.4205 | 3.5419 | 0.05031625 |
| miR780 | | UUCUUCGGACACAUUUGGCAU | 35 | 33 | 1.5752 | 1.6234 | 0.0434835 |
| miR408 | | ACGGGGAACAGGCAGAGCAUG | 19871 | 18728 | 894.3307 | 921.2903 | 0.04284742 |
| miR2652 | | UUAUGCAGGGUGGAUUGGAUU | 225 | 211 | 10.1265 | 10.3798 | 0.03564302 |
| miR6263 | | AAUGGAAAAAGGGGAUGGA | 248 | 230 | 11.1617 | 11.3144 | 0.01960331 |
| miR5658 | | AUGAAUGAUGAAUGAUGAUGACUA | 39 | 36 | 1.7553 | 1.771 | 0.01284659 |
| miR528 | | UGGAAGGGAGCUGCAGAGAUG | 98 | 90 | 4.4107 | 4.4274 | 0.00545208 |
| miR5039 | | CCCUUUCUGAAUCGUUUGCUG | 76 | 35 | 3.4205 | 1.7218 | -0.99028966 |
| miR479 | | UGUGAUAUUGGUUCGGCUCAUC | 17953 | 8314 | 808.0076 | 408.9923 | -0.98229518 |
| miR5020 | | UGGCAUGAAGAUGAACUUGCA | 56 | 26 | 2.5204 | 1.279 | -0.97863645 |
| miR1526 | | CGGAAGUGGAAAAUUAAAGCA | 71 | 33 | 3.1955 | 1.6234 | -0.97702317 |
| miR5767 | | UGUGGACCUUUAAUGUGCA | 171 | 80 | 7.6962 | 3.9355 | -0.96759935 |
| miR7838 | | AGCAUGUGCUGGGAGGAGAGAGA | 969 | 454 | 43.6116 | 22.3337 | -0.96548964 |
| miR5294 | | GCUAGAAUCGGAAUGAGUUAGCAU | 223 | 105 | 10.0365 | 5.1653 | -0.9583322 |
| miR5632 | | UUUAUUGAUAGUUGGAUAAGU | 131 | 62 | 5.8959 | 3.05 | -0.95090281 |
| miR4993 | | GGCGGCGGUGGUGGAGGAGG | 221 | 105 | 9.9465 | 5.1653 | -0.94533681 |
| miR162 | | UCGAUAAACCUCUGCAUCCAG | 1533 | 730 | 68.9955 | 35.911 | -0.94207644 |
| miR7743 | | UUAUCUUUUGUAUUGAUCUUU | 44 | 21 | 1.9803 | 1.0331 | -0.9387391 |
| miR3444 | | AUCUCAUCAAUUCGUCACAAUACC | 25 | 12 | 1.1252 | 0.5903 | -0.93066121 |
| miR5239 | | UGGGAGUGGAAGAAGAAUGUG | 56 | 27 | 2.5204 | 1.3282 | -0.92418031 |
| miR4346 | | UAAACCCAACGAGAGAGCUGCAU | 31 | 15 | 1.3952 | 0.7379 | -0.91897472 |
| miR818 | | AGGAGCAUUAGGAUGGACCAU | 1355 | 658 | 60.9843 | 32.3691 | -0.91382063 |
| miR2089 | | UUACCUAUGCCACCCAUUCC | 1558 | 761 | 70.1206 | 37.436 | -0.90541205 |
| miR7502 | | UUUUGAAAAGUAGAGAAUGAAUAA | 571 | 279 | 25.6989 | 13.7249 | -0.90491097 |
| miR167 | | UGAAGCUGCCAGCAUGAUCUGA | 780656 | 384075 | 35134.852 | 18893.88 | -0.89498381 |
| miR169 | | CAGCCAAGGAUGACUUGCCGG | 802 | 396 | 36.0955 | 19.4805 | -0.88978828 |
| miR8043 | | UGAUGAUAAUUGGACAUUGCA | 118 | 59 | 5.3108 | 2.9024 | -0.87168284 |
| miR5641 | | UGGAAGAAGAUGUAGGUUUAA | 40 | 20 | 1.8003 | 0.9839 | -0.87165374 |
| miR829 | | AGCUCUGAUACCAAAUGAUACGAA | 36 | 18 | 1.6202 | 0.8855 | -0.8716077 |
| miR5033 | | GCUGGACAAAUAGCAAACUAC | 10030 | 5020 | 451.4185 | 246.9499 | -0.87024716 |
| miR6196 | | AGGAGAGGUAUAGAUGGACGAGGA | 179 | 90 | 8.0562 | 4.4274 | -0.86363978 |
| miR5265 | | AAGUGAUGUGUGGAUGAUGGUUGA | 103 | 52 | 4.6357 | 2.558 | -0.85777094 |
| miR1134 | | CAGAACAAAGAAGAAGAAGAAGAU | 412 | 209 | 18.5428 | 10.2814 | -0.85082238 |
| miR1445 | | UCCCUUGUAGAUCUAGUAGAAGAA | 4860 | 2477 | 218.7332 | 121.8516 | -0.84404702 |
| miR3514 | | AGGAUUCUGGAAUUAAACGGUGAA | 129 | 66 | 5.8059 | 3.2468 | -0.8385012 |
| miR1887 | | UAUUAUGUAGAAGUCAUAAGAGA | 285 | 146 | 12.8269 | 7.1822 | -0.83667481 |
| miR6432 | | CCGACUUGGAGAAAAAGAUGG | 232 | 119 | 10.4416 | 5.854 | -0.83484815 |
| miR5522 | | AAGAUAGGAAUGGGGAGGCUU | 33 | 17 | 1.4852 | 0.8363 | -0.82856475 |
| miR6477 | | UAAACAGUAGAAGGAAUUCAU | 31 | 16 | 1.3952 | 0.7871 | -0.8258531 |
| miR7760 | | GGCUUUGGUCGGAGUGGUGGGCUG | 399 | 209 | 17.9577 | 10.2814 | -0.80456586 |
| miR8122 | | UAAGGAAGGUUUUGUGGACAAG | 1649 | 865 | 74.2163 | 42.5521 | -0.80250575 |
| miR165 | | UCGGACCAGGCUUCAUCCCCC | 945 | 501 | 42.5315 | 24.6458 | -0.78718993 |
| miR390 | | AAGCUCAGGAGGGAUAGCGCC | 2734 | 1453 | 123.0487 | 71.4777 | -0.7836643 |
| miR5541 | | UCAAAGUGGUGUAUGUAAUGA | 56 | 30 | 2.5204 | 1.4758 | -0.77215549 |
| miR6170 | | AAGAAACAGGAAGAGAGAGUGAGU | 41 | 22 | 1.8453 | 1.0823 | -0.76975493 |
| miR2621 | | ACCGCUUGGGCGUUGGAAUUUGGC | 42 | 23 | 1.8903 | 1.1314 | -0.74050614 |
| miR5673 | | UCUUGGAAUCUCGGGAAGCAU | 31 | 17 | 1.3952 | 0.8363 | -0.73837948 |
| miR2101 | | AUUUGAACUCAAGUGACCGUUGGU | 69 | 38 | 3.1055 | 1.8693 | -0.73232745 |
| miR5772 | | UAGGAAUGUGAGUAGAGUAAGCAU | 315 | 174 | 14.1772 | 8.5596 | -0.72795734 |
| miR1108 | | UGAGCAAAUGAGAGAAUGAACCCC | 25 | 14 | 1.1252 | 0.6887 | -0.70823388 |
| miR6273 | | AGUGCAGUCGAUGAUUUUUAUUU | 23 | 13 | 1.0352 | 0.6395 | -0.69489326 |
| miR3699 | | ACAGAAGAUAGACAUUUGAAC | 3181 | 1799 | 143.1667 | 88.4986 | -0.69396943 |
| miR6484 | | UGAUGAGCUCUGCAAGAAUGG | 535 | 303 | 24.0787 | 14.9055 | -0.69191273 |
| miR2275 | | AGAAUUGGCAAGGAACAAAAGUGA | 30 | 17 | 1.3502 | 0.8363 | -0.69108066 |
| miR7707 | | UUUGAUCGUAUGAUUAUGAACGG | 60 | 34 | 2.7004 | 1.6726 | -0.69108066 |
| miR7726 | | UGUUGCAUGUCGGACGUCACGGU | 352 | 201 | 15.8424 | 9.8878 | -0.68006944 |
| miR7822 | | UGAGACAAUUGAAAAAUGGUA | 42 | 24 | 1.8903 | 1.1806 | -0.67909497 |
| miR8151 | | UGGAUACCAGUAGACCCCAU | 189 | 109 | 8.5063 | 5.3621 | -0.66573361 |
| miR161 | | UUAAUGACUUAAAGUGACUUA | 86 | 50 | 3.8706 | 2.4597 | -0.65407486 |
| miR5261 | | UCUAUUGUAGAUGUUUUGGAU | 24 | 14 | 1.0802 | 0.6887 | -0.64935087 |
| miR3947 | | AAUGAUUUAGUAGACGACGUUACA | 253 | 148 | 11.3867 | 7.2806 | -0.64522044 |
| miR5770 | | UUCAGGAUAUGGUUUUGAUAA | 112329 | 66174 | 5055.5722 | 3255.3111 | -0.63507896 |
| miR5631 | | UGACGAGGAAGAGAUAAUUUU | 463 | 273 | 20.8382 | 13.4297 | -0.63380359 |
| miR166 | | UCGGACCAGGCUUCAUUCCCC | 524493 | 309678 | 23605.767 | 15234.0531 | -0.63183954 |
| miR5291 | | GUAUGUAUGUAUGGAUGGAUGGAU | 27 | 16 | 1.2152 | 0.7871 | -0.62657493 |
| miR393 | | UCAUGCGAUCCCUUCGGAAUU | 1429 | 847 | 64.3148 | 41.6666 | -0.62625939 |
| miR6462 | | AAAGGACAAAAUAUGCAUGAAGAA | 92 | 55 | 4.1406 | 2.7056 | -0.61389127 |
| miR3948 | | GGAGUGGGAGUGGGAGUAGGUUGU | 7760 | 4651 | 349.253 | 228.7976 | -0.61020059 |
| miR5835 | | ACAAACUCAUCCGGAUUUUGGCAU | 100 | 60 | 4.5007 | 2.9516 | -0.60865218 |
| miR6019 | | AACAGGUGGACGGUUGUAAAUUUU | 2175 | 1305 | 97.8899 | 64.1971 | -0.60865189 |
| miR5254 | | AGCGGUGGAAGCAAUUGUGUA | 35150 | 21140 | 1581.9901 | 1039.9443 | -0.60523431 |
| miR5139 | | AACCUGGCUCUGAUACCA | 1531 | 926 | 68.9055 | 45.5529 | -0.59707624 |
| miR6260 | | UGGAGUGGAGAAUGGGGUAGGCCU | 468 | 284 | 21.0632 | 13.9709 | -0.59229967 |
| miR4345 | | UUAAGACGGAACUUACAAAGAAUA | 70 | 43 | 3.1505 | 2.1153 | -0.57471852 |
| miR6269 | | UGUGAAUAAGUGAUUGUCUGA | 70 | 43 | 3.1505 | 2.1153 | -0.57471852 |
| miR7496 | | AGACCAAAUUGUUAGACGAUGUGU | 1031 | 634 | 46.402 | 31.1885 | -0.57317282 |
| miR4393 | | UUUGAAUAAGGGACACAGAGACC | 570 | 351 | 25.6539 | 17.2668 | -0.57117743 |
| miR5534 | | CUUUAGACAACAGUAGAAUGG | 2507 | 1556 | 112.8321 | 76.5446 | -0.55980505 |
| miR860 | | UCAAUUAGAUUGGAUAUAUGGAUA | 217 | 135 | 9.7665 | 6.6411 | -0.55641942 |
| miR6288 | | AACCGAAUUAGAAAAAACCGUCGG | 90 | 56 | 4.0506 | 2.7548 | -0.55618804 |
| miR6448 | | UAGGGACACAAUUAACAAAUGGC | 270 | 168 | 12.1518 | 8.2645 | -0.55617059 |
| miR1534 | | UAUUUAGGGGUAAAAUUGUCAU | 53 | 33 | 2.3854 | 1.6234 | -0.55521269 |
| miR8138 | | UAAAGAUGGGAACAAAACAA | 682 | 426 | 30.6947 | 20.9563 | -0.55060555 |
| miR4406 | | ACCUUGUACUAGAGAACCGGUGUA | 1520 | 955 | 68.4104 | 46.9795 | -0.54218431 |
| miR1520 | | GUCACGAUCCUGUUGGACUAA | 752 | 473 | 33.8451 | 23.2684 | -0.54057497 |
| miR862 | | CUGGAUCUGGUUUGAAGGA | 27 | 17 | 1.2152 | 0.8363 | -0.53910131 |
| miR3704 | | GGAUCUGGUGGAGAUUGUGGAGGA | 73 | 46 | 3.2855 | 2.2629 | -0.53794011 |
| miR1033 | | AUAGACGGGUCGAUGAUGUGGCAU | 282 | 178 | 12.6919 | 8.7564 | -0.53549829 |
| miR5644 | | GUGAAGUGGCGGAUAACGGUA | 176 | 112 | 7.9212 | 5.5096 | -0.52377142 |
| miR832 | | UUUAUUCCCAAUCCAAGACAAAGU | 132 | 84 | 5.9409 | 4.1322 | -0.52377142 |
| miR1875 | | AGCAAUGGAGCUGAAUGAAGAGAA | 36 | 23 | 1.6202 | 1.1314 | -0.51806284 |
| miR2948 | | UAGUGGGAGAUUGUUGGGAAAAU | 172 | 110 | 7.7412 | 5.4113 | -0.516582 |
| miR1171 | | AGUGUGGAGUGGGAGUGGGAGUGG | 6139 | 3970 | 276.2969 | 195.297 | -0.50054959 |
| miR473 | | UGAGGCCGUUGGGGAGAGUGG | 474 | 307 | 21.3332 | 15.1023 | -0.49833211 |
| miR6262 | | UCUUUAGACAACGGUAGAAUGGU | 16467 | 10751 | 741.1275 | 528.8761 | -0.48679198 |
| miR1144 | | UGGGUUUAUGUGCGGCAGGCAG | 173 | 113 | 7.7862 | 5.5588 | -0.48614593 |
| miR396 | | UUCCACAGCUUUCUUGAACUG | 3868 | 2527 | 174.0864 | 124.3112 | -0.48584722 |
| miR5788 | | UGGAUGUAGAGCAUACUCAGUAUA | 52 | 34 | 2.3404 | 1.6726 | -0.48466266 |
| miR7535 | | UGGAAAAUGACUGGGGGUGGU | 113 | 74 | 5.0858 | 3.6403 | -0.48241738 |
| miR5303 | | AACUAGUUUUUGAGGCUGAGCAA | 53 | 35 | 2.3854 | 1.7218 | -0.47031363 |
| miR3952 | | UGAAGGGCCUUUCUAGAGCAC | 675 | 447 | 30.3796 | 21.9894 | -0.46629463 |
| miR902 | | AUGAAGGUCGUGUAAUCGUGC | 128 | 85 | 5.7609 | 4.1814 | -0.46230815 |
| miR8051 | | GAUAGUAUGGUAGAAAGAUUCA | 295 | 196 | 13.277 | 9.6419 | -0.46153983 |
| miR7997 | | AUUUGAUCGGGACCUAUCAAAAAU | 39 | 26 | 1.7553 | 1.279 | -0.45670136 |
| miR6025 | | UACCAACAAGAGAUGAACAUU | 250 | 167 | 11.2517 | 8.2153 | -0.45375783 |
| miR1858 | | GUGAUGAGGAGGAGUGGGGUC | 338 | 227 | 15.2123 | 11.1669 | -0.44600956 |
| miR5234 | | UUUUGUUAUGGAUGGCUGAAG | 889 | 598 | 40.0111 | 29.4175 | -0.44372565 |
| miR7696 | | UUCAAAUGAGAACUUUGAAG | 7917 | 5345 | 356.3191 | 262.9377 | -0.43844881 |
| miR1857 | | UGGAUUUUUUUGAAGCAUCGG | 359 | 243 | 16.1575 | 11.9539 | -0.43472261 |
| miR4375 | | AGCACUAUGGUCGGGCCUGGCAU | 56 | 38 | 2.5204 | 1.8693 | -0.43115459 |
| miR5655 | | AAGUAGAGACUGAUGAAGAAGGAG | 53 | 36 | 2.3854 | 1.771 | -0.429667 |
| miR830 | | UAACAUUUUGAAGACAGAAGUGGA | 198 | 135 | 8.9114 | 6.6411 | -0.42422988 |
| miR6195 | | UGAGACGAGAUGGGAUGAG | 797 | 544 | 35.8704 | 26.7611 | -0.42265641 |
| miR8003 | | AAUUUCUGGUAACAAAUGGGAGUC | 205 | 140 | 9.2264 | 6.887 | -0.42189216 |
| miR6455 | | UAAAGAUAGCAUCCUCAAUUU | 57 | 39 | 2.5654 | 1.9185 | -0.41920503 |
| miR5562 | | AUGAUGGAGAAUAGGCUGUCAAC | 51 | 35 | 2.2953 | 1.7218 | -0.41476516 |
| miR159 | | UUUGGAUUGAAGGGAGCUCUA | 5295 | 3647 | 238.3112 | 179.4076 | -0.40960575 |
| miR1863 | | AGAGUUUGUGGCUGUAUCAUUACU | 1210 | 834 | 54.4583 | 41.0271 | -0.40857477 |
| miR5340 | | UUGGAUGACAUGAUUAAUUUCAAA | 56 | 39 | 2.5204 | 1.9185 | -0.39367395 |
| miR3633 | | UUACCUAUGCCACCCAUUCCUU | 3141 | 2197 | 141.3665 | 108.0775 | -0.38737407 |
| miR4403 | | ACGACACGAACACGACCCGAUGAC | 1400 | 985 | 63.0096 | 48.4553 | -0.37891718 |
| miR172 | | AGAAUCUUGAUGAUGCUGCAU | 74690 | 52745 | 3361.5601 | 2594.6956 | -0.37356565 |
| miR1159 | | ACAAUGACAUAGAUGACGGAU | 4032 | 2857 | 181.4675 | 140.545 | -0.36867906 |
| miR5524 | | GAAAAAUGUGGAUUCAUGACGG | 558 | 396 | 25.1138 | 19.4805 | -0.36644963 |
| miR6171 | | AUUGUGGACGGCUGAAGGUUU | 1687 | 1201 | 75.9265 | 59.081 | -0.36190926 |
| miR850 | | UAAAGAUCCGGAAACAACCAAG | 866 | 617 | 38.9759 | 30.3522 | -0.36078125 |
| miR5169 | | UUUGACCAAAGUUUGAGAGACAUU | 98 | 70 | 4.4107 | 3.4435 | -0.35713196 |
| miR6029 | | UGGGUUGUAAUUUGAUGGCUU | 823 | 592 | 37.0406 | 29.1224 | -0.34697821 |
| miR1040 | | UGAACCAGAUGAACAUGUG | 37 | 27 | 1.6653 | 1.3282 | -0.3263097 |
| miR5269 | | AGAAGAUGGUGGGACAACUUGCUU | 759 | 556 | 34.1602 | 27.3514 | -0.32070174 |
| miR1518 | | UGUGUUGUAAAGGGAAUAGUCCAC | 86 | 63 | 3.8706 | 3.0992 | -0.32066136 |
| miR5021 | | UGGGCCAAGAAGAAGAAAGAAAA | 151 | 111 | 6.796 | 5.4604 | -0.31567921 |
| miR6300 | | GUCGUUGUAGUAUAGUGGUG | 2639 | 1940 | 118.773 | 95.4348 | -0.31561957 |
| miR2950 | | UGGUAGACGGAGAUGGAAUA | 160 | 118 | 7.2011 | 5.8048 | -0.31097094 |
| miR5081 | | UUUUGUUGCAGAAUUGAUAGU | 69 | 51 | 3.1055 | 2.5089 | -0.3077706 |
| miR5494 | | UUGAUUUGGGUAUAGACGGUA | 74 | 55 | 3.3305 | 2.7056 | -0.29979022 |
| miR168 | | UCGCUUGGUGCAGGUCGGGAA | 55988 | 41638 | 2519.8424 | 2048.3066 | -0.29890182 |
| miR7825 | | UUGGAAGAAAUGGUAGAAGAGGAG | 216 | 161 | 9.7215 | 7.9201 | -0.29566029 |
| miR1850 | | UGGAAAGUAGAAGAGAUUGGG | 2115 | 1581 | 95.1894 | 77.7745 | -0.29150371 |
| miR7504 | | AGGGAAAAAUCUGAUCUGUGCAUU | 24 | 18 | 1.0802 | 0.8855 | -0.28673424 |
| miR477 | | ACCUCCCUCGAAGGCUUCCAA | 772 | 579 | 34.7453 | 28.4829 | -0.2867218 |
| miR1513 | | UUUAAUGUGUAGAGAUUCAAUGGU | 120 | 91 | 5.4008 | 4.4766 | -0.27076971 |
| miR1515 | | UCAUUUUUGCGUGCAAUGAUCC | 324 | 247 | 14.5822 | 12.1507 | -0.26316896 |
| miR3515 | | AAAUGUAGAGCAAAAUGAAGGUAU | 354 | 270 | 15.9324 | 13.2822 | -0.26246948 |
| miR7508 | | CAAAGAAGAAGCGGGAGGAG | 31 | 24 | 1.3952 | 1.1806 | -0.2409517 |
| miR5719 | | UUGUGAUGAAAAUAGACGUCC | 3258 | 2532 | 146.6323 | 124.5572 | -0.23539452 |
| miR3950 | | UAUUUUCUGCAACAUGAUUGU | 2460 | 1917 | 110.7168 | 94.3034 | -0.23149246 |
| miR861 | | CUUGGAGAAAUUAUGAGCGUCAGA | 1066 | 831 | 47.9773 | 40.8796 | -0.23097089 |
| miR845 | | UGGCUCUGAUACCAAACUGAUGCA | 87 | 68 | 3.9156 | 3.3451 | -0.22718405 |
| miR8034 | | UAUGACAGAAGAUCUUCAAAAACU | 298 | 233 | 13.412 | 11.462 | -0.22666559 |
| miR5272 | | AGAAAUUGUUAUGUUCGGAUAACU | 74 | 58 | 3.3305 | 2.8532 | -0.2231579 |
| miR3705 | | GAGGUGGUUAUGAGCGGAC | 142 | 112 | 6.391 | 5.5096 | -0.21409411 |
| miR5657 | | UGGACAAGGAAGAUUGAGGUG | 3716 | 2933 | 167.2454 | 144.2837 | -0.21305821 |
| miR6035 | | UGAAGUAAAAAUGCAUGACGU | 96 | 76 | 4.3207 | 3.7387 | -0.20872835 |
| miR1169 | | UCUCCAGUCUAAAGCAACAUCACA | 29 | 23 | 1.3052 | 1.1314 | -0.20616182 |
| miR831 | | AUCAAGAUUACAAGGAGAUGGAGA | 166 | 132 | 7.4711 | 6.4935 | -0.20232437 |
| miR440 | | AUGUCACUGAUGAUCGAAGGACAA | 441 | 351 | 19.848 | 17.2668 | -0.2009929 |
| miR3954 | | UUGGACAGAGAAAUCACGGUCA | 116108 | 92662 | 5225.653 | 4558.3407 | -0.19710257 |
| miR2636 | | UUUGUGUUGAAGAUGGCUGAAUAU | 1364 | 1089 | 61.3893 | 53.5714 | -0.19652422 |
| miR6295 | | AGGACAGGAGAUGAUUCAUGA | 1373 | 1098 | 61.7944 | 54.0141 | -0.19414004 |
| miR1028 | | UGCACUUGUAGGGUUUAAGGAGGC | 25 | 20 | 1.1252 | 0.9839 | -0.19359786 |
| miR5250 | | UGAUGCAUGUUGAUACGGAUC | 603 | 483 | 27.1391 | 23.7603 | -0.19181983 |
| miR827 | | UUAGAUGACCAUCAACAAACA | 170 | 137 | 7.6512 | 6.7395 | -0.18304447 |
| miR7773 | | UUAUUCCAUUCGUCGACACGU | 351 | 283 | 15.7974 | 13.9217 | -0.18235174 |
| miR3630 | | GAACAAGUGAUGGAUAGACCUGCA | 286 | 231 | 12.872 | 11.3636 | -0.17981628 |
| miR1103 | | UGGAAAAUGGAUGGUGCAGUUUU | 110 | 89 | 4.9508 | 4.3782 | -0.17732381 |
| miR7129 | | AGAAAUCUAGAGAUCGUGUAU | 883 | 715 | 39.741 | 35.1731 | -0.17615568 |
| miR952 | | ACAGAACAUGGCAUUGGUCCG | 1497 | 1219 | 67.3752 | 59.9665 | -0.16806088 |
| miR5376 | | UGAGAGGGUUUGAAGAAUUUGGGC | 560 | 457 | 25.2038 | 22.4813 | -0.1649158 |
| miR6483 | | UUAUUGUAGAAAUUUUCGGGA | 34 | 28 | 1.5302 | 1.3774 | -0.15177265 |
| miR2086 | | UCACAUUUAAUGCAGAACUGGAA | 145 | 120 | 6.526 | 5.9032 | -0.14470177 |
| miR5741 | | UAGGGACUGAAAUUGGAUGGCUU | 99 | 82 | 4.4557 | 4.0338 | -0.14351254 |
| miR826 | | UUGCUGGUUUUGGAUGACGUG | 152 | 126 | 6.841 | 6.1983 | -0.14233465 |
| miR7122 | | UUGGACAGAGAAAUCACGGUCG | 1476 | 1224 | 66.4301 | 60.2125 | -0.14177407 |
| miR5532 | | UGGAAUGUAUGACAAAGGUGGAGU | 30 | 25 | 1.3502 | 1.2298 | -0.13474941 |
| miR5062 | | UGAACCUCUGGAGAAGAAGCCCC | 469 | 393 | 21.1082 | 19.3329 | -0.1267455 |
| miR7814 | | AAUUGAUUUUUAUAGCUUUGA | 44 | 37 | 1.9803 | 1.8201 | -0.12170129 |
| miR5713 | | UAUGAGCUUCAGAAGAACUUUGUU | 244 | 207 | 10.9817 | 10.183 | -0.10893875 |
| miR6140 | | AAGUUUGUAGAAGAGUUUGUGGCU | 655 | 557 | 29.4795 | 27.4006 | -0.10550457 |
| miR900 | | UCUCCAGGCAAAGAACACAC | 34 | 29 | 1.5302 | 1.4266 | -0.10113935 |
| miR8135 | | AGAAUUUUGCAGUGGUUGGAU | 1263 | 1085 | 56.8436 | 53.3746 | -0.09084457 |
| miR5565 | | UUUUGUUGGAAGAUUGUCGGA | 287 | 247 | 12.917 | 12.1507 | -0.08823161 |
| miR1078 | | CUUGAUUGAUUGUUGGAU | 1431 | 1236 | 64.4048 | 60.8028 | -0.08303045 |
| miR5231 | | UUUGCAAGUUGUAAGCUCAU | 35 | 31 | 1.5752 | 1.525 | -0.04672577 |
| miR7984 | | UCCGACUUUGUGAAAUGACUU | 429 | 380 | 19.3079 | 18.6934 | -0.04666227 |
| miR3446 | | CUGGAAGCAACUGUGGCACGG | 14904 | 13204 | 670.7818 | 649.5471 | -0.0464094 |
| miR909 | | UGUGGUCAAAGAGUGUGGUGG | 38 | 34 | 1.7103 | 1.6726 | -0.03215694 |
| miR8126 | | UCUGACUCCCAGAUUACUGACAUA | 106 | 96 | 4.7707 | 4.7225 | -0.01465017 |
| miR8148 | | UAGACGGAUCGAUGACGUGGCAU | 1508 | 1369 | 67.8703 | 67.3455 | -0.01119884 |
| miR2099 | | GAAUUGCAUGUACAAGCUUU | 140 | 128 | 6.301 | 6.2967 | -0.00098488 |

** and ** indicate a significant difference at P < 0.05 and P < 0.01, respectively.*

**Table S4 | List of known miRNAs in *Citrus sinensis* leaves after removing these miRNAs with normalized read-count less than 10 TPM in the two miRNA libraries constructed from Mg-sufficient and -deficient leaves.**

| **miRNA** | ***Sequence*** | **Expressed** | | **Normalized read count** | | **Fold change** |
| --- | --- | --- | --- | --- | --- | --- |
|  |  | **Control** | **Mg-deficiency** | **Control** | **Mg-deficiency** |  |
| ***Up-regulated miRNAs*** | |  |  |  |  |  |
| miR5832 | UUGGCGGAGCGAUUUGUC | 0 | 40754 | 0.01 | 2004.8199 | 17.61311311** |
| miR5493 | ACCGGGCUCGAGCGACGCGUG | 0 | 25261 | 0.01 | 1242.6695 | 16.92308312** |
| miR1104 | CGCAGCUGUUCGUCUUUCCC | 0 | 4433 | 0.01 | 218.0735 | 14.41252685** |
| miR5760 | UGUUUAAGAUAGUUUGUAAGGAU | 0 | 3899 | 0.01 | 191.8043 | 14.22734744** |
| miR3443 | GUGCGUUUGAGAUUGAGGUU | 0 | 2668 | 0.01 | 131.2475 | 13.68000232** |
| miR5666 | AGGGACAUAGAGACAUUUACU | 0 | 2555 | 0.01 | 125.6886 | 13.61756618** |
| miR7730 | AUGAACACGGCACGAUUGAAGUUAU | 0 | 1366 | 0.01 | 67.1979 | 12.71420043** |
| miR6166 | AUUAGAUAACGGAUUAAUGGA | 0 | 1131 | 0.01 | 55.6375 | 12.44184188** |
| miR6233 | UCAAGUUGUUUUGGAAUUACUG | 0 | 1045 | 0.01 | 51.4069 | 12.3277463** |
| miR4408 | AACAAAUAUGGAUGAUUGUUGGA | 0 | 1044 | 0.01 | 51.3577 | 12.32636488** |
| miR8039 | UUUCCUACUGAGAUUAUCAAC | 0 | 956 | 0.01 | 47.0287 | 12.19932574** |
| miR5160 | CAGAAGAUCGAUGGUAAUUUCUU | 0 | 944 | 0.01 | 46.4384 | 12.18110255** |
| miR1851 | AUAUGGGAUGGCAUUUGGC | 0 | 840 | 0.01 | 41.3223 | 12.01270484** |
| miR917 | UUUGGACGGUUAUUUUCGAA | 0 | 814 | 0.01 | 40.0433 | 11.96734516** |
| miR1077 | UUGAAGUGUUCGGAUCGCGGC | 22 | 74084 | 0.9902 | 3644.4294 | 11.84568538** |
| miR4223 | AUGGUUUUGAUAAAUAGAACAGGC | 0 | 578 | 0.01 | 28.4337 | 11.47338613** |
| miR1172 | UAGACGGAGACAUAGUGAA | 0 | 432 | 0.01 | 21.2515 | 11.05334896** |
| miR5821 | UGCGGAGGGAUGGUGGGAG | 0 | 379 | 0.01 | 18.6442 | 10.86451118** |
| miR1162 | CGGCUUAAUUUGACUCAACACG | 0 | 359 | 0.01 | 17.6604 | 10.7863023** |
| miR6449 | CUAUGAUUCUGGAAAUAAACGGUU | 0 | 272 | 0.01 | 13.3806 | 10.38592709** |
| miR395 | GUGAAGUGUUCGGAUCGCC | 64 | 73994 | 2.8804 | 3640.002 | 10.30345436** |
| miR946 | UGUGGAAUAGAAGGGCUGAGU | 0 | 255 | 0.01 | 12.5443 | 10.29281625** |
| miR2610 | AAGAUUUAGACAUUGUAUGGCGUU | 7 | 2833 | 0.315 | 139.3643 | 8.7892935** |
| miR5774 | GCUUGGCCUACGACACGUGCAU | 11 | 3200 | 0.4951 | 157.4183 | 8.3126676** |
| miR5540 | UUGUGCGAAUCAACGGUUCUA | 14 | 3488 | 0.6301 | 171.5859 | 8.08913448** |
| miR3513 | UUGGAUAAGAUAGGAUUGGAU | 1 | 234 | 0.045 | 11.5112 | 7.99889752** |
| miR812 | ACGGCACGAUUAAAGAUGGGCCAU | 5 | 1041 | 0.225 | 51.2101 | 7.83035956** |
| miR3438 | UCAAGGAUUGAGAUAGAUAGC | 13 | 2429 | 0.5851 | 119.4903 | 7.67399457** |
| miR5776 | ACUAUGGGCUGACCUUAGGUGG | 11 | 1393 | 0.4951 | 68.5261 | 7.11278982** |
| miR6228 | GUGGAUAGUAGAAUGAUGAAGGA | 3 | 349 | 0.135 | 17.1684 | 6.9906523** |
| miR5251 | AGUAGAUCAGUUGGAGCUU | 2 | 329 | 0.09 | 16.1846 | 7.49048099** |
| miR5286 | ACAAUGGAGGCAAGGGAAGUA | 30 | 2988 | 1.3502 | 146.9893 | 6.7663942** |
| miR529 | GAGGAAGGAGAGAUGGAGCAG | 60 | 5700 | 2.7004 | 280.4013 | 6.6981761** |
| miR4387 | ACUGAGCACUGAUUGGAUGAUGAU | 4 | 327 | 0.18 | 16.0862 | 6.48168285** |
| miR5181 | CUUUUUGGAUUGAAGGGA | 4 | 309 | 0.18 | 15.2007 | 6.39999705** |
| miR8019 | AAAAGAAGACCCUUUUGAGCUUG | 47 | 3599 | 2.1153 | 177.0463 | 6.3871206** |
| miR1168 | UCGUGGACAAGGCCAAGGCGC | 19 | 1338 | 0.8551 | 65.8205 | 6.26630003** |
| miR2616 | AUUGGGUUGGGUCGGCCGGU | 570 | 39850 | 25.6539 | 1960.3492 | 6.25578869** |
| miR6261 | AAGUGUUAGGUAUAGAGAAGCACG | 7 | 484 | 0.315 | 23.8095 | 6.24004169** |
| miR4366 | CUUGUUGUAGAGUUUGUUGG | 17 | 1154 | 0.7651 | 56.769 | 6.2133112**** |
| miR8127 | CAACUGUGGGAGAUACCUUUA | 60 | 3359 | 2.7004 | 165.24 | 5.93524603** |
| miR833 | UGAUUUGUUGAUAAUCGGCUCAGU | 17 | 849 | 0.7651 | 41.765 | 5.7705023** |
| miR7510 | AGAACUGAGACUAUUAGCGGCGU | 11 | 536 | 0.4951 | 26.3676 | 5.7349025** |
| miR1440 | UUAAGGAGAGGUUGGCUAUUUGAG | 6 | 248 | 0.27 | 12.1999 | 5.4977661** |
| miR1160 | UGAAGGAAGCAGUAGUGGAU | 11 | 413 | 0.4951 | 20.3168 | 5.35880943** |
| miR1512 | UAAGCAGAACAUUCAUGAGCAU | 28 | 813 | 1.2602 | 39.9941 | 4.98806257** |
| miR5290 | AAUAUGAGUAGAGUAGACACCUA | 57 | 1386 | 2.5654 | 68.1818 | 4.73213099** |
| miR5337 | CUAGAACGACGAAGACAUAUUGUGA | 53 | 1264 | 2.3854 | 62.1802 | 4.70415214** |
| miR7758 | AACCGUUAGUUGACCGUGUAA | 24 | 533 | 1.0802 | 26.22 | 4.60129733** |
| miR5304 | AUGAUGAGUCUGGUAAUUGGA | 64 | 1350 | 2.8804 | 66.4108 | 4.5270768** |
| miR7785 | GUGAAUGGGUAGAGAGAGAAGAC | 22 | 420 | 0.9902 | 20.6611 | 4.38305331** |
| miR1533 | AUAAUAAAAAUAGAUCAUGUG | 18 | 335 | 0.8101 | 16.4797 | 4.34644616** |
| miR6426 | AGUGGAAGAUGGAAGUGUAGA | 188 | 3403 | 8.4613 | 167.4045 | 4.30631516** |
| miR5037 | AUGAGAACUUUGAAGGCCGGC | 1155 | 19027 | 51.9829 | 935.9991 | 4.17039812** |
| miR3520 | AGGUGAUCGGUGAAUAAUUAU | 79 | 1257 | 3.5555 | 61.8359 | 4.1203203** |
| miR1026 | UGUGAAAUGACUUGAGAGGCC | 121 | 1867 | 5.4458 | 91.8437 | 4.07596486** |
| miR4404 | ACAUGGAAGACUGGAUGGAUCAA | 16 | 245 | 0.7201 | 12.0523 | 4.06496741** |
| miR5490 | UAUGGAUUUGUAUUUGGAUGG | 63 | 921 | 2.8354 | 45.3069 | 3.9981066** |
| miR1869 | UGGAACAAUGUAGGCAAGGGAAGUA | 2119 | 28170 | 95.3695 | 1385.7726 | 3.86101877** |
| miR6218 | CGAAAAAUCACGAAGACUUGC | 30 | 393 | 1.3502 | 19.3329 | 3.83981303** |
| miR2916 | UGGGGGCUCGAAGACGAUCAGA | 6410 | 70604 | 288.4938 | 3473.237 | 3.58966864** |
| miR5656 | AGUGAGUGAGAGAUUGGGUGU | 21 | 213 | 0.9451 | 10.4782 | 3.47078011** |
| miR4371 | AUGGUGGUGACGGGUGACGGAGU | 1823 | 17547 | 82.0475 | 863.1932 | 3.39515222** |
| miR5999 | CUUCACGAUCAUGACGGACAA | 75 | 618 | 3.3755 | 30.4014 | 3.17096464** |
| miR3711 | GGCGCUAGAAGGAGGGCAUU | 109 | 784 | 4.9057 | 38.5675 | 2.97485472** |
| miR5813 | ACAGCAGGACGGUGGUCAUGGA | 3747 | 21623 | 168.6406 | 1063.7047 | 2.65707388** |
| miR7837 | UGGAUGGGAGGAUGUGGUGGU | 49 | 265 | 2.2053 | 13.0362 | 2.56347656** |
| miR7485 | AAAGACCAUCUUUGAUUCGUUUGA | 98 | 380 | 4.4107 | 18.6934 | 2.08344945** |
| miR1507 | UCUUCAUUCCACACGUCGUCU | 70 | 255 | 3.1505 | 12.5443 | 1.99337925** |
| miR4414 | AGCUGCUGACUCGUUGGUUC | 99 | 346 | 4.4557 | 17.0208 | 1.93357484** |
| miR5485 | UGACAAGUGGUAUCAGAGCAA | 152 | 496 | 6.841 | 24.3998 | 1.83459019** |
| miR5807 | AGGACUCUAGGAGGUGAUGUGGC | 319 | 1000 | 14.3572 | 49.1932 | 1.77668449** |
| miR398 | GGGGCGACAUGAGAUCACAUG | 8029 | 22159 | 361.3598 | 1090.0722 | 1.59291577** |
| miR1310 | AGAGGCAUCGGGGGCGCAACGC | 945 | 2373 | 42.5315 | 116.7355 | 1.45663972** |
| miR7532 | UGAACAGCCUCUGGUCGAUGGU | 3609 | 8670 | 162.4296 | 426.5051 | 1.39274843** |
| miR5298 | UUGGAGAUGAUAACGAAGAUGAC | 598 | 1379 | 26.9141 | 67.8374 | 1.33371869** |
| miR5262 | UCUGAAUCAGUAGACUCAUUU | 190 | 427 | 8.5513 | 21.0055 | 1.29655146** |
| miR5024 | AUAAGAACGGCCAAGAUACUAACA | 202 | 447 | 9.0914 | 21.9894 | 1.27423386** |
| miR5224 | CGGAAGACAUUGUCAGGAC | 10604 | 22940 | 477.2524 | 1128.4921 | 1.24157196** |
| miR1535 | CUUCUUUGUGGUAGAUUGUUU | 670 | 1399 | 30.1546 | 68.8213 | 1.19047705** |
| miR2938 | UGAUCUUCUGAGAAGGGUUCGAG | 216 | 433 | 9.7215 | 21.3007 | 1.13165** |
|  |  |  |  |  |  |  |
| ***Down-regulated miRNAs*** | |  |  |  |  |  |
| miR4351 | AUUGGGAGUGUGCAGUGGGAGUGG | 5736 | 0 | 258.1592 | 0.01 | -14.65597294** |
| miR5073 | GAUUGGUGAACGGCAGAAGUAUUU | 2978 | 0 | 134.0303 | 0.01 | -13.71027147** |
| miR1135 | UUCGCUCAAGUAAUUCUGACGGA | 2059 | 0 | 92.6691 | 0.01 | -13.17787312** |
| miR1516 | AAAGUUGGGACUUGAGGAGGCGGU | 709 | 0 | 31.9098 | 0.01 | -11.63978408** |
| miR5574 | UUUAAAUAGAACUCUAAAAGA | 657 | 0 | 29.5695 | 0.01 | -11.52989416** |
| miR4397 | UGUCUAAGAGUGUGGCGACAUUAC | 464 | 0 | 20.8832 | 0.01 | -11.02812713** |
| miR5525 | UCAAUCCUUGUGGAGACGAUCUGA | 418 | 0 | 18.8129 | 0.01 | -10.87750643** |
| miR6162 | UUUGAUACGACUGUCAUUUACGG | 395 | 0 | 17.7777 | 0.01 | -10.79585287** |
| miR6138 | CACGUUUGGGAUUGAGGUUGAAA | 372 | 0 | 16.7425 | 0.01 | -10.70929917** |
| miR6108 | UAUGGGUGAGAAGGGAAGAUA | 33936 | 19 | 1527.3518 | 0.9347 | -10.67424131** |
| miR5025 | AUCUGUAUAUAUGAGUAAUGAUCA | 337 | 0 | 15.1673 | 0.01 | -10.56674863** |
| miR4381 | UUGUGACGGUCAACUGGUGAAAU | 334 | 0 | 15.0323 | 0.01 | -10.55385004** |
| miR7130 | GUUUGGAAGUGUGGUGAGGUGGC | 328 | 0 | 14.7622 | 0.01 | -10.52769204** |
| miR7697 | UCCGGAUAAAUUAUGAUCGGAUA | 299 | 0 | 13.457 | 0.01 | -10.39414106** |
| miR6032 | UGGAGCAUGAAUCAGAAUCGG | 296 | 0 | 13.322 | 0.01 | -10.37959505** |
| miR1165 | UACUGUAGCAAGCGGCCAUCC | 288 | 0 | 12.962 | 0.01 | -10.34007259** |
| miR3509 | AUCUAACGACUGCUACAUAAUCAU | 278 | 0 | 12.5119 | 0.01 | -10.28908522** |
| miR5782 | UAGCUGUGUAGUAGAAGUUGAGA | 278 | 0 | 12.5119 | 0.01 | -10.28908522** |
| miR5074 | GCAAGGCCACCGUGCCGGCGACGC | 271 | 0 | 12.1969 | 0.01 | -10.25229873** |
| miR5516 | CUGCAGUUGCUGUCGGGUAGGCGG | 268 | 0 | 12.0618 | 0.01 | -10.23622943** |
| miR782 | ACAAACAGAGUUGGAUUUCUU | 249 | 0 | 11.2067 | 0.01 | -10.13014587** |
| miR5023 | UUGGUAGUGAUAAAGGCGC | 239 | 0 | 10.7566 | 0.01 | -10.0710064** |
| miR7708 | UGUCAUGAACUGAACGAAAGACAGC | 239 | 0 | 10.7566 | 0.01 | -10.0710064** |
| miR5780 | AAACUUAACUGACGGUAGGGA | 2925 | 3 | 131.645 | 0.1476 | -9.80074424** |
| miR2863 | AUAUAGGGACUAAAUGGGCAAA | 836 | 1 | 37.6257 | 0.0492 | -9.57884436** |
| miR6247 | UGGCUGAAUGAACAUAAGGCA | 829 | 1 | 37.3107 | 0.0492 | -9.56671537** |
| miR444 | UGCAUGUUAGUUGUGGCAAGCUU | 759 | 2 | 34.1602 | 0.0984 | -8.43944239** |
| miR779 | UCUGCUCAUAGAUUGUCUGCUCAU | 603 | 2 | 27.1391 | 0.0984 | -8.10749886** |
| miR5561 | CAUAAGAGAGAGAGCAUAGACAA | 457 | 2 | 20.5681 | 0.0984 | -7.70753451** |
| miR5259 | CAAGGGGUAUUUGGAUGGACA | 617 | 3 | 27.7692 | 0.1476 | -7.5556491** |
| miR6145 | CAUUGUUCACAUGUACGGCACUAU | 516 | 3 | 23.2235 | 0.1476 | -7.2977489** |
| miR7812 | AUGUUAGGAAAUUGAUGGGUG | 887 | 6 | 39.9211 | 0.2952 | -7.07931495** |
| miR6475 | UCUUGCGAGAGUAAAGAAUGA | 3233 | 23 | 145.5071 | 1.1314 | -7.00683666** |
| miR6248 | AUGUUGUAGGAAUGGAGGUAGGUA | 990 | 9 | 44.5568 | 0.4427 | -6.65317244** |
| miR5373 | UGUCUUGAUUUUAGAUGCAUG | 3543 | 47 | 159.4592 | 2.3121 | -6.10783973** |
| miR158 | UUCAAAACUGUUAGACAAAGC | 364 | 5 | 16.3825 | 0.246 | -6.05735341** |
| miR5789 | UGAUGAGCAUCUGGUCGGUAU | 289 | 4 | 13.007 | 0.1968 | -6.04641422** |
| miR5158 | UGAGACGGGAUGAGAUGAGAU | 465 | 7 | 20.9282 | 0.3444 | -5.92521928** |
| miR6425 | UUGCUUCCGUGGACAUAGGCA | 308 | 5 | 13.8621 | 0.246 | -5.81634371** |
| miR5721 | AGAAAAUGGUAGAGAGAAAGUGGA | 346 | 6 | 15.5724 | 0.2952 | -5.72115477** |
| miR1149 | CGGAUCAAUACUGACCCCCAGC | 392 | 7 | 17.6427 | 0.3444 | -5.67884241** |
| miR7712 | UUCAAUUGUAGAAACUUUAGAUGGC | 333 | 7 | 14.9873 | 0.3444 | -5.44351155** |
| miR5818 | UCGAACUGAGAGGCACAGGUU | 507 | 15 | 22.8185 | 0.7379 | -4.95063483** |
| miR5268 | ACAAGUGGAAUGAGAUGGAUGGUU | 337 | 10 | 15.1673 | 0.4919 | -4.94645542** |
| miR7767 | CCCAAGAUGAGUGCUCUCC | 322 | 14 | 14.4922 | 0.6887 | -4.39525714** |
| miR3437 | AAAAAUACAAGGACUAAACGGAU | 892 | 39 | 40.1461 | 1.9185 | -4.38720917** |
| miR5830 | AUGAGAGGAGGUGAUGUGACAUCA | 239 | 12 | 10.7566 | 0.5903 | -4.18762998** |
| miR5369 | UGAGAAAGGAGAGAUGGUGCA | 16576 | 868 | 746.0332 | 42.6997 | -4.126942** |
| miR5519 | UGGUAGACGCUACGGACUUAG | 1606 | 90 | 72.281 | 4.4274 | -4.02908484** |
| miR8143 | GGGAUGGUGGAAAGAUGAUGGUA | 423 | 26 | 19.0379 | 1.279 | -3.89578618** |
| miR5256 | AUAUAGGAUUAUGAAGAUUAA | 271 | 27 | 12.1969 | 1.3282 | -3.19897021** |
| miR5210 | UAGGAUGUGUUUGGAAUUGAGGUU | 377 | 42 | 16.9676 | 2.0661 | -3.03780053** |
| miR1222 | UUAAGAGAGGUCAGUGGUUCA | 421 | 52 | 18.9479 | 2.558 | -2.88894979** |
| miR5742 | CCAAAUCAGAUGGUCGCGGAU | 453 | 56 | 20.3881 | 2.7548 | -2.88770785** |
| miR164 | UGGAGAAGCAGGGCACGUGCA | 57341 | 9234 | 2580.7366 | 454.25 | -2.50622448** |
| miR5368 | AGGGACAGUCUCAGGUAGA | 589 | 103 | 26.509 | 5.0669 | -2.38730699** |
| miR3946 | GUGUAGAGAGAGAGAGAGAGACAC | 235 | 48 | 10.5766 | 2.3613 | -2.16322268** |
| miR5077 | UUCACGUCGGGUUCACCA | 5365 | 1293 | 241.4616 | 63.6068 | -1.92454086** |
| miR156 | UUGACAGAAGAGAGUGAGCAC | 475712 | 115344 | 21410.289 | 5674.141 | -1.91583036** |
| miR2118 | GUCGAUGGAACAAUGUAGGCAAGG | 32284 | 9127 | 1453.0005 | 448.9864 | -1.69429155** |
| miR1023 | AGCGAGAUUGGGAAAGUGCAU | 434 | 125 | 19.533 | 6.1492 | -1.66744291** |
| miR5380 | GAGAAUGAGAUGGGGAUGGGGAA | 329 | 98 | 14.8072 | 4.8209 | -1.61892445** |
| miR535 | UGACAAUGAGAGAGAGCACAC | 185022 | 57065 | 8327.2537 | 2807.2102 | -1.56870368** |
| miR5059 | UCGUUCCUGGGCAGCAACACCA | 10610 | 3334 | 477.5225 | 164.0101 | -1.54178405** |
| miR894 | GUUUCACGUCGGGUUCACCA | 28609 | 9269 | 1287.6004 | 455.9718 | -1.49766842** |
| miR171 | CGAGCCGAAUCAAUAUCACUC | 13357 | 4441 | 601.1562 | 218.467 | -1.46032453** |
| miR1048 | UGGAAGAUAGUGUAGAGAC | 298 | 104 | 13.412 | 5.1161 | -1.39040802* |
| miR6116 | UCAUUGUACACAAGCUGAG | 7142 | 2748 | 321.4388 | 135.1829 | -1.24963141** |
| miR157 | UUGACGGAAGAUAGAGAGCAC | 1935764 | 770693 | 87122.601 | 37912.8583 | -1.2003598** |
| miR403 | UUAGAUUCACGCACAAACUCG | 2596 | 1036 | 116.8377 | 50.9642 | -1.19694978** |
| miR858 | CUCGUUGUCUGUUCGACCUUG | 837 | 338 | 37.6707 | 16.6273 | -1.17988892** |
| miR1446 | CGAACUCUCUCCCUCAACGGC | 26042 | 10652 | 1172.0679 | 524.006 | -1.16140091** |
| miR5161 | UUUUGAUAGAGUGGAGUAUA | 22914 | 9463 | 1031.2865 | 465.5153 | -1.14754469** |
| miR8155 | CGUAACCUGGCUCUGAUACCA | 288 | 121 | 12.962 | 5.9524 | -1.12274496** |
| miR6478 | CCGACCUUAGCUCAGUUGGCAG | 2082 | 914 | 93.7042 | 44.9626 | -1.05938825** |
| miR530 | UGCAUUUGCACCUGCAUCUUG | 435 | 195 | 19.578 | 9.5927 | -1.02922455** |
| miR3951 | UAGAUAAAGAUGAGAGAAAAA | 18102 | 8139 | 814.7136 | 400.3835 | -1.02491047** |
| miR5054 | GUUCCCCACAGACGGCGCCA | 4885 | 2217 | 219.8584 | 109.0613 | -1.0114354** |
|  |  |  |  |  |  |  |
| ***Equally expressed miRNAs*** | |  |  |  |  |  |
| miR5822 | UGUCUGCGAGUCGGGUUG | 1244 | 2272 | 55.9885 | 111.767 | 0.99729185 |
| miR4413 | AAGAGAAUUGAAAGGACU | 201 | 358 | 9.0464 | 17.6112 | 0.96107752 |
| miR5293 | AGAUGUAGAAGGGAUGGAAGAAGA | 136 | 238 | 6.1209 | 11.708 | 0.93567895 |
| miR3627 | UCGCAGGGGAGAUGGGACUAAC | 7480 | 12241 | 336.6511 | 602.174 | 0.83892624 |
| miR160 | GCGUACGAGGAGCCAAGCAUA | 5880 | 9478 | 264.6402 | 466.2532 | 0.8170814 |
| miR5662 | AGAGUGUGAGCAAUUGGAGAGUG | 943 | 1518 | 42.4414 | 74.6753 | 0.81515888 |
| miR397 | UCAUUGAGUGCAGCGUUGAUG | 1816 | 2855 | 81.7324 | 140.4466 | 0.7810417 |
| miR6464 | UUGAUUGAUUGUUGGAUUUU | 5136 | 7505 | 231.1551 | 369.195 | 0.67552182 |
| miR5198 | GGGGUGAAGAGAUUGGGGA | 374 | 543 | 16.8326 | 26.7119 | 0.66622456 |
| miR5052 | CCCGUGGACGUAGGCAUA | 2517 | 3619 | 113.2822 | 178.0302 | 0.6522008 |
| miR5539 | AAGAAAACGGGAUGGCGAGCU | 191 | 272 | 8.5963 | 13.3806 | 0.63835507 |
| miR5207 | CAUUAAGGUGUUUGGACGUU | 1053 | 1332 | 47.3922 | 65.5253 | 0.46740242 |
| miR5492 | AGACUAGGAGAAACAGAUAUGGUU | 411 | 509 | 18.4978 | 25.0393 | 0.43684053 |
| miR4385 | AAUCGAUGUUGAAAAGUUUGAUGG | 201 | 243 | 9.0464 | 11.9539 | 0.40206569 |
| miR5149 | GAGCAGCUGGAAGAUUUGGGCA | 381 | 458 | 17.1476 | 22.5305 | 0.39387266 |
| miR6466 | UCAGUGGUAGAGCAUUUGACUGCA | 871 | 1046 | 39.2009 | 51.4561 | 0.39245534 |
| miR482 | AGUGGGAGCGUGGGGUAAGAAG | 3544 | 4249 | 159.5042 | 209.0219 | 0.39005969 |
| miR8124 | ACUUGGUACGUGGAGCGGU | 261 | 311 | 11.7468 | 15.2991 | 0.38117899 |
| miR5386 | CGUCGGCUGUCGGCGGACUG | 2074 | 2189 | 93.3442 | 107.6839 | 0.20617028 |
| miR774 | UGAGGAUGAAGAUGAGGAUGC | 495 | 516 | 22.2784 | 25.3837 | 0.18825675 |
| miR1861 | CUGAUCUUGAGGCAAGGAGCUGUUG | 779 | 786 | 35.0603 | 38.6659 | 0.14122345 |
| miR5227 | ACUGAAGAUGAAGACGAUGAUGAA | 1106 | 1110 | 49.7776 | 54.6045 | 0.13352318 |
| miR6485 | AGAAUGUAGAAGAGGUAA | 318 | 318 | 14.3122 | 15.6434 | 0.12830865 |
| miR5740 | UGGAACAGGAAACAACAUUUGG | 248 | 246 | 11.1617 | 12.1015 | 0.11662911 |
| miR6118 | UGGACGAUAUGGGUGGUUCGGAAA | 401 | 397 | 18.0477 | 19.5297 | 0.1138548 |
| miR472 | UUUUUCCCACACCUCCCAUCCC | 309 | 303 | 13.9071 | 14.9055 | 0.10002316 |
| miR6267 | UAGGAAUAGGUCAGGCAAUGU | 593 | 573 | 26.6891 | 28.1877 | 0.07881511 |
| miR1091 | CGGCAUGUGAGGGAAGAGUUG | 408 | 394 | 18.3628 | 19.3821 | 0.07793883 |
| miR5013 | UUUGUGACAGUCAGAGUGCCUU | 329 | 317 | 14.8072 | 15.5942 | 0.07471069 |
| miR5766 | UUGAGGCAUGUAGAAGGGCAA | 13886 | 13293 | 624.9648 | 653.9253 | 0.06535091 |
| miR5244 | UUAUCGGAUGAAGAUUGUUGG | 1670 | 1588 | 75.1614 | 78.1188 | 0.05567785 |
| miR408 | ACGGGGAACAGGCAGAGCAUG | 19871 | 18728 | 894.3307 | 921.2903 | 0.04284742 |
| miR2652 | UUAUGCAGGGUGGAUUGGAUU | 225 | 211 | 10.1265 | 10.3798 | 0.03564302 |
| miR6263 | AAUGGAAAAAGGGGAUGGA | 248 | 230 | 11.1617 | 11.3144 | 0.01960331 |
| miR479 | UGUGAUAUUGGUUCGGCUCAUC | 17953 | 8314 | 808.0076 | 408.9923 | -0.98229518 |
| miR7838 | AGCAUGUGCUGGGAGGAGAGAGA | 969 | 454 | 43.6116 | 22.3337 | -0.96548964 |
| miR5294 | GCUAGAAUCGGAAUGAGUUAGCAU | 223 | 105 | 10.0365 | 5.1653 | -0.9583322 |
| miR162 | UCGAUAAACCUCUGCAUCCAG | 1533 | 730 | 68.9955 | 35.911 | -0.94207644 |
| miR818 | AGGAGCAUUAGGAUGGACCAU | 1355 | 658 | 60.9843 | 32.3691 | -0.91382063 |
| miR2089 | UUACCUAUGCCACCCAUUCC | 1558 | 761 | 70.1206 | 37.436 | -0.90541205 |
| miR7502 | UUUUGAAAAGUAGAGAAUGAAUAA | 571 | 279 | 25.6989 | 13.7249 | -0.90491097 |
| miR167 | UGAAGCUGCCAGCAUGAUCUGA | 780656 | 384075 | 35134.852 | 18893.88 | -0.89498381 |
| miR169 | CAGCCAAGGAUGACUUGCCGG | 802 | 396 | 36.0955 | 19.4805 | -0.88978828 |
| miR5033 | GCUGGACAAAUAGCAAACUAC | 10030 | 5020 | 451.4185 | 246.9499 | -0.87024716 |
| miR1134 | CAGAACAAAGAAGAAGAAGAAGAU | 412 | 209 | 18.5428 | 10.2814 | -0.85082238 |
| miR1445 | UCCCUUGUAGAUCUAGUAGAAGAA | 4860 | 2477 | 218.7332 | 121.8516 | -0.84404702 |
| miR1887 | UAUUAUGUAGAAGUCAUAAGAGA | 285 | 146 | 12.8269 | 7.1822 | -0.83667481 |
| miR6432 | CCGACUUGGAGAAAAAGAUGG | 232 | 119 | 10.4416 | 5.854 | -0.83484815 |
| miR7760 | GGCUUUGGUCGGAGUGGUGGGCUG | 399 | 209 | 17.9577 | 10.2814 | -0.80456586 |
| miR8122 | UAAGGAAGGUUUUGUGGACAAG | 1649 | 865 | 74.2163 | 42.5521 | -0.80250575 |
| miR165 | UCGGACCAGGCUUCAUCCCCC | 945 | 501 | 42.5315 | 24.6458 | -0.78718993 |
| miR390 | AAGCUCAGGAGGGAUAGCGCC | 2734 | 1453 | 123.0487 | 71.4777 | -0.7836643 |
| miR5772 | UAGGAAUGUGAGUAGAGUAAGCAU | 315 | 174 | 14.1772 | 8.5596 | -0.72795734 |
| miR3699 | ACAGAAGAUAGACAUUUGAAC | 3181 | 1799 | 143.1667 | 88.4986 | -0.69396943 |
| miR6484 | UGAUGAGCUCUGCAAGAAUGG | 535 | 303 | 24.0787 | 14.9055 | -0.69191273 |
| miR7726 | UGUUGCAUGUCGGACGUCACGGU | 352 | 201 | 15.8424 | 9.8878 | -0.68006944 |
| miR3947 | AAUGAUUUAGUAGACGACGUUACA | 253 | 148 | 11.3867 | 7.2806 | -0.64522044 |
| miR5770 | UUCAGGAUAUGGUUUUGAUAA | 112329 | 66174 | 5055.5722 | 3255.3111 | -0.63507896 |
| miR393 | UCAUGCGAUCCCUUCGGAAUU | 1429 | 847 | 64.3148 | 41.6666 | -0.62625939 |
| miR5631 | UGACGAGGAAGAGAUAAUUUU | 463 | 273 | 20.8382 | 13.4297 | -0.63380359 |
| miR166 | UCGGACCAGGCUUCAUUCCCC | 524493 | 309678 | 23605.767 | 15234.0531 | -0.63183954 |
| miR393 | UCAUGCGAUCCCUUCGGAAUU | 1429 | 847 | 64.3148 | 41.6666 | -0.62625939 |
| miR3948 | GGAGUGGGAGUGGGAGUAGGUUGU | 7760 | 4651 | 349.253 | 228.7976 | -0.61020059 |
| miR6019 | AACAGGUGGACGGUUGUAAAUUUU | 2175 | 1305 | 97.8899 | 64.1971 | -0.60865189 |
| miR5254 | AGCGGUGGAAGCAAUUGUGUA | 35150 | 21140 | 1581.9901 | 1039.9443 | -0.60523431 |
| miR5139 | AACCUGGCUCUGAUACCA | 1531 | 926 | 68.9055 | 45.5529 | -0.59707624 |
| miR6260 | UGGAGUGGAGAAUGGGGUAGGCCU | 468 | 284 | 21.0632 | 13.9709 | -0.59229967 |
| miR7496 | AGACCAAAUUGUUAGACGAUGUGU | 1031 | 634 | 46.402 | 31.1885 | -0.57317282 |
| miR4393 | UUUGAAUAAGGGACACAGAGACC | 570 | 351 | 25.6539 | 17.2668 | -0.57117743 |
| miR5534 | CUUUAGACAACAGUAGAAUGG | 2507 | 1556 | 112.8321 | 76.5446 | -0.55980505 |
| miR6448 | UAGGGACACAAUUAACAAAUGGC | 270 | 168 | 12.1518 | 8.2645 | -0.55617059 |
| miR8138 | UAAAGAUGGGAACAAAACAA | 682 | 426 | 30.6947 | 20.9563 | -0.55060555 |
| miR4406 | ACCUUGUACUAGAGAACCGGUGUA | 1520 | 955 | 68.4104 | 46.9795 | -0.54218431 |
| miR1520 | GUCACGAUCCUGUUGGACUAA | 752 | 473 | 33.8451 | 23.2684 | -0.54057497 |
| miR1033 | AUAGACGGGUCGAUGAUGUGGCAU | 282 | 178 | 12.6919 | 8.7564 | -0.53549829 |
| miR1171 | AGUGUGGAGUGGGAGUGGGAGUGG | 6139 | 3970 | 276.2969 | 195.297 | -0.50054959 |
| miR473 | UGAGGCCGUUGGGGAGAGUGG | 474 | 307 | 21.3332 | 15.1023 | -0.49833211 |
| miR6262 | UCUUUAGACAACGGUAGAAUGGU | 16467 | 10751 | 741.1275 | 528.8761 | -0.48679198 |
| miR396 | UUCCACAGCUUUCUUGAACUG | 3868 | 2527 | 174.0864 | 124.3112 | -0.48584722 |
| miR3952 | UGAAGGGCCUUUCUAGAGCAC | 675 | 447 | 30.3796 | 21.9894 | -0.46629463 |
| miR8051 | GAUAGUAUGGUAGAAAGAUUCA | 295 | 196 | 13.277 | 9.6419 | -0.46153983 |
| miR6025 | UACCAACAAGAGAUGAACAUU | 250 | 167 | 11.2517 | 8.2153 | -0.45375783 |
| miR1858 | GUGAUGAGGAGGAGUGGGGUC | 338 | 227 | 15.2123 | 11.1669 | -0.44600956 |
| miR5234 | UUUUGUUAUGGAUGGCUGAAG | 889 | 598 | 40.0111 | 29.4175 | -0.44372565 |
| miR7696 | UUCAAAUGAGAACUUUGAAG | 7917 | 5345 | 356.3191 | 262.9377 | -0.43844881 |
| miR1857 | UGGAUUUUUUUGAAGCAUCGG | 359 | 243 | 16.1575 | 11.9539 | -0.43472261 |
| miR6195 | UGAGACGAGAUGGGAUGAG | 797 | 544 | 35.8704 | 26.7611 | -0.42265641 |
| miR159 | UUUGGAUUGAAGGGAGCUCUA | 5295 | 3647 | 238.3112 | 179.4076 | -0.40960575 |
| miR1863 | AGAGUUUGUGGCUGUAUCAUUACU | 1210 | 834 | 54.4583 | 41.0271 | -0.40857477 |
| miR3633 | UUACCUAUGCCACCCAUUCCUU | 3141 | 2197 | 141.3665 | 108.0775 | -0.38737407 |
| miR4403 | ACGACACGAACACGACCCGAUGAC | 1400 | 985 | 63.0096 | 48.4553 | -0.37891718 |
| miR172 | AGAAUCUUGAUGAUGCUGCAU | 74690 | 52745 | 3361.5601 | 2594.6956 | -0.37356565 |
| miR1159 | ACAAUGACAUAGAUGACGGAU | 4032 | 2857 | 181.4675 | 140.545 | -0.36867906 |
| miR5524 | GAAAAAUGUGGAUUCAUGACGG | 558 | 396 | 25.1138 | 19.4805 | -0.36644963 |
| miR6171 | AUUGUGGACGGCUGAAGGUUU | 1687 | 1201 | 75.9265 | 59.081 | -0.36190926 |
| miR850 | UAAAGAUCCGGAAACAACCAAG | 866 | 617 | 38.9759 | 30.3522 | -0.36078125 |
| miR6029 | UGGGUUGUAAUUUGAUGGCUU | 823 | 592 | 37.0406 | 29.1224 | -0.34697821 |
| miR5269 | AGAAGAUGGUGGGACAACUUGCUU | 759 | 556 | 34.1602 | 27.3514 | -0.32070174 |
| miR6300 | GUCGUUGUAGUAUAGUGGUG | 2639 | 1940 | 118.773 | 95.4348 | -0.31561957 |
| miR168 | UCGCUUGGUGCAGGUCGGGAA | 55988 | 41638 | 2519.8424 | 2048.3066 | -0.29890182 |
| miR1850 | UGGAAAGUAGAAGAGAUUGGG | 2115 | 1581 | 95.1894 | 77.7745 | -0.29150371 |
| miR477 | ACCUCCCUCGAAGGCUUCCAA | 772 | 579 | 34.7453 | 28.4829 | -0.2867218 |
| miR1515 | UCAUUUUUGCGUGCAAUGAUCC | 324 | 247 | 14.5822 | 12.1507 | -0.26316896 |
| miR3515 | AAAUGUAGAGCAAAAUGAAGGUAU | 354 | 270 | 15.9324 | 13.2822 | -0.26246948 |
| miR5719 | UUGUGAUGAAAAUAGACGUCC | 3258 | 2532 | 146.6323 | 124.5572 | -0.23539452 |
| miR3950 | UAUUUUCUGCAACAUGAUUGU | 2460 | 1917 | 110.7168 | 94.3034 | -0.23149246 |
| miR861 | CUUGGAGAAAUUAUGAGCGUCAGA | 1066 | 831 | 47.9773 | 40.8796 | -0.23097089 |
| miR8034 | UAUGACAGAAGAUCUUCAAAAACU | 298 | 233 | 13.412 | 11.462 | -0.22666559 |
| miR5657 | UGGACAAGGAAGAUUGAGGUG | 3716 | 2933 | 167.2454 | 144.2837 | -0.21305821 |
| miR440 | AUGUCACUGAUGAUCGAAGGACAA | 441 | 351 | 19.848 | 17.2668 | -0.2009929 |
| miR3954 | UUGGACAGAGAAAUCACGGUCA | 116108 | 92662 | 5225.653 | 4558.3407 | -0.19710257 |
| miR2636 | UUUGUGUUGAAGAUGGCUGAAUAU | 1364 | 1089 | 61.3893 | 53.5714 | -0.19652422 |
| miR6295 | AGGACAGGAGAUGAUUCAUGA | 1373 | 1098 | 61.7944 | 54.0141 | -0.19414004 |
| miR5250 | UGAUGCAUGUUGAUACGGAUC | 603 | 483 | 27.1391 | 23.7603 | -0.19181983 |
| miR7773 | UUAUUCCAUUCGUCGACACGU | 351 | 283 | 15.7974 | 13.9217 | -0.18235174 |
| miR3630 | GAACAAGUGAUGGAUAGACCUGCA | 286 | 231 | 12.872 | 11.3636 | -0.17981628 |
| miR7129 | AGAAAUCUAGAGAUCGUGUAU | 883 | 715 | 39.741 | 35.1731 | -0.17615568 |
| miR952 | ACAGAACAUGGCAUUGGUCCG | 1497 | 1219 | 67.3752 | 59.9665 | -0.16806088 |
| miR5376 | UGAGAGGGUUUGAAGAAUUUGGGC | 560 | 457 | 25.2038 | 22.4813 | -0.1649158 |
| miR7122 | UUGGACAGAGAAAUCACGGUCG | 1476 | 1224 | 66.4301 | 60.2125 | -0.14177407 |
| miR5062 | UGAACCUCUGGAGAAGAAGCCCC | 469 | 393 | 21.1082 | 19.3329 | -0.1267455 |
| miR5713 | UAUGAGCUUCAGAAGAACUUUGUU | 244 | 207 | 10.9817 | 10.183 | -0.10893875 |
| miR6140 | AAGUUUGUAGAAGAGUUUGUGGCU | 655 | 557 | 29.4795 | 27.4006 | -0.10550457 |
| miR8135 | AGAAUUUUGCAGUGGUUGGAU | 1263 | 1085 | 56.8436 | 53.3746 | -0.09084457 |
| miR5565 | UUUUGUUGGAAGAUUGUCGGA | 287 | 247 | 12.917 | 12.1507 | -0.08823161 |
| miR1078 | CUUGAUUGAUUGUUGGAU | 1431 | 1236 | 64.4048 | 60.8028 | -0.08303045 |
| miR7984 | UCCGACUUUGUGAAAUGACUU | 429 | 380 | 19.3079 | 18.6934 | -0.04666227 |
| miR3446 | CUGGAAGCAACUGUGGCACGG | 14904 | 13204 | 670.7818 | 649.5471 | -0.0464094 |
| miR8148 | UAGACGGAUCGAUGACGUGGCAU | 1508 | 1369 | 67.8703 | 67.3455 | -0.01119884 |

** and ** indicate a significant difference at P < 0.05 and P < 0.01, respectively.*

**Table S5 | List of novel miRNAs in *Citrus sinensis* leaves.**

| miRNA | *Sequence* | Expressed | | Normalized read count | | Fold change |
| --- | --- | --- | --- | --- | --- | --- |
|  |  | Control | Mg-deficiency | Control | Mg-deficiency |  |
| ***Up-regulated miRNAs*** | |  |  |  |  |  |
| novel_mir_96 | GTTCTCAGGTCGCCCCTGTGGGA | 0 | 11218 | 0.01 | 551.8494 | 15.75198699** |
| novel_mir_64 | TTTTGTTGCATGATGCTGATAA | 0 | 285 | 0.01 | 14.0201 | 10.45328092** |
| novel_mir_150 | ACTTGCTGTATCGGTCGACA | 0 | 185 | 0.01 | 9.1007 | 9.82983371** |
| novel_mir_88 | TTCAGAGTAGACAAATGGTAA | 0 | 170 | 0.01 | 8.3628 | 9.70784225** |
| novel_mir_147 | ACTGGTACTGTAACGGAGGAGGA | 0 | 118 | 0.01 | 5.8048 | 9.18110255** |
| novel_mir_45 | TGCTGAGGAACAGATGCAGTTG | 0 | 111 | 0.01 | 5.4604 | 9.09286283** |
| novel_mir_81 | TATGTTGCAACTGTGGTACGGTA | 0 | 92 | 0.01 | 4.5258 | 8.82202902** |
| novel_mir_23 | ACTCTCCCTCAAGGGCTTCTGA | 0 | 84 | 0.01 | 4.1322 | 8.69076627** |
| novel_mir_181 | CAAGCGAGACTCGAGCAAGTTG | 0 | 80 | 0.01 | 3.9355 | 8.62040313** |
| novel_mir_190 | TTTAATTGGTACGTGGCGTTGT | 0 | 72 | 0.01 | 3.5419 | 8.46837967** |
| novel_mir_146 | TTGGGGGTAGATTGAGGTTTA | 0 | 62 | 0.01 | 3.05 | 8.25266543** |
| novel_mir_26 | CCAGCGCTGCACTCGATCATG | 0 | 59 | 0.01 | 2.9024 | 8.18110255** |
| novel_mir_163 | ACGTGGCAGCATATCAGTGGACA | 0 | 53 | 0.01 | 2.6072 | 8.02635745** |
| novel_mir_122 | ATCGAGATTTTAGTTAGATGG | 0 | 48 | 0.01 | 2.3613 | 7.88343754** |
| novel_mir_144 | TTCAACTTTGAAAACGTCATC | 0 | 48 | 0.01 | 2.3613 | 7.88343754** |
| novel_mir_167 | ATGGAATAGCAGAATTGACAAGT | 0 | 42 | 0.01 | 2.0661 | 7.69076627** |
| novel_mir_176 | TCTCATCGGTTTGGATGGCATT | 0 | 36 | 0.01 | 1.771 | 7.4684204** |
| novel_mir_90 | GAGGGAAGTGGTTCGAGCCTC | 0 | 35 | 0.01 | 1.7218 | 7.42777376** |
| novel_mir_164 | TGCTAGCGGCAAACCATGACAC | 0 | 33 | 0.01 | 1.6234 | 7.34287471** |
| novel_mir_48 | TCCAAAGGGATCGCATTGATC | 0 | 33 | 0.01 | 1.6234 | 7.34287471** |
| novel_mir_49 | CAACTGTGGAATAGAAGTTGGGA | 0 | 31 | 0.01 | 1.525 | 7.25266543** |
| novel_mir_61 | CGCTATCCATCCTGAGTTTCA | 0 | 31 | 0.01 | 1.525 | 7.25266543** |
| novel_mir_161 | AATGGCTGTGGTGGGTGGTCT | 0 | 30 | 0.01 | 1.4758 | 7.20535341** |
| novel_mir_7 | TTATTGAGTGTGTATGTTACA | 0 | 29 | 0.01 | 1.4266 | 7.15643707** |
| novel_mir_179 | TCTCTTTCTGAAGATCCTGGAT | 0 | 25 | 0.01 | 1.2298 | 6.9422799** |
| novel_mir_24 | TTGACAGTGGGAAGGGCTAATT | 0 | 22 | 0.01 | 1.0823 | 6.75795664** |
| novel_mir_65 | TGTGAGAGGCATGTATCTTAC | 0 | 22 | 0.01 | 1.0823 | 6.75795664** |
| novel_mir_35 | TGCAGGTGAGATGATACCGTCA | 9 | 26 | 0.4051 | 1.279 | 1.65866627** |
| novel_mir_117 | AGATCATCTGGCAGTTTCACC | 167 | 378 | 7.5161 | 18.595 | 1.30685858** |
| novel_mir_104 | AAGGCTTTCAAGAAGGACCCA | 11 | 21 | 0.4951 | 1.0331 | 1.06118805* |
| novel_mir_50 | TTGTGGTAGATTGTTTGCTTA | 530 | 977 | 23.8536 | 48.0618 | 1.01068367** |
|  |  |  |  |  |  |  |
| ***Down-regulated miRNAs*** | |  |  |  |  |  |
| novel_mir_243 | TCCCTACTCCACCCATGCCATA | 1926 | 0 | 86.6832 | 0.01 | -13.08153679** |
| novel_mir_252 | TTGAGCCGCGTCAATATCTCC | 579 | 0 | 26.059 | 0.01 | -11.34756593** |
| novel_mir_250 | TAGATAACGGATTAACGGCTA | 368 | 0 | 16.5625 | 0.01 | -10.6937047** |
| novel_mir_233 | AGCAGGAAAGTGGCTGGTTGA | 309 | 0 | 13.9071 | 0.01 | -10.44160584** |
| novel_mir_251 | GGTGCAGCTGTGGTATGGTAC | 131 | 0 | 5.8959 | 0.01 | -9.20356824** |
| novel_mir_207 | TGAGGTTCTTGGGGAGAGTAG | 117 | 0 | 5.2658 | 0.01 | -9.0405089** |
| novel_mir_218 | TCCAAAGGGATCGCATTGAT | 111 | 0 | 4.9958 | 0.01 | -8.96457192** |
| novel_mir_297 | TGCCTGGCTCCCTGTATGCTT | 87 | 0 | 3.9156 | 0.01 | -8.61308959** |
| novel_mir_314 | TCCGACAATGTGGTAAACGTGTT | 83 | 0 | 3.7356 | 0.01 | -8.54519619** |
| novel_mir_202 | TATGTTGCAACAGTGGTACGGTA | 79 | 0 | 3.5555 | 0.01 | -8.47390867** |
| novel_mir_295 | TTGATGAAAATCTAACGGCTG | 73 | 0 | 3.2855 | 0.01 | -8.35996911** |
| novel_mir_317 | TTATTGGCTTTAGAAACAGGTT | 72 | 0 | 3.2405 | 0.01 | -8.34007264** |
| novel_mir_210 | CTAGACACAAGTGAGAGCGCA | 59 | 0 | 2.6554 | 0.01 | -8.05278538** |
| novel_mir_273 | TTTGATATGTGGTTAGCTTGG | 47 | 0 | 2.1153 | 0.01 | -7.72471848** |
| novel_mir_271 | TGCCTGGCTCCCTGTATGCCA | 46 | 0 | 2.0703 | 0.01 | -7.69369604** |
| novel_mir_321 | CTGAAAAGGAATGCTGGTCAA | 45 | 0 | 2.0253 | 0.01 | -7.66199182** |
| novel_mir_209 | ACCAGCGCTGCACTCGATCAT | 38 | 0 | 1.7103 | 0.01 | -7.41810561** |
| novel_mir_276 | AGGCAGTCTCCTTGGCTAAG | 37 | 0 | 1.6653 | 0.01 | -7.3796383** |
| novel_mir_244 | TCCCGTGAACGTAGGTATATT | 36 | 0 | 1.6202 | 0.01 | -7.34002811** |
| novel_mir_224 | TTTATGCGGATATATTTTTAA | 31 | 0 | 1.3952 | 0.01 | -7.12432813** |
| novel_mir_237 | AGGGATCAGGCTACGGTGCAA | 28 | 0 | 1.2602 | 0.01 | -6.9775089** |
| novel_mir_267 | TTAGAACCGAACCGAACCGAA | 28 | 0 | 1.2602 | 0.01 | -6.9775089** |
| novel_mir_215 | TCATACAAGTGAACATGATTT | 25 | 0 | 1.1252 | 0.01 | -6.81403765** |
| novel_mir_232 | ATACTGTATTGTATTATGTTG | 25 | 0 | 1.1252 | 0.01 | -6.81403765** |
| novel_mir_266 | TACGTTACAGATTCTGTATGT | 25 | 0 | 1.1252 | 0.01 | -6.81403765** |
| novel_mir_258 | TATGGTGGTCGCCGGCATGTG | 24 | 0 | 1.0802 | 0.01 | -6.75515464** |
| novel_mir_280 | GGAATGGCGGCTGGTTCAAAG | 24 | 0 | 1.0802 | 0.01 | -6.75515464** |
| novel_mir_319 | TTGGAGAGCAAGTAGGGCATT | 24 | 0 | 1.0802 | 0.01 | -6.75515464** |
| novel_mir_132 | GTGACAGAAGATAGAGAGCGC | 24996 | 2801 | 1124.991 | 137.7902 | -3.02936789** |
| novel_mir_43 | AGAAGAACGAGAGAAAGACGA | 142 | 24 | 6.391 | 1.1806 | -2.43652144** |
| novel_mir_188 | TACAAACTGACGTGGCATGAT | 5479 | 1227 | 246.5924 | 60.3601 | -2.03046124** |
| novel_mir_5 | TGGAGAAGGGGAGCGCGTGCA | 55 | 14 | 2.4754 | 0.6887 | -1.84571409** |
| novel_mir_112 | ATATTATAGAGTTCTAACTCAAA | 27 | 7 | 1.2152 | 0.3444 | -1.81903673** |
| novel_mir_189 | TAGATACAACTGTGGTACGGT | 76 | 21 | 3.4205 | 1.0331 | -1.72722732** |
| novel_mir_153 | AATTGTTGGGGAAAAATTAGGTT | 43 | 12 | 1.9353 | 0.5903 | -1.71303698** |
| novel_mir_71 | TTGACATGTGCACAGTCGGAC | 297 | 84 | 13.367 | 4.1322 | -1.69369372** |
| novel_mir_59 | CTTTCAGCAGCCTCCGGCGTC | 48 | 14 | 2.1603 | 0.6887 | -1.64928409** |
| novel_mir_63 | TGTGGAATCTGCAAGCTCGAG | 58 | 18 | 2.6104 | 0.8855 | -1.55970668** |
| novel_mir_12 | TACTAGGGATCTCAACATTTGGA | 213 | 76 | 9.5865 | 3.7387 | -1.35846748** |
| novel_mir_97 | TTGAGCCGCGCCAATATCACG | 38 | 14 | 1.7103 | 0.6887 | -1.31230183** |
| novel_mir_70 | AGGTGCAGCTGTATATGCAGG | 41 | 16 | 1.8453 | 0.7871 | -1.22923654** |
| novel_mir_2 | TAGAAGTTCCAACGGGTCGGG | 39 | 16 | 1.7553 | 0.7871 | -1.15709878** |
| novel_mir_11 | ATGGGGAGTAGCTGCGCGGTG | 2651 | 1097 | 119.3131 | 53.9649 | -1.1446592** |
| novel_mir_177 | GCTCAAGAATGCCGTGGGAAA | 364 | 158 | 16.3825 | 7.7725 | -1.07570491** |
| novel_mir_174 | GTGCTCTCTACCATTGTCATA | 141 | 62 | 6.346 | 3.05 | -1.05703828** |
| novel_mir_85 | TGTTTGTTGAAAAGTTGGCTT | 67 | 30 | 3.0155 | 1.4758 | -1.03090001** |
| novel_mir_20 | GGTCATGGGAGGATTGGCGAGA | 61380 | 28066 | 2762.519 | 1380.6565 | -1.00063006** |
|  |  |  |  |  |  |  |
| ***Equally expressed miRNAs*** | |  |  |  |  |  |
| novel_mir_127 | AGGGACAAGCTAAAAGACCAA | 690 | 1254 | 31.0547 | 61.6883 | 0.99018526 |
| novel_mir_101 | GATGCCTCATCTCGGACACC | 168 | 297 | 7.5611 | 14.6104 | 0.95032764 |
| novel_mir_39 | TTGTCGCAGGAGCGGTGGCACC | 15 | 25 | 0.6751 | 1.2298 | 0.86525059 |
| novel_mir_173 | TCAAGGAGCGCACGAACGGTT | 383 | 627 | 17.2376 | 30.8441 | 0.83943563 |
| novel_mir_40 | TTGTCGCCGGAGAGATAGCACC | 34 | 53 | 1.5302 | 2.6072 | 0.76878103 |
| novel_mir_9 | TGATTGAGCCGTGCCAATATC | 38 | 59 | 1.7103 | 2.9024 | 0.76299695 |
| novel_mir_99 | TTCCACCAAAGCATTCATTTCC | 87 | 125 | 3.9156 | 6.1492 | 0.65116534 |
| novel_mir_121 | CTGACAGCGGCTGTACTGTAGT | 2403 | 3260 | 108.1514 | 160.3698 | 0.56835015 |
| novel_mir_3 | GGAATGTTGTCTGGCTCGAGG | 480 | 587 | 21.6033 | 28.8764 | 0.41863919 |
| novel_mir_180 | CTGATGAGAGAGCGAATGATA | 343 | 419 | 15.4373 | 20.612 | 0.41706405 |
| novel_mir_83 | TCTGGATGAATATTTGAAGGC | 113 | 137 | 5.0858 | 6.7395 | 0.40616683 |
| novel_mir_58 | TGTTGGAACGGCTCAATCAAA | 1431 | 1673 | 64.4048 | 82.3002 | 0.35372772 |
| novel_mir_142 | AGCAAGCATCCTGGGCTAAT | 326 | 369 | 14.6722 | 18.1523 | 0.30706715 |
| novel_mir_78 | TTGAGAAGTGTAGTATTATTT | 215 | 229 | 9.6765 | 11.2652 | 0.2193157 |
| novel_mir_19 | TTCCCTAGTCCCCCTATTCCTA | 168 | 163 | 7.5611 | 8.0185 | 0.08473625 |
| novel_mir_141 | TCTGTATAGGACAGTTGATGTT | 330 | 162 | 14.8523 | 7.9693 | -0.89816145 |
| novel_mir_10 | TAATATAGGAATAAATTGGACA | 463 | 229 | 20.8382 | 11.2652 | -0.88735774 |
| novel_mir_155 | TACTAGACACAAGACATGGCAT | 28 | 14 | 1.2602 | 0.6887 | -0.87170513 |
| novel_mir_69 | AGGTGCAGTTGCAAGTGCAGA | 125 | 63 | 5.6259 | 3.0992 | -0.86018805 |
| novel_mir_80 | GGAAACCCTAGGGGGAGGTCG | 560 | 297 | 25.2038 | 14.6104 | -0.78664559 |
| novel_mir_135 | GCAATGCTCTTGAAGGACTAC | 52956 | 28435 | 2383.382 | 1398.8088 | -0.76881124 |
| novel_mir_15 | TATTTGGAGAGTATTTTGGAG | 27 | 15 | 1.2152 | 0.7379 | -0.71969655 |
| novel_mir_25 | TGGCGCAGCTGTCCTAAACGG | 72 | 41 | 3.2405 | 2.0169 | -0.68407688 |
| novel_mir_1 | TAGTGGTAGACTTCCCAAAAT | 61 | 35 | 2.7454 | 1.7218 | -0.67309879 |
| novel_mir_116 | TATGTTGCAACTGTGGTATGGTA | 153 | 90 | 6.886 | 4.4274 | -0.63720646 |
| novel_mir_74 | AGGGATAGTTATAGATGGAGGTT | 41 | 26 | 1.8453 | 1.279 | -0.52883912 |
| novel_mir_128 | CTGGAGACAACTGTGGTACGG | 444 | 293 | 19.983 | 14.4136 | -0.47134247 |
| novel_mir_41 | GTAGATGATGAGTATGCACTG | 306 | 217 | 13.7721 | 10.6749 | -0.36752601 |
| novel_mir_134 | AAGTCATTAGAAGAACTGCCG | 27153 | 20219 | 1222.07 | 994.6374 | -0.29708481 |
| novel_mir_187 | TGAGGGAAGAGCTTAGAAGG | 1810 | 1451 | 81.4624 | 71.3793 | -0.19062857 |
| novel_mir_6 | ATACAGAGTCTGTAACGTAGT | 46 | 37 | 2.0703 | 1.8201 | -0.18582212 |
| novel_mir_184 | AGTAAGATTGTCGTCACACAT | 4703 | 3927 | 211.6671 | 193.1817 | -0.13183861 |
| novel_mir_27 | AGATCATGCGGCAGTTTCACC | 373 | 319 | 16.7875 | 15.6926 | -0.097303 |
| novel_mir_56 | TGGAGGCAGCGGTTCATCGATC | 116 | 100 | 5.2208 | 4.9193 | -0.08581785 |
| novel_mir_162 | TAATCGTGGGAGACGAAGCTG | 19350 | 17241 | 870.8822 | 848.14 | -0.03817516 |

** and ** indicate a significant difference at P < 0.05 and P < 0.01, respectively.*

**Table S6 | List of novel miRNAs in *Citrus sinensis* leaves after removing these miRNAs with normalized read-count less than 10 TPM in the two miRNA libraries constructed from Mg-sufficient and -deficient leaves**

| **miRNA** | ***Sequence*** | **Expressed** | | **Normalized read count** | | **Fold change** |
| --- | --- | --- | --- | --- | --- | --- |
|  |  | **Control** | **Mg-deficiency** | **Control** | **Mg-deficiency** |  |
| ***Up-regulated miRNAs*** | |  |  |  |  |  |
| novel_mir_96 | GTTCTCAGGTCGCCCCTGTGGGA | 0 | 11218 | 0.01 | 551.8494 | 15.75198699** |
| novel_mir_64 | TTTTGTTGCATGATGCTGATAA | 0 | 285 | 0.01 | 14.0201 | 10.45328092** |
| novel_mir_117 | AGATCATCTGGCAGTTTCACC | 167 | 378 | 7.5161 | 18.595 | 1.30685858** |
| novel_mir_50 | TTGTGGTAGATTGTTTGCTTA | 530 | 977 | 23.8536 | 48.0618 | 1.01068367** |
|  |  |  |  |  |  |  |
| ***Down-regulated miRNAs*** | |  |  |  |  |  |
| novel_mir_252 | TCCCTACTCCACCCATGCCATA | 1926 | 0 | 86.6832 | 0.01 | -13.08153679** |
| novel_mir_252 | TTGAGCCGCGTCAATATCTCC | 579 | 0 | 26.059 | 0.01 | -11.34756593** |
| novel_mir_250 | TAGATAACGGATTAACGGCTA | 368 | 0 | 16.5625 | 0.01 | -10.6937047** |
| novel_mir_233 | AGCAGGAAAGTGGCTGGTTGA | 309 | 0 | 13.9071 | 0.01 | -10.44160584** |
| novel_mir_132 | GTGACAGAAGATAGAGAGCGC | 24996 | 2801 | 1124.9907 | 137.7902 | -3.02936789** |
| novel_mir_188 | TACAAACTGACGTGGCATGAT | 5479 | 1227 | 246.5924 | 60.3601 | -2.03046124** |
| novel_mir_71 | TTGACATGTGCACAGTCGGAC | 297 | 84 | 13.367 | 4.1322 | -1.69369372** |
| novel_mir_11 | ATGGGGAGTAGCTGCGCGGTG | 2651 | 1097 | 119.3131 | 53.9649 | -1.1446592** |
| novel_mir_177 | GCTCAAGAATGCCGTGGGAAA | 364 | 158 | 16.3825 | 7.7725 | -1.07570491** |
| novel_mir_20 | GGTCATGGGAGGATTGGCGAGA | 61380 | 28066 | 2762.5192 | 1380.6565 | -1.00063006** |
|  |  |  |  |  |  |  |
| ***Equally expressed miRNAs*** | |  |  |  |  |  |
| novel_mir_127 | AGGGACAAGCTAAAAGACCAA | 690 | 1254 | 31.0547 | 61.6883 | 0.99018526 |
| novel_mir_101 | GATGCCTCATCTCGGACACC | 168 | 297 | 7.5611 | 14.6104 | 0.95032764 |
| novel_mir_173 | TCAAGGAGCGCACGAACGGTT | 383 | 627 | 17.2376 | 30.8441 | 0.83943563 |
| novel_mir_121 | CTGACAGCGGCTGTACTGTAGT | 2403 | 3260 | 108.1514 | 160.3698 | 0.56835015 |
| novel_mir_3 | GGAATGTTGTCTGGCTCGAGG | 480 | 587 | 21.6033 | 28.8764 | 0.41863919 |
| novel_mir_180 | CTGATGAGAGAGCGAATGATA | 343 | 419 | 15.4373 | 20.612 | 0.41706405 |
| novel_mir_58 | TGTTGGAACGGCTCAATCAAA | 1431 | 1673 | 64.4048 | 82.3002 | 0.35372772 |
| novel_mir_142 | AGCAAGCATCCTGGGCTAAT | 326 | 369 | 14.6722 | 18.1523 | 0.30706715 |
| novel_mir_78 | TTGAGAAGTGTAGTATTATTT | 215 | 229 | 9.6765 | 11.2652 | 0.2193157 |
| novel_mir_141 | TCTGTATAGGACAGTTGATGTT | 330 | 162 | 14.8523 | 7.9693 | -0.89816145 |
| novel_mir_10 | TAATATAGGAATAAATTGGACA | 463 | 229 | 20.8382 | 11.2652 | -0.88735774 |
| novel_mir_80 | GGAAACCCTAGGGGGAGGTCG | 560 | 297 | 25.2038 | 14.6104 | -0.78664559 |
| novel_mir_135 | GCAATGCTCTTGAAGGACTAC | 52956 | 28435 | 2383.3817 | 1398.8088 | -0.76881124 |
| novel_mir_128 | CTGGAGACAACTGTGGTACGG | 444 | 293 | 19.983 | 14.4136 | -0.47134247 |
| novel_mir_41 | GTAGATGATGAGTATGCACTG | 306 | 217 | 13.7721 | 10.6749 | -0.36752601 |
| novel_mir_134 | AAGTCATTAGAAGAACTGCCG | 27153 | 20219 | 1222.0704 | 994.6374 | -0.29708481 |
| novel_mir_187 | TGAGGGAAGAGCTTAGAAGG | 1810 | 1451 | 81.4624 | 71.3793 | -0.19062857 |
| novel_mir_184 | AGTAAGATTGTCGTCACACAT | 4703 | 3927 | 211.6671 | 193.1817 | -0.13183861 |
| novel_mir_27 | AGATCATGCGGCAGTTTCACC | 373 | 319 | 16.7875 | 15.6926 | -0.097303 |
| novel_mir_162 | TAATCGTGGGAGACGAAGCTG | 19350 | 17241 | 870.8822 | 848.14 | -0.03817516 |

*** indicates a significant difference at P < 0.05 and P < 0.01.*

**Table S7 | List of Mg-deficiency-responsive known miRNAs in *Citrus sinensis* leaves.**

| **miRNA** | ***Sequence*** | | **Expressed** | | **Normalized read count** | | **Fold change** |
| --- | --- | --- | --- | --- | --- | --- | --- |
|  |  |  | **Control** | **Mg-deficiency** | **Control** | **Mg-deficiency** |  |
| ***Up-regulated miRNAs*** | | |  |  |  |  |  |
| miR5832 | UUGGCGGAGCGAUUUGUC | | 0 | 40754 | 0.01 | 2004.8199 | 17.61311311** |
| miR5493 | ACCGGGCUCGAGCGACGCGUG | | 0 | 25261 | 0.01 | 1242.6695 | 16.92308312** |
| miR1104 | CGCAGCUGUUCGUCUUUCCC | | 0 | 4433 | 0.01 | 218.0735 | 14.41252685** |
| miR5760 | UGUUUAAGAUAGUUUGUAAGGAU | | 0 | 3899 | 0.01 | 191.8043 | 14.22734744** |
| miR3443 | GUGCGUUUGAGAUUGAGGUU | | 0 | 2668 | 0.01 | 131.2475 | 13.68000232** |
| miR5666 | AGGGACAUAGAGACAUUUACU | | 0 | 2555 | 0.01 | 125.6886 | 13.61756618** |
| miR7730 | AUGAACACGGCACGAUUGAAGUUAU | | 0 | 1366 | 0.01 | 67.1979 | 12.71420043** |
| miR6166 | AUUAGAUAACGGAUUAAUGGA | | 0 | 1131 | 0.01 | 55.6375 | 12.44184188** |
| miR6233 | UCAAGUUGUUUUGGAAUUACUG | | 0 | 1045 | 0.01 | 51.4069 | 12.3277463** |
| miR4408 | AACAAAUAUGGAUGAUUGUUGGA | | 0 | 1044 | 0.01 | 51.3577 | 12.32636488** |
| miR8039 | UUUCCUACUGAGAUUAUCAAC | | 0 | 956 | 0.01 | 47.0287 | 12.19932574** |
| miR5160 | CAGAAGAUCGAUGGUAAUUUCUU | | 0 | 944 | 0.01 | 46.4384 | 12.18110255** |
| miR1851 | AUAUGGGAUGGCAUUUGGC | | 0 | 840 | 0.01 | 41.3223 | 12.01270484** |
| miR917 | UUUGGACGGUUAUUUUCGAA | | 0 | 814 | 0.01 | 40.0433 | 11.96734516** |
| miR1077 | UUGAAGUGUUCGGAUCGCGGC | | 22 | 74084 | 0.9902 | 3644.4294 | 11.84568538** |
| miR4223 | AUGGUUUUGAUAAAUAGAACAGGC | | 0 | 578 | 0.01 | 28.4337 | 11.47338613** |
| miR1172 | UAGACGGAGACAUAGUGAA | | 0 | 432 | 0.01 | 21.2515 | 11.05334896** |
| miR5821 | UGCGGAGGGAUGGUGGGAG | | 0 | 379 | 0.01 | 18.6442 | 10.86451118** |
| miR1162 | CGGCUUAAUUUGACUCAACACG | | 0 | 359 | 0.01 | 17.6604 | 10.7863023** |
| miR6449 | CUAUGAUUCUGGAAAUAAACGGUU | | 0 | 272 | 0.01 | 13.3806 | 10.38592709** |
| miR395 | GUGAAGUGUUCGGAUCGCC | | 64 | 73994 | 2.8804 | 3640.002 | 10.30345436** |
| miR946 | UGUGGAAUAGAAGGGCUGAGU | | 0 | 255 | 0.01 | 12.5443 | 10.29281625** |
| miR2610 | AAGAUUUAGACAUUGUAUGGCGUU | | 7 | 2833 | 0.315 | 139.3643 | 8.7892935** |
| miR5774 | GCUUGGCCUACGACACGUGCAU | | 11 | 3200 | 0.4951 | 157.4183 | 8.3126676** |
| miR5540 | UUGUGCGAAUCAACGGUUCUA | | 14 | 3488 | 0.6301 | 171.5859 | 8.08913448** |
| miR3513 | UUGGAUAAGAUAGGAUUGGAU | | 1 | 234 | 0.045 | 11.5112 | 7.99889752** |
| miR812 | ACGGCACGAUUAAAGAUGGGCCAU | | 5 | 1041 | 0.225 | 51.2101 | 7.83035956** |
| miR3438 | UCAAGGAUUGAGAUAGAUAGC | | 13 | 2429 | 0.5851 | 119.4903 | 7.67399457** |
| miR5251 | AGUAGAUCAGUUGGAGCUU | | 2 | 329 | 0.09 | 16.1846 | 7.49048099** |
| miR5776 | ACUAUGGGCUGACCUUAGGUGG | | 11 | 1393 | 0.4951 | 68.5261 | 7.11278982** |
| miR6228 | GUGGAUAGUAGAAUGAUGAAGGA | | 3 | 349 | 0.135 | 17.1684 | 6.99065238** |
| miR5286 | ACAAUGGAGGCAAGGGAAGUA | | 30 | 2988 | 1.3502 | 146.9893 | 6.7663942** |
| miR529 | GAGGAAGGAGAGAUGGAGCAG | | 60 | 5700 | 2.7004 | 280.4013 | 6.6981761** |
| miR4387 | ACUGAGCACUGAUUGGAUGAUGAU | | 4 | 327 | 0.18 | 16.0862 | 6.48168285** |
| miR5181 | CUUUUUGGAUUGAAGGGA | | 4 | 309 | 0.18 | 15.2007 | 6.39999705** |
| miR8019 | AAAAGAAGACCCUUUUGAGCUUG | | 47 | 3599 | 2.1153 | 177.0463 | 6.3871206** |
| miR1168 | UCGUGGACAAGGCCAAGGCGC | | 19 | 1338 | 0.8551 | 65.8205 | 6.26630003** |
| miR2616 | AUUGGGUUGGGUCGGCCGGU | | 570 | 39850 | 25.6539 | 1960.3492 | 6.25578869** |
| miR6261 | AAGUGUUAGGUAUAGAGAAGCACG | | 7 | 484 | 0.315 | 23.8095 | 6.24004169** |
| miR4366 | CUUGUUGUAGAGUUUGUUGG | | 17 | 1154 | 0.7651 | 56.769 | 6.2133112** |
| miR8127 | CAACUGUGGGAGAUACCUUUA | | 60 | 3359 | 2.7004 | 165.24 | 5.93524603** |
| miR833 | UGAUUUGUUGAUAAUCGGCUCAGU | | 17 | 849 | 0.7651 | 41.765 | 5.7705023** |
| miR7510 | AGAACUGAGACUAUUAGCGGCGU | | 11 | 536 | 0.4951 | 26.3676 | 5.7349025** |
| miR1440 | UUAAGGAGAGGUUGGCUAUUUGAG | | 6 | 248 | 0.27 | 12.1999 | 5.4977661** |
| miR1160 | UGAAGGAAGCAGUAGUGGAU | | 11 | 413 | 0.4951 | 20.3168 | 5.35880943** |
| miR1512 | UAAGCAGAACAUUCAUGAGCAU | | 28 | 813 | 1.2602 | 39.9941 | 4.98806257** |
| miR5290 | AAUAUGAGUAGAGUAGACACCUA | | 57 | 1386 | 2.5654 | 68.1818 | 4.73213099** |
| miR5337 | CUAGAACGACGAAGACAUAUUGUGA | | 53 | 1264 | 2.3854 | 62.1802 | 4.70415214** |
| miR7758 | AACCGUUAGUUGACCGUGUAA | | 24 | 533 | 1.0802 | 26.22 | 4.60129733** |
| miR5304 | AUGAUGAGUCUGGUAAUUGGA | | 64 | 1350 | 2.8804 | 66.4108 | 4.5270768** |
| miR7785 | GUGAAUGGGUAGAGAGAGAAGAC | | 22 | 420 | 0.9902 | 20.6611 | 4.38305331** |
| miR1533 | AUAAUAAAAAUAGAUCAUGUG | | 18 | 335 | 0.8101 | 16.4797 | 4.34644616** |
| miR6426 | AGUGGAAGAUGGAAGUGUAGA | | 188 | 3403 | 8.4613 | 167.4045 | 4.30631516** |
| miR5037 | AUGAGAACUUUGAAGGCCGGC | | 1155 | 19027 | 51.9829 | 935.9991 | 4.17039812** |
| miR3520 | AGGUGAUCGGUGAAUAAUUAU | | 79 | 1257 | 3.5555 | 61.8359 | 4.1203203** |
| miR1026 | UGUGAAAUGACUUGAGAGGCC | | 121 | 1867 | 5.4458 | 91.8437 | 4.07596486** |
| miR4404 | ACAUGGAAGACUGGAUGGAUCAA | | 16 | 245 | 0.7201 | 12.0523 | 4.06496741** |
| miR5490 | UAUGGAUUUGUAUUUGGAUGG | | 63 | 921 | 2.8354 | 45.3069 | 3.9981066** |
| miR1869 | UGGAACAAUGUAGGCAAGGGAAGUA | | 2119 | 28170 | 95.3695 | 1385.7726 | 3.86101877** |
| miR6218 | CGAAAAAUCACGAAGACUUGC | | 30 | 393 | 1.3502 | 19.3329 | 3.83981303** |
| miR2916 | UGGGGGCUCGAAGACGAUCAGA | | 6410 | 70604 | 288.4938 | 3473.237 | 3.58966864** |
| miR5656 | AGUGAGUGAGAGAUUGGGUGU | | 21 | 213 | 0.9451 | 10.4782 | 3.47078011** |
| miR4371 | AUGGUGGUGACGGGUGACGGAGU | | 1823 | 17547 | 82.0475 | 863.1932 | 3.39515222** |
| miR5999 | CUUCACGAUCAUGACGGACAA | | 75 | 618 | 3.3755 | 30.4014 | 3.17096464** |
| miR3711 | GGCGCUAGAAGGAGGGCAUU | | 109 | 784 | 4.9057 | 38.5675 | 2.97485472** |
| miR5813 | ACAGCAGGACGGUGGUCAUGGA | | 3747 | 21623 | 168.6406 | 1063.7047 | 2.65707388** |
| miR7837 | UGGAUGGGAGGAUGUGGUGGU | | 49 | 265 | 2.2053 | 13.0362 | 2.56347656** |
| miR7485 | AAAGACCAUCUUUGAUUCGUUUGA | | 98 | 380 | 4.4107 | 18.6934 | 2.08344945** |
| miR1507 | UCUUCAUUCCACACGUCGUCU | | 70 | 255 | 3.1505 | 12.5443 | 1.99337925** |
| miR4414 | AGCUGCUGACUCGUUGGUUC | | 99 | 346 | 4.4557 | 17.0208 | 1.93357484** |
| miR5485 | UGACAAGUGGUAUCAGAGCAA | | 152 | 496 | 6.841 | 24.3998 | 1.83459019** |
| miR5807 | AGGACUCUAGGAGGUGAUGUGGC | | 319 | 1000 | 14.3572 | 49.1932 | 1.77668449** |
| miR398 | GGGGCGACAUGAGAUCACAUG | | 8029 | 22159 | 361.3598 | 1090.0722 | 1.59291577** |
|  |  | |  |  |  |  |  |
| ***Down-regulated miRNAs*** | | |  |  |  |  |  |
| miR4351 | | AUUGGGAGUGUGCAGUGGGAGUGG | 5736 | 0 | 258.1592 | 0.01 | -14.65597294** |
| miR5073 | | GAUUGGUGAACGGCAGAAGUAUUU | 2978 | 0 | 134.0303 | 0.01 | -13.71027147** |
| miR1135 | | UUCGCUCAAGUAAUUCUGACGGA | 2059 | 0 | 92.6691 | 0.01 | -13.17787312** |
| miR1516 | | AAAGUUGGGACUUGAGGAGGCGGU | 709 | 0 | 31.9098 | 0.01 | -11.63978408** |
| miR5574 | | UUUAAAUAGAACUCUAAAAGA | 657 | 0 | 29.5695 | 0.01 | -11.52989416** |
| miR4397 | | UGUCUAAGAGUGUGGCGACAUUAC | 464 | 0 | 20.8832 | 0.01 | -11.02812713** |
| miR5525 | | UCAAUCCUUGUGGAGACGAUCUGA | 418 | 0 | 18.8129 | 0.01 | -10.87750643** |
| miR6162 | | UUUGAUACGACUGUCAUUUACGG | 395 | 0 | 17.7777 | 0.01 | -10.79585287** |
| miR6138 | | CACGUUUGGGAUUGAGGUUGAAA | 372 | 0 | 16.7425 | 0.01 | -10.70929917** |
| miR6108 | | UAUGGGUGAGAAGGGAAGAUA | 33936 | 19 | 1527.3518 | 0.9347 | -10.67424131** |
| miR5025 | | AUCUGUAUAUAUGAGUAAUGAUCA | 337 | 0 | 15.1673 | 0.01 | -10.56674863** |
| miR4381 | | UUGUGACGGUCAACUGGUGAAAU | 334 | 0 | 15.0323 | 0.01 | -10.55385004** |
| miR7130 | | GUUUGGAAGUGUGGUGAGGUGGC | 328 | 0 | 14.7622 | 0.01 | -10.52769204** |
| miR7697 | | UCCGGAUAAAUUAUGAUCGGAUA | 299 | 0 | 13.457 | 0.01 | -10.39414106** |
| miR6032 | | UGGAGCAUGAAUCAGAAUCGG | 296 | 0 | 13.322 | 0.01 | -10.37959505** |
| miR1165 | | UACUGUAGCAAGCGGCCAUCC | 288 | 0 | 12.962 | 0.01 | -10.34007259** |
| miR3509 | | AUCUAACGACUGCUACAUAAUCAU | 278 | 0 | 12.5119 | 0.01 | -10.28908522** |
| miR5782 | | UAGCUGUGUAGUAGAAGUUGAGA | 278 | 0 | 12.5119 | 0.01 | -10.28908522** |
| miR5074 | | GCAAGGCCACCGUGCCGGCGACGC | 271 | 0 | 12.1969 | 0.01 | -10.25229873** |
| miR5516 | | CUGCAGUUGCUGUCGGGUAGGCGG | 268 | 0 | 12.0618 | 0.01 | -10.23622943** |
| miR782 | | ACAAACAGAGUUGGAUUUCUU | 249 | 0 | 11.2067 | 0.01 | -10.13014587** |
| miR5023 | | UUGGUAGUGAUAAAGGCGC | 239 | 0 | 10.7566 | 0.01 | -10.0710064** |
| miR7708 | | UGUCAUGAACUGAACGAAAGACAGC | 239 | 0 | 10.7566 | 0.01 | -10.0710064** |
| miR5780 | | AAACUUAACUGACGGUAGGGA | 2925 | 3 | 131.645 | 0.1476 | -9.80074424** |
| miR2863 | | AUAUAGGGACUAAAUGGGCAAA | 836 | 1 | 37.6257 | 0.0492 | -9.57884436** |
| miR6247 | | UGGCUGAAUGAACAUAAGGCA | 829 | 1 | 37.3107 | 0.0492 | -9.56671537** |
| miR444 | | UGCAUGUUAGUUGUGGCAAGCUU | 759 | 2 | 34.1602 | 0.0984 | -8.43944239** |
| miR779 | | UCUGCUCAUAGAUUGUCUGCUCAU | 603 | 2 | 27.1391 | 0.0984 | -8.10749886** |
| miR5561 | | CAUAAGAGAGAGAGCAUAGACAA | 457 | 2 | 20.5681 | 0.0984 | -7.70753451** |
| miR5259 | | CAAGGGGUAUUUGGAUGGACA | 617 | 3 | 27.7692 | 0.1476 | -7.5556491** |
| miR6145 | | CAUUGUUCACAUGUACGGCACUAU | 516 | 3 | 23.2235 | 0.1476 | -7.2977489** |
| miR7812 | | AUGUUAGGAAAUUGAUGGGUG | 887 | 6 | 39.9211 | 0.2952 | -7.07931495** |
| miR6475 | | UCUUGCGAGAGUAAAGAAUGA | 3233 | 23 | 145.5071 | 1.1314 | -7.00683666** |
| miR6248 | | AUGUUGUAGGAAUGGAGGUAGGUA | 990 | 9 | 44.5568 | 0.4427 | -6.65317244** |
| miR5373 | | UGUCUUGAUUUUAGAUGCAUG | 3543 | 47 | 159.4592 | 2.3121 | -6.10783973** |
| miR158 | | UUCAAAACUGUUAGACAAAGC | 364 | 5 | 16.3825 | 0.246 | -6.05735341** |
| miR5789 | | UGAUGAGCAUCUGGUCGGUAU | 289 | 4 | 13.007 | 0.1968 | -6.04641422** |
| miR5158 | | UGAGACGGGAUGAGAUGAGAU | 465 | 7 | 20.9282 | 0.3444 | -5.92521928** |
| miR6425 | | UUGCUUCCGUGGACAUAGGCA | 308 | 5 | 13.8621 | 0.246 | -5.81634371** |
| miR5721 | | AGAAAAUGGUAGAGAGAAAGUGGA | 346 | 6 | 15.5724 | 0.2952 | -5.72115477** |
| miR1149 | | CGGAUCAAUACUGACCCCCAGC | 392 | 7 | 17.6427 | 0.3444 | -5.67884241** |
| miR7712 | | UUCAAUUGUAGAAACUUUAGAUGGC | 333 | 7 | 14.9873 | 0.3444 | -5.44351155** |
| miR5818 | | UCGAACUGAGAGGCACAGGUU | 507 | 15 | 22.8185 | 0.7379 | -4.95063483** |
| miR5268 | | ACAAGUGGAAUGAGAUGGAUGGUU | 337 | 10 | 15.1673 | 0.4919 | -4.94645542** |
| miR7767 | | CCCAAGAUGAGUGCUCUCC | 322 | 14 | 14.4922 | 0.6887 | -4.39525714** |
| miR3437 | | AAAAAUACAAGGACUAAACGGAU | 892 | 39 | 40.1461 | 1.9185 | -4.38720917** |
| miR5830 | | AUGAGAGGAGGUGAUGUGACAUCA | 239 | 12 | 10.7566 | 0.5903 | -4.18762998** |
| miR5369 | | UGAGAAAGGAGAGAUGGUGCA | 16576 | 868 | 746.0332 | 42.6997 | -4.126942** |
| miR5519 | | UGGUAGACGCUACGGACUUAG | 1606 | 90 | 72.281 | 4.4274 | -4.02908484** |
| miR8143 | | GGGAUGGUGGAAAGAUGAUGGUA | 423 | 26 | 19.0379 | 1.279 | -3.89578618** |
| miR5256 | | AUAUAGGAUUAUGAAGAUUAA | 271 | 27 | 12.1969 | 1.3282 | -3.19897021** |
| miR5210 | | UAGGAUGUGUUUGGAAUUGAGGUU | 377 | 42 | 16.9676 | 2.0661 | -3.03780053** |
| miR1222 | | UUAAGAGAGGUCAGUGGUUCA | 421 | 52 | 18.9479 | 2.558 | -2.88894979** |
| miR5742 | | CCAAAUCAGAUGGUCGCGGAU | 453 | 56 | 20.3881 | 2.7548 | -2.88770785** |
| miR164 | | UGGAGAAGCAGGGCACGUGCA | 57341 | 9234 | 2580.7366 | 454.25 | -2.50622448** |
| miR5368 | | AGGGACAGUCUCAGGUAGA | 589 | 103 | 26.509 | 5.0669 | -2.38730699** |
| miR3946 | | GUGUAGAGAGAGAGAGAGAGACAC | 235 | 48 | 10.5766 | 2.3613 | -2.16322268** |
| miR5077 | | UUCACGUCGGGUUCACCA | 5365 | 1293 | 241.4616 | 63.6068 | -1.92454086** |
| miR156 | | UUGACAGAAGAGAGUGAGCAC | 475712 | 115344 | 21410.289 | 5674.141 | -1.91583036** |
| miR2118 | | GUCGAUGGAACAAUGUAGGCAAGG | 32284 | 9127 | 1453.0005 | 448.9864 | -1.69429155** |
| miR1023 | | AGCGAGAUUGGGAAAGUGCAU | 434 | 125 | 19.533 | 6.1492 | -1.66744291** |
| miR5380 | | GAGAAUGAGAUGGGGAUGGGGAA | 329 | 98 | 14.8072 | 4.8209 | -1.61892445** |
| miR535 | | UGACAAUGAGAGAGAGCACAC | 185022 | 57065 | 8327.2537 | 2807.2102 | -1.56870368** |
| miR5059 | | UCGUUCCUGGGCAGCAACACCA | 10610 | 3334 | 477.5225 | 164.0101 | -1.54178405** |

*** indicates a significant difference at P < 0.05 and P < 0.01.*

**Table S8 | List of Mg-deficiency-responsive novel miRNAs in *Citrus sinensis* leaves.**

| miRNA | *Sequence* | Expressed | | Normalized read count | | Fold change |
| --- | --- | --- | --- | --- | --- | --- |
|  |  | Control | Mg-deficiency | Control | Mg-deficiency |  |
| ***Up-regulated miRNAs*** | |  |  |  |  |  |
| novel_mir_96 | GTTCTCAGGTCGCCCCTGTGGGA | 0 | 11218 | 0.01 | 551.8494 | 15.75198699** |
| novel_mir_64 | TTTTGTTGCATGATGCTGATAA | 0 | 285 | 0.01 | 14.0201 | 10.45328092** |
|  |  |  |  |  |  |  |
| ***Down-regulated miRNAs*** | |  |  |  |  |  |
| novel_mir_243 | TCCCTACTCCACCCATGCCATA | 1926 | 0 | 86.6832 | 0.01 | -13.08153679** |
| novel_mir_252 | TTGAGCCGCGTCAATATCTCC | 579 | 0 | 26.059 | 0.01 | -11.34756593** |
| novel_mir_250 | TAGATAACGGATTAACGGCTA | 368 | 0 | 16.5625 | 0.01 | -10.6937047** |
| novel_mir_233 | AGCAGGAAAGTGGCTGGTTGA | 309 | 0 | 13.9071 | 0.01 | -10.44160584** |
| novel_mir_132 | GTGACAGAAGATAGAGAGCGC | 24996 | 2801 | 1124.9907 | 137.7902 | -3.02936789** |
| novel_mir_188 | TACAAACTGACGTGGCATGAT | 5479 | 1227 | 246.5924 | 60.3601 | -2.03046124** |
| novel_mir_71 | TTGACATGTGCACAGTCGGAC | 297 | 84 | 13.367 | 4.1322 | -1.69369372** |

*** indicates a significant difference at P < 0.05 and P < 0.01.*

| **Table S9:** List of target genes for parts of known miRNAs in *Citrus sinensis* leaves | | | |
| --- | --- | --- | --- |
| **miRNA** | **Assession** | **Homology** | **Target genes** |
| miR1851 | orange1.1g034260m | AT5G51970.2 | GroES-like zinc-binding alcohol dehydrogenase family protein |
|  | orange1.1g016177m | AT1G49520.1 | SWIB complex BAF60b domain-containing protein |
|  | orange1.1g004374m | AT2G41770.1 | Protein of unknown function (DUF288) |
|  | orange1.1g002218m | AT4G20910.1 | Double-stranded RNA binding protein-related / DsRBD protein-related |
|  | orange1.1g033660m |  |  |
|  | orange1.1g034260m | AT5G51970.2 | GroES-like zinc-binding alcohol dehydrogenase family protein |
|  | orange1.1g002226m | AT4G20910.2 | Double-stranded RNA binding protein-related / DsRBD protein-related |
| miR3443 | orange1.1g017888m | AT5G12040.1 | Nitrilase/cyanide hydratase and apolipoprotein N-acyltransferase family protein |
|  | orange1.1g022660m | AT5G12040.2 | Nitrilase/cyanide hydratase and apolipoprotein N-acyltransferase family protein |
| miR5821 | orange1.1g045278m | AT1G33060.2 | NAC 014 |
|  | orange1.1g022991m | AT1G71190.1 | Senescence associated gene 18 |
|  | orange1.1g013216m | AT4G38220.2 | Peptidase M20/M25/M40 family protein |
|  | orange1.1g013368m | AT4G38220.1 | Peptidase M20/M25/M40 family protein |
|  | orange1.1g046783m | AT2G04620.1 | Cation efflux family protein |
| miR5832 | orange1.1g037540m | AT4G16640.1 | Matrixin family protein |
| miR6233 | orange1.1g035332m |  |  |
| miR946 | orange1.1g005467m | AT1G70610.1 | Transporter associated with antigen processing protein 1 |
| miR5181 | orange1.1g000918m | AT4G34310.1 | alpha/beta-Hydrolases superfamily protein |
|  | orange1.1g046910m | AT1G19260.1 | TTF-type zinc finger protein with HAT dimerisation domain |
|  | orange1.1g030522m | AT5G16110.1 |  |
|  | orange1.1g047186m | AT1G19260.1 | TTF-type zinc finger protein with HAT dimerisation domain |
|  | orange1.1g043961m | AT3G18990.1 | AP2/B3-like transcriptional factor family protein |
|  | orange1.1g008441m | AT5G60250.1 | Zinc finger (C3HC4-type RING finger) family protein |
|  | orange1.1g006461m | AT2G03890.1 | Phosphoinositide 4-kinase gamma 7 |
|  | orange1.1g013377m | AT2G17760.1 | Eukaryotic aspartyl protease family protein |
|  | orange1.1g038130m | AT4G12010.1 | Disease resistance protein (TIR-NBS-LRR class) family |
|  | orange1.1g009558m | AT3G49720.1 |  |
|  | orange1.1g010497m | AT2G17840.1 | Senescence/dehydration-associated protein-related |
|  | orange1.1g046634m | AT1G19260.1 | TTF-type zinc finger protein with HAT dimerisation domain |
|  | orange1.1g017007m | AT1G70150.1 | Zinc ion binding |
|  | orange1.1g044415m | AT1G73850.1 | Protein of unknown function (DUF1666) |
|  | orange1.1g007728m | AT2G14960.1 | Auxin-responsive GH3 family protein |
|  | orange1.1g020776m | AT3G08950.1 | Electron transport SCO1/SenC family protein |
|  | orange1.1g040037m | AT1G19260.1 | TTF-type zinc finger protein with HAT dimerisation domain |
|  | orange1.1g004845m | AT5G55930.1 | Oligopeptide transporter 1 |
|  | orange1.1g038168m | AT5G60900.1 | Receptor-like protein kinase 1 |
|  | orange1.1g011110m | AT5G38840.1 | SMAD/FHA domain-containing protein |
|  | orange1.1g043340m | AT2G39670.2 | Radical SAM superfamily protein |
| miR1160 | orange1.1g005451m | AT4G11440.1 | Mitochondrial substrate carrier family protein |
|  | orange1.1g004285m | AT3G23430.1 | Phosphate 1 |
| miR4366 | orange1.1g008795m | AT1G71800.1 | Cleavage stimulating factor 64 |
|  | orange1.1g020626m | AT3G21360.1 | 2-oxoglutarate (2OG) and Fe(II)-dependent oxygenase superfamily protein |
|  | orange1.1g015258m | AT3G12250.2 | TGACG motif-binding factor 6 |
|  | orange1.1g021676m | AT3G12250.1 | TGACG motif-binding factor 6 |
|  | orange1.1g026397m | AT2G36110.1 | Polynucleotidyl transferase, ribonuclease H-like superfamily protein |
|  | orange1.1g019129m | AT4G21960.1 | Peroxidase superfamily protein |
|  | orange1.1g001962m | AT3G01780.1 | ARM repeat superfamily protein |
| miR833 | orange1.1g047519m | AT1G45616.1 | Receptor like protein 6 |
| miR1533 | orange1.1g007444m | AT1G08520.1 | ALBINA 1 |
| miR1168 | orange1.1g008187m | AT1G58100.1 | TCP family transcription factor |
| miR5656 | orange1.1g023027m | AT2G03050.1 | Mitochondrial transcription termination factor family protein |
|  | orange1.1g030403m | AT4G30930.1 | Ribosomal protein L21 |
| miR1077 | orange1.1g014749m | AT2G34250.1 | SecY protein transport family protein |
| miR7785 | orange1.1g030776m | AT5G19440.1 | NAD(P)-binding Rossmann-fold superfamily protein |
| miR1512 | orange1.1g017846m | AT1G32790.1 | CTC-interacting domain 11 |
| miR6218 | orange1.1g007885m | AT3G12010.1 |  |
|  | orange1.1g013847m | AT3G12010.1 |  |
|  | orange1.1g005203m | AT1G70610.1 | Transporter associated with antigen processing protein 1 |
|  | orange1.1g003633m | AT2G32400.1 | Glutamate receptor 5 |
| miR8019 | orange1.1g010016m | AT4G27500.1 | Proton pump interactor 1 |
| miR7837 | orange1.1g023148m | AT2G35700.1 | ERF family protein 38 |
|  | orange1.1g019677m | AT3G48120.1 |  |
| miR5337 | orange1.1g011353m | AT1G63500.1 | Protein kinase protein with tetratricopeptide repeat domain |
|  | orange1.1g017395m | AT5G41260.1 | Protein kinase protein with tetratricopeptide repeat domain |
|  | orange1.1g005610m | AT3G44830.1 | Lecithin:cholesterol acyltransferase family protein |
| miR529 | orange1.1g026986m | AT2G36895.1 |  |
|  | orange1.1g006213m | AT1G49890.1 | Family of unknown function (DUF566) |
|  | orange1.1g042284m | AT4G21990.1 | APS reductase 3 |
|  | orange1.1g005441m | AT1G48110.2 | Evolutionarily conserved C-terminal region 7 |
|  | orange1.1g005453m | AT1G48110.1 | Evolutionarily conserved C-terminal region 7 |
|  | orange1.1g006668m | AT1G15530.1 | Concanavalin A-like lectin protein kinase family protein |
|  | orange1.1g008238m | AT4G05200.1 | Cysteine-rich RLK (RECEPTOR-like protein kinase) 25 |
|  | orange1.1g004813m | AT5G64320.1 | Pentatricopeptide repeat (PPR) superfamily protein |
| miR5490 | orange1.1g023612m | AT1G04945.2 | HIT-type Zinc finger family protein |
| miR395 | orange1.1g005583m | AT3G02050.1 | K^+^ uptake transporter 3 |
|  | orange1.1g014749m | AT2G34250.1 | SecY protein transport family protein |
| miR1507 | orange1.1g034576m | AT3G14470.1 | NB-ARC domain-containing disease resistance protein |
|  | orange1.1g042037m | AT3G14460.1 | LRR and NB-ARC domains-containing disease resistance protein |
|  | orange1.1g046115m | AT3G14470.1 | NB-ARC domain-containing disease resistance protein |
|  | orange1.1g035555m | AT3G14470.1 | NB-ARC domain-containing disease resistance protein |
|  | orange1.1g002154m | AT3G14470.1 | NB-ARC domain-containing disease resistance protein |
|  | orange1.1g039822m | AT3G14470.1 | NB-ARC domain-containing disease resistance protein |
|  | orange1.1g045522m | AT3G50950.2 | HOPZ-ACTIVATED RESISTANCE 1 |
| miR5485 | orange1.1g034260m | AT5G51970.2 | GroES-like zinc-binding alcohol dehydrogenase family protein |
| miR6426 | orange1.1g037454m | AT5G60020.1 | Laccase 17 |
|  | orange1.1g010327m | AT5G24120.1 | Sigma factor E |
| miR2616 | orange1.1g032059m | AT5G19590.1 | Protein of unknown function, DUF538 |
| miR5037 | orange1.1g017170m | AT5G65670.2 | Indole-3-acetic acid inducible 9 |
| miR1869 | orange1.1g023864m | AT4G25720.1 | Glutaminyl cyclase |
| miR2916 | orange1.1g010447m | AT1G51980.1 | Insulinase (Peptidase family M16) protein |
| miR164 | orange1.1g030909m | AT1G56010.2 | NAC domain containing protein 1 |
|  | orange1.1g047710m | AT5G53950.1 | NAC (No Apical Meristem) domain transcriptional regulator superfamily protein |
|  | orange1.1g017636m | AT3G08030.1 | Protein of unknown function, DUF642 |
|  | orange1.1g022869m | AT3G08030.2 | Protein of unknown function, DUF642 |
|  | orange1.1g017827m | AT5G61430.1 | NAC domain containing protein 100 |
| miR535 | orange1.1g009840m | AT5G24910.1 | Cytochrome P450, family 714, subfamily A, polypeptide 1 |
| miR156 | orange1.1g029650m | AT1G53160.1 | Squamosa promoter binding protein-like 4 |
|  | orange1.1g030599m | AT3G60030.1 | Squamosa promoter-binding protein-like 12 |
|  | orange1.1g009653m | AT1G69170.1 | Squamosa promoter-binding protein-like (SBP domain) transcription factor family protein |
|  | orange1.1g011640m | AT5G43270.2 | Squamosa promoter binding protein-like 2 |
|  | orange1.1g011651m | AT5G43270.3 | Squamosa promoter binding protein-like 2 |
|  | orange1.1g021420m | AT5G50670.1 | Squamosa promoter-binding protein-like (SBP domain) transcription factor family protein |
|  | orange1.1g032310m | AT2G33810.1 | Squamosa promoter binding protein-like 3 |
|  | orange1.1g008680m | AT1G69170.1 | Squamosa promoter-binding protein-like (SBP domain) transcription factor family protein |
|  | orange1.1g032937m | AT3G15270.1 | Squamosa promoter binding protein-like 5 |
|  | orange1.1g046416m | AT2G42200.1 | Squamosa promoter binding protein-like 9 |
|  | orange1.1g016971m | AT5G50570.2 | Squamosa promoter-binding protein-like (SBP domain) transcription factor family protein |
| miR3946 | orange1.1g025742m | AT1G21000.2 | PLATZ transcription factor family protein |
|  | orange1.1g026068m | AT1G21000.1 | PLATZ transcription factor family protein |
|  | orange1.1g005518m | AT2G35940.1 | BEL1-like homeodomain 1 |
|  | orange1.1g021412m | AT5G66530.2 | Galactose mutarotase-like superfamily protein |
|  | orange1.1g016506m | AT1G32740.1 | SBP (S-ribonuclease binding protein) family protein |
|  | orange1.1g018220m | AT3G56680.1 | Single-stranded nucleic acid binding R3H protein |
|  | orange1.1g011991m | AT2G43850.1 | Integrin-linked protein kinase family |
|  | orange1.1g017665m | AT3G04070.1 | NAC domain containing protein 47 |
|  | orange1.1g016142m | AT2G01170.1 | Bidirectional amino acid transporter 1 |
|  | orange1.1g002698m | AT2G42600.1 | Phosphoenolpyruvate carboxylase 2 |
|  | orange1.1g002776m | AT3G22400.1 | PLAT/LH2 domain-containing lipoxygenase family protein |
|  | orange1.1g025497m | AT5G65430.1 | General regulatory factor 8 |
|  | orange1.1g030941m | AT3G16640.1 | Translationally controlled tumor protein |
|  | orange1.1g025914m | AT4G13040.1 | Integrase-type DNA-binding superfamily protein |
|  | orange1.1g011758m | AT5G55860.1 | Plant protein of unknown function (DUF827) |
|  | orange1.1g006091m | AT5G24300.2 | Glycogen/starch synthases, ADP-glucose type |
|  | orange1.1g009139m | AT5G24300.1 | Glycogen/starch synthases, ADP-glucose type |
|  | orange1.1g007773m | AT5G24240.1 | Phosphatidylinositol 3- and 4-kinase ;Ubiquitin family protein |
|  | orange1.1g024507m | AT5G32450.1 | RNA binding (RRM/RBD/RNP motifs) family protein |
|  | orange1.1g013752m | AT2G46810.1 | Basic helix-loop-helix (bHLH) DNA-binding superfamily protein |
|  | orange1.1g014958m | AT3G61950.1 | Basic helix-loop-helix (bHLH) DNA-binding superfamily protein |
|  | orange1.1g010449m | AT5G08570.1 | Pyruvate kinase family protein |
| miR5023 | orange1.1g009492m | AT4G12300.1 | Cytochrome P450, family 706, subfamily A, polypeptide 4 |
| miR5789 | orange1.1g025015m | AT5G49400.1 | Zinc knuckle (CCHC-type) family protein |
| miR7697 | orange1.1g005619m | AT1G74190.1 | Receptor like protein 15 |
|  | orange1.1g037792m | AT1G07390.2 | Receptor like protein 1 |
|  | orange1.1g042610m | AT1G74180.1 | Receptor like protein 14 |
| miR6425 | orange1.1g035520m | AT4G10660.1 | CDC68-related |
|  | orange1.1g030905m | AT4G36020.1 | Cold shock domain protein 1 |
| miR7767 | orange1.1g031715m | AT3G52560.1 | Ubiquitin E2 variant 1D-4 |
|  | orange1.1g033889m | AT2G47900.3 | Tubby like protein 3 |
|  | orange1.1g016567m | AT2G47900.1 | Tubby like protein 3 |
|  | orange1.1g009727m | AT4G20860.1 | FAD-binding Berberine family protein |
| miR5380 | orange1.1g022661m | AT1G09660.1 | RNA-binding KH domain-containing protein |
| miR158 | orange1.1g002569m | AT5G63020.1 | Disease resistance protein (CC-NBS-LRR class) family |
|  | orange1.1g038105m | AT1G12220.1 | Disease resistance protein (CC-NBS-LRR class) family |
|  | orange1.1g041843m | AT1G12280.1 | LRR and NB-ARC domains-containing disease resistance protein |
| miR5210 | orange1.1g029519m | AT5G44080.1 | Basic-leucine zipper (bZIP) transcription factor family protein |
| miR1149 | orange1.1g008466m | AT5G50320.1 | Radical SAM domain-containing protein / GCN5-related N-acetyltransferase (GNAT) family protein |
| miR1222 | orange1.1g016962m | AT5G40990.1 | GDSL lipase 1 |
|  | orange1.1g011822m | AT2G34250.1 | SecY protein transport family protein |
|  | orange1.1g017447m | AT2G34250.2 | SecY protein transport family protein |
|  | orange1.1g003640m | AT2G32400.1 | Glutamate receptor 5 |
|  | orange1.1g002536m | AT2G46920.2 | Protein phosphatase 2C family protein |
|  | orange1.1g002806m | AT2G46920.1 | Protein phosphatase 2C family protein |
| miR5818 | orange1.1g001860m | AT4G27190.1 | NB-ARC domain-containing disease resistance protein |
| miR5368 | orange1.1g011456m | AT5G53840.1 | F-box/RNI-like/FBD-like domains-containing protein |
|  | orange1.1g013011m | AT4G01050.1 | Thylakoid rhodanese-like |
| miR779 | orange1.1g027903m | AT3G63120.1 | Cyclin p1;1 |
|  | orange1.1g044779m | AT2G38290.1 | Ammonium transporter 2 |
|  | orange1.1g042791m | AT3G14470.1 | NB-ARC domain-containing disease resistance protein |
|  | orange1.1g003190m | AT1G29370.1 | Kinase-related protein of unknown function (DUF1296) |
|  | orange1.1g041074m | AT2G38290.1 | Ammonium transporter 2 |
|  | orange1.1g001921m | AT3G45630.1 | RNA binding (RRM/RBD/RNP motifs) family protein |
|  | orange1.1g025347m | AT2G40610.1 | Expansin A8 |
|  | orange1.1g045028m | AT1G73660.1 | Protein tyrosine kinase family protein |
|  | orange1.1g022465m | AT2G35260.1 |  |
|  | orange1.1g022454m | AT2G35260.1 |  |
| miR5574 | orange1.1g037861m | AT1G47980.1 |  |
| miR7812 | orange1.1g017621m | AT4G08850.1 | Leucine-rich repeat receptor-like protein kinase family protein |
|  | orange1.1g045556m | ATCG00190.1 | RNA polymerase subunit beta |
|  | orange1.1g038769m | AT3G24503.1 | Aldehyde dehydrogenase 2C4 |
|  | orange1.1g011499m | AT1G72520.1 | PLAT/LH2 domain-containing lipoxygenase family protein |
| miR5519 | orange1.1g016864m | AT4G39520.1 | GTP-binding protein-related |
|  | orange1.1g009752m | AT4G24490.1 | RAB geranylgeranyl transferase alpha subunit 1 |
| miR5077 | orange1.1g006793m | AT1G25570.1 | Di-glucose binding protein with Leucine-rich repeat domain |
|  | orange1.1g036785m | AT2G32990.1 | glycosyl hydrolase 9B8 |
|  | orange1.1g015573m | AT5G11860.3 | SCP1-like small phosphatase 5 |
|  | orange1.1g048613m | AT4G16260.1 | Glycosyl hydrolase superfamily protein |
| miR5742 | orange1.1g009718m | AT5G20890.1 | TCP-1/cpn60 chaperonin family protein |
|  | orange1.1g041155m | AT2G34930.1 | Disease resistance family protein/LRR family protein |
|  | orange1.1g023573m | AT1G52340.1 | NAD(P)-binding Rossmann-fold superfamily protein |
| miR5561 | orange1.1g018677m | AT4G36730.1 | G-box binding factor 1 |
|  | orange1.1g019071m | AT4G36730.2 | G-box binding factor 1 |
| miR5158 | orange1.1g043878m | AT5G37930.1 | Protein with RING/U-box and TRAF-like domains |
|  | orange1.1g042649m | AT4G21330.1 | Basic helix-loop-helix (bHLH) DNA-binding superfamily protein |
| miR5256  miR8127 | orange1.1g004233m | AT4G35790.2 | Phospholipase D delta |
|  | orange1.1g037313m | AT1G73066.1 | Leucine-rich repeat family protein |
| miR1172 | orange1.1g019097m | AT1G68620.1 | alpha/beta-Hydrolases superfamily protein |
|  | orange1.1g003176m | AT1G64260.1 | MuDR family transposase |

**Table S10 | List of target genes for parts of novel miRNAs in *Citrus sinensis* leaves.**

| **miRNA** | **Assession** | **Homology** | **Target genes** |
| --- | --- | --- | --- |
| novel_mir_243 | orange1.1g037138m | AT5G63020.1 | Disease resistance protein (CC-NBS-LRR class) family |
| novel_mir_233 | orange1.1g045509m | AT1G69310.2 | WRKY DNA-binding protein 57 |
| novel_mir_132 | orange1.1g029650m | AT1G53160.1 | Squamosa promoter binding protein-like 4 |
|  | orange1.1g030599m | AT3G60030.1 | Squamosa promoter-binding protein-like 12 |
|  | orange1.1g009653m | AT1G69170.1 | Squamosa promoter-binding protein-like (SBP domain) transcription factor family protein |
|  | orange1.1g010591m | AT1G69170.1 | Squamosa promoter-binding protein-like (SBP domain) transcription factor family protein |
|  | orange1.1g010621m | AT1G69170.1 | Squamosa promoter-binding protein-like (SBP domain) transcription factor family protein |
|  | orange1.1g010605m | AT1G69170.1 | Squamosa promoter-binding protein-like (SBP domain) transcription factor family protein |
|  | orange1.1g010865m | AT1G69170.1 | Squamosa promoter-binding protein-like (SBP domain) transcription factor family protein |
|  | orange1.1g010873m | AT1G69170.1 | Squamosa promoter-binding protein-like (SBP domain) transcription factor family protein |
|  | orange1.1g011640m | AT5G43270.2 | Squamosa promoter binding protein-like 2 |
|  | orange1.1g011651m | AT5G43270.3 | Squamosa promoter binding protein-like 2 |
|  | orange1.1g011646m | AT5G43270.1 | Squamosa promoter binding protein-like 2 |
|  | orange1.1g011637m | AT5G43270.3 | Squamosa promoter binding protein-like 2 |
|  | orange1.1g011635m | AT5G43270.2 | Squamosa promoter binding protein-like 2 |
|  | orange1.1g011662m | AT5G43270.3 | Squamosa promoter binding protein-like 2 |
|  | orange1.1g013094m | AT5G43270.1 | Squamosa promoter binding protein-like 2 |
|  | orange1.1g016391m | AT5G43270.3 | Squamosa promoter binding protein-like 2 |
|  | orange1.1g016364m | AT5G43270.1 | Squamosa promoter binding protein-like 2 |
|  | orange1.1g021420m | AT5G50670.1 | Squamosa promoter-binding protein-like (SBP domain) transcription factor family protein |
|  | orange1.1g032310m | AT2G33810.1 | Squamosa promoter binding protein-like 3 |
|  | orange1.1g046416m | AT2G42200.1 | Squamosa promoter binding protein-like 9 |
|  | orange1.1g017256m | AT2G42200.1 | Squamosa promoter binding protein-like 9 |
|  | orange1.1g016971m | AT5G50570.2 | Squamosa promoter-binding protein-like (SBP domain) transcription factor family protein |

**
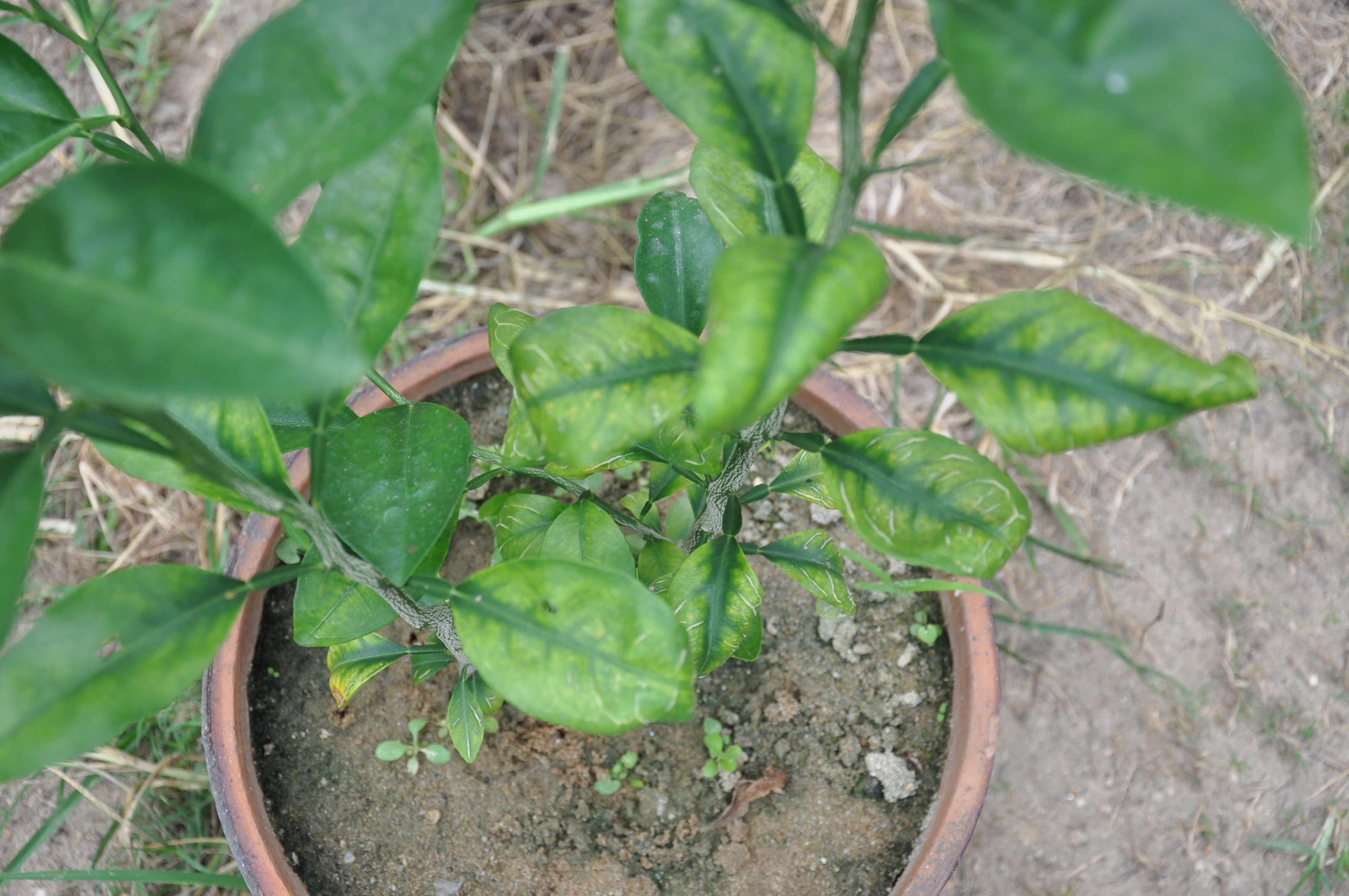
**

**Figure S1 | Fig. 1 Mg-deficient symptoms in *Citrus sinensis* seedlings.**

**Figure S2 | Length distribution of small RNAs from Mg-sufficient and -deficient *Citrus sinensis* leaves.**
